# Supplementary material for: Reprogrammed SimCells for antimicrobial therapy
Source: Proc Natl Acad Sci U S A. 2026 Mar 17;123(12):e2517118123. doi: 10.1073/pnas.2517118123 (PMC13012131; doi:10.1073/pnas.2517118123)
Supplement: Supplementary file 1 — Appendix 01 (PDF) [file pnas.2517118123.sapp.pdf]

Supplementary Information

Reprogrammed SimCells for Antibacterial Therapy

Yun Dong[1], Xianglin Ji[2], Tao Dong[3], Yun Wang[2], Erik Bakkeren[4,5], Kevin R. Foster[4] and Wei E. Huang[1*].

1. Department of Engineering Science, University of Oxford, Parks Road, OX1 3PJ, Oxford, UK.
2. Oxford Suzhou Centre for Advanced Research (OSCAR), University of Oxford, Suzhou Industrial Park, Jiangsu, P.R. China.
3. Department of Immunology and Microbiology, School of Life Sciences, Southern University of Science and Technology, Shenzhen 518055, China.
4. Sir William Dunn School of Pathology, University of Oxford, Oxford OX1 3RE, UK
5. Department of Biological Sciences, University of Calgary, Calgary T2N 1N4, Canada

*Corresponding author
Wei E. Huang
Tel.: +44 1865 283789
Email: wei.huang@eng.ox.ac.uk

Classification: Biological Sciences, Microbiology

Keywords: synthetic biology, SimCells, mini-SimCells, nanobody-antigen interaction, antibacterial therapy, type VI secretion system, aspirin, catechol, hydrogen peroxide, antimicrobial resistance

Supplementary Results

Heterologous Expression, Assembly and Function of T6SS in E. coli

The expression, assembly and function of T6SS were verified in *E. coli* BL21(DE3) parental cells. Following the confirmation of heterologous T6SS expression with fluorescence analysis, a bacterial killing assay was conducted to assess the functional efficacy of the T6SS in contact-promoting conditions (as in solid growth conditions). After a 4-hour co-incubation on the LB plate, both constitutive and induced T6SS-expressing attacker cells demonstrated significant killing activity against prey cells compared to the inactive controls. Notably, the induced T6SS expression resulted in the elimination of over 98% of the prey cells in the mixture (Supplementary Figure S2a). These findings confirm the functional efficacy of heterologously expressed T6SS.

To examine the stability of T6SS machinery, flow cytometry analysis was conducted to monitor the fluorescence signals of sfGFP-labelled T6SS VipA sheath in constitutive and induced T6SS-expressing strains over a 96-hour time course. For the constitutive T6SS-expressing strain, over 97% of cells remained GFP^+ after 96 hours of continuous culture, and no significant decrease was observed in the duration. Notably, the proportion of GFP^+ cells in the induced T6SS-expressing strain increased progressively over time with the presence of the inducer in the culturing medium. However, there were fewer GFP^+ cells in the induced T6SS-expressing strain (~65%) compared to the constitutive group (Supplementary Figure S2b). To further assess machinery functionality, we evaluated the competitive capacity of both attacker strains against a prey strain (*E. coli* DH10B) after 96 hours of growth. Significant out-competition of the prey cells was observed in both groups, with the constitutive T6SS-expressing strain exhibiting a higher inhibition efficiency (~46% elimination of prey cells) compared to the induced group (~37% elimination of prey cells) (Supplementary Figure S2c). The results suggest a higher stability for T6SS under constitutive control, possibly due to a relatively lower burden for cell growth. A decline of the T6SS functionality was noticed after 96-hour culturing without a loss in the GFP^+ cell population, which may be attributed to the decrease in cell viability due to an insufficient energy supply.

Characterisation of Reprogrammed SimCells in High Purity

The reprogrammed SimCells were generated by inducing the expression of I-CeuI endonuclease in the reprogrammed SimCell-generating parental cells(1, 2). After the culture reached the early log phase (OD_{600} ~ 0.4), 1 μM of crystal violet was added to induce the I-

CeuI expression. Growth arrest was observed within 1 hour for SimCell (+) groups, resulting in a stable OD_{600} plateau maintained for over 35 hrs of continuous culturing, while the uninduced or control groups continued normal growth until reaching the stationary phase (Supplementary Figure S4a). For further verification, 5 μL of post-35-hr cultures were plated on LB agar, and no single colony formation was detected after 48 hrs incubation for induced SimCells (Supplementary Figure S4a). Additionally, as a second validation, we stained cells with SYTO 9, a DNA-binding fluorescent dye. As expected, no significant fluorescence was observed in SimCells compared to the control group (parental cells) (Supplementary Figure S4b). Single-cell Raman spectra analysis revealed the absence of the Raman band at 785 cm^{-1} in SimCells (Supplementary Figure S4c). Both above evidence confirm the removal of genome DNA. Finally, plating 200 μL of SimCell culture at OD_{600} = 2.0 (~10^8 cells) yielded no single colony formation after 24 hours of incubation (Supplementary Figure S4d). These results confirm the high purity of the reprogrammed SimCells, which yield an escape frequency of less than 10^{-8}, meeting the criteria of NIH guidelines for recombinant microorganisms(3).

T6SS Expression and Activity in Reprogrammed SimCells

The expression, assembly and function of T6SS were further verified in reprogrammed SimCells. Under the microscope, dotted GFP signals were identified as a characteristic mark for T6SS activity in most SimCells (Supplementary Figure S5a). SimCells with constitutive and tetracycline-induced T6SS were generated from the cultures with the same cell density. A higher yield of functional SimCells was usually obtained for the constitutive T6SS group. The constitutive T6SS plasmid (pT6S_NP/pT6S_N3-NP) allowed the parental cells to synthesise and assemble an active secretion system before chromosome removal, reducing metabolic burden and yielding more functional T6SS SimCells than an inducible plasmid (pT6S_Tet/pT6S_N3-Tet). Hence, SimCells carrying the constitutive T6SS plasmid were used in subsequent experiments. On agar media, the T6SS activity showed significant killing effect on prey cells within 3 hrs (Supplementary Figure S5b). Nonetheless, SimCell killing efficiency was lower than that of the parental bacteria, likely due to their limited residual energy reserves.

Hydrogen Peroxide (H_2O_2) Produced from Catechol Polymerisation Shows Broad-spectrum Antimicrobial Activities

Catechol is a broad-spectrum antimicrobial candidate (4–6). Through an auto-polymerisation process, H_2O_2 is generated and subsequently disrupts a broad range of bacterial cellular processes (Figure 4a) (7–10). A hydrogen peroxide assay with 800 μM catechol in M9 minimal

medium showed sustained H_2O_2 release over 21 days, reaching concentrations above 300 μM (Supplementary Figure S7a). In the disc diffusion assay, the formation of inhibition zones verified the antibacterial activity of 5 mM to 100 mM catechol against *Pseudomonas putida* (Supplementary Figure S7b). Additionally, when different concentrations of catechol were added to *E. coli* DH10B cultures, significant inhibition was observed at 5 mM and higher concentrations, with a time-dependent increase in killing efficiency for the 5 mM group (Supplementary Figure S7c). This indicates a prolonged release of H_2O_2 from catechol polymerisation, contributing to sustained antibacterial activity over time.

Catechol is Generated and Released from Parental Cells and SimCells

To achieve a sustained cellular conversion of aspirin to catechol, the NahG enzyme was integrated into our engineered parental cell, SimCell and mini-SimCell platforms, regulated by a strong constitutive promoter J23100 followed by a strong ribosome binding site (RBS) BBa_B0031. The NahG enzyme and mRFP labelling were positioned downstream of the nanobody (Supplementary Figure S8a). As for the negative control (NC) strain, SalR, a regulatory protein for a salicylate hydroxylase SalA, which shares high function similarity with NahG, is used as substitution of NahG.

With 800 μM aspirin added into the NahG+ parental cells, the filtered supernatant from the overnight culture showed a dark-brown colour (Supplementary Figure S8b), indicating the cell-produced catechol was permeable to the cell membrane and was released to the surrounding environment. To test the antimicrobial effect, the collected supernatants were subsequently mixed in varying ratios with concentrated LB medium to normalise the nutritional content. These supernatant-LB mixtures were then utilised as the growth medium for the prey cells (*E. coli* DH10B). As shown in Supplementary Figure S7b, the medium containing 20% NahG+ supernatant demonstrated a significant inhibitory effect compared to the control group. The cell growth was reduced in mixtures with 50% and 80% supernatant, likely due to the accumulation of secondary metabolites from prior culturing. Similar results were observed using supernatant from NahG+ SimCells (Supplementary Figure S7c). This result corroborates that catechol is permeable across the cell membrane to exert its antimicrobial activity.

Supplementary Methods

Assessing T6SS machinery stability with flow cytometry

Flow cytometry was applied to track the T6SS-related fluorescence in a single cell. Overnight cultures were 1:100 re-inoculated into the fresh LB medium with antibiotics for selection. All cultures were continuously incubated at 37°C, 180 rpm. Samples were collected at 24, 48, 72, 96h, centrifuged and resuspended in 1×PBS. A FACS Calibur (BD Biosciences) with an FL1 filter (FITC channel) was used for flow cytometry analysis. CellQuest was used to acquire 100,000 viable cells' fluorescence data for each experiment. Data analysis was performed by FlowJo software.

SYTO 9 staining assay

200 μL of culture from each group were collected, washed twice then resuspended with 1×PBS solution. A final concentration of 100nM of SYTO 9 stain was added and the cultures were incubated in a dark place at room temperature for 60 min. Cell pellets were collected and washed with 1 mL sterile ddH_2O three times to remove the dye. 2 μL of the sample was used for fluorescence microscopy. Excitation/emission for SYTO 9 stain was 485/498 nm.

Single-cell Raman Spectra (SCRS)

Raman microspectroscopy was used for the characterisation of single bacterial cells' metabolic profiles. Prior to the measurement, the SimCells were washed with sterile ddH_2O three times to remove the culture medium and extracellular metabolites and then diluted until individual cells could be observed under the microscope. 2 μL of each sample was dropped and air-dried onto an aluminium-coated slide. Raman spectroscopic acquisition was performed using a LabRAM HR Evolution confocal Raman microscope with a 100×/0.75 air objective (HORIBA, UK). A 532-nm neodymium-yttrium aluminium garnet laser with a 300-grooves mm^{-1} diffraction grating was used for SCRS. ~80 mW laser power was set, and before focusing, it was attenuated by neutral density (ND) filters to protect the samples. 100–3200 cm^{-1} spectrum was obtained and recorded by LABSPEC 6 software (HORIBA, UK). All raw spectra were corrected using cosmic ray correction, pre-processed using polyline baseline fitting and subtraction, and vector-normalised for the entire spectrum. For optimising single-cell level visualisation, Linear discriminant analysis (LDA) was applied for SCRS dimension reduction. R studio was used for data analysis and figure plotting. Raman peak at 785 cm^{-1} (cytosine ring breathing mode) indicated the DNA/RNA level of the single cell.

Fluorescence Microscopy

Prepared fresh parental cell or purified SimCell / mini-SimCell samples were washed twice and then resuspended with sterile deionised water to remove the remaining medium. 2 μL of the sample was dropped and air-dried on the aluminium-coated slide. Nikon Ti Eclipse was used for fluorescence microscopy. Fiji was used for image analysis.

Scanning Electron Microscopy (SEM)

To prepare sterile coverslips, clean coverslips were dipped in 70% ethanol overnight, air-dried then exposed to UV light for 30 min. For coating, clean and sterile coverslips were dipped into 0.01% poly-L-lysine solution for 1-2 hours at room temperature, then washed twice with sterile water. The coated coverslips were allowed to dry in a 60°C drying hood overnight before use and were stored at 4°C in a sterile container for further use. For sample preparation, purified mini-SimCell culture was diluted into an appropriate concentration to avoid overlap between cells. The coated coverslips were dipped in the culture and incubated at 37°C for 30 minutes. For primary fixation, the coverslips were dipped into the 2.5% glutaraldehyde solution and fixed at room temperature for 1 hour. After primary fixation, the samples can be stored at 4°C for up to 1 week or proceeded to secondary fixation. For secondary fixation, the fixed samples were first rinsed with pH=7.2 0. 1M PIPES buffer for three times, 5 min each time. A sufficient volume of 1% OsO_4 in 0.1 M PIPES buffer was used to cover the samples. The samples were incubated at 4 °C for 1 hour and then rinsed with ddH_2O three times, 5 min each time. Subsequent to secondary fixation, the samples were dehydrated using graded EtOH concentrations of 50%, 70%, 90%, and 95%, one time for 5 min each and finally three times using 100% EtOH for 10 min. For the drying step, the EtOH was removed and 0.5 mL hexamethyldisilazane (HMDS) was quickly added to each sample. After 3 min incubation, the HMDS was removed, and the samples were left for air dry overnight or at least 3 hours to allow the HMDS fumes to evaporate. This step was performed in the fume hood. Once dried, the samples were attached to carbon adhesive tape on an SEM stub, sputter-coated with gold/palladium, and then viewed using Zeiss Sigma 300 SEM. Fiji was used for image analysis.

Mini-SimCell size distribution and concentration characterisation

The size distribution and concentration of Mini-SimCells were characterised by nanoparticle tracking analysis (NTA) using ZetaView (Particle Metrix, Inning am Ammersee, Germany). Prior to the measurement, Silica 100 nm microspheres (Polysciences Inc., Philadelphia, United States) were used for the quality check. Purified mini-SimCells were diluted in 1xPBS to an

optimal concentration. The same batch of 1xPBS solution was used as the control group. 1mL of each sample was loaded onto the sample chamber using a syringe to avoid introducing bubbles. Background 1×PBS was used for flushing away the previous sample before loading a new sample. In the sample chamber, mini-SimCells were illuminated by a laser beam, the scattered light was captured, and the movement of each particle over time was tracked by a live camera. Based on Brownian motion, the diffusion coefficient of the particles was calculated, and then the size of each particle was determined using the Stokes-Einstein equation. Mini-SimCell concentrations were calculated by the number of particles in a known volume compared to the control group. Each sample was measured from 11 positions in 2 cycles. Parameter set as: sensitivity = 80, frame = 30, shutter speed = 100. For T6SS Mini-SimCells, a 500 nm filter was added to observe the fluorescent particles selectively.

H_2O_2 generation assay

The concentration of hydrogen peroxide is measured using a fluorimetric hydrogen peroxide assay kit (Sigma-Aldrich MAK165). 10mL of 800 µM catechol in M9 minimal medium and blank M9 minimal medium is prepared and continuously incubated at 37 °C, 180rpm. At 1, 3, 7, 11 and 21 day, 50 µL of each sample is collected and mixed with 50 µL of the Master Mix in each well of 96-well plate (Greiner, black). All samples were run in triplicates. The plate was incubated at room temperature for 30 min in the dark. The fluorescence intensity is measured at $\lambda ex = 540$/ $\lambda em = 590$ nm using a fluorescence plate reader (Tecan Spark). Gain = 60. All readings were corrected by subtracting the background. The hydrogen peroxide concentration for the samples is calculated based on the standard curve.

Disc diffusion method for antimicrobial activity characterisation of catechol

This method is adapted from Kirby-Bauer disc diffusion method for antibiotic susceptibility testing. An overnight bacterial suspension was prepared and normalised to OD_{600} =2.0. Sterile L-shaped spreaders were used to evenly spread the bacterial suspension over the surface of fresh LB agar plates. Allowing the inoculum to dry for a short period, small paper discs impregnated with 50 µL of 0/ 5 mM/ 10 mM/ 0.1 M catechol were placed on the surface of the agar plates. Sterile forceps were used for placing the discs. Triplicates were applied for each plate with even spacing between discs. All agar plates were incubated at 37°C for 24 hours before capturing the images. Inhibition zones appeared as clear, circular areas around each disc. Fiji was used for image analysis and measurement of inhibition zones.

Assessing permeability and extracellular antimicrobial activity of cell-produced catechol

Overnight cultures were 1:100 re-inoculated into fresh LB medium with antibiotics for selection, grew until stationary phase and centrifuged to collect all cells. Cell pellets were washed twice with 1×PBS to remove traces of culture medium and antibiotics, then resuspended with fresh minimal medium (M9 medium) with 800μM Aspirin. All cultures were normalised to a volume of 50 mL and OD_{600} of 2.0. After overnight incubation at 37°C, 180 rpm, the supernatants were collected, filtered for sterilisation, and mixed in different ratios with concentrated fresh LB medium to normalise the nutrition level. The final supernatant-LB mediums contained 20%, 50%, 80% of the supernatant respectively and 1xLB. Then these media were used as the growth medium for prey cells (*E coli* DH10B). Overnight cultures of prey cells were 1:1000 re-inoculated into 200 μL of different growth media with corresponding antibiotics in a flat 96-well plate. The plate was sealed with Breathe-Easy sealing membrane and incubated with a Synergy 2 microplate reader (BioTek) at 37 °C, 1000 rpm. OD_{600} was measured every 15 min for 18 hours.

Supplementary Tables

Table S1. Comparison of advantages and challenges between various antimicrobials.

	Approach	Biosafety	Stability	Resistance risk	Manufacture	Refs
Traditional antimicrobials	Antibiotics	√	√	High	√	(11–13)
Non-traditional antimicrobials	Antimicrobial peptides (AMPs)	70% of AMPs are predicted to have hemolytic activity	Instable, with short half-lives	Medium	√	(14–16)
	Bacteriophage	√	√	High	Difficult	(17–20)
	Monoclonal antibodies	√	√	Medium	Complex and Expensive	(21–23)
	Nanoparticles (NPs)	Potential toxic effects in human health and environment	Appropriate stabilizer required	Low	Can be difficult	(24–26)
	Gas therapy	Can be toxic	Uncontrollable, reliable delivery system to-be-developed	Low	√	(27–29)
	Live biotherapeutic products (LBPs)	Biosafety and biocontainment concerns	√	Medium to low	√	(30–33)

Table S2. Strains and plasmids used in this study.

Strain	Description	Source
Escherichia coli DH5a		Lab collection
E. coli DH10B		Lab collection
E. coli BL21(DE3)		Lab collection
E. coli BL21(DE3) ΔminD		Lab collection
Pseudomonas putida UWC1		Lab collection
E. coli ST131		NCTC 13441
BL21: Ag	*E. coli* BL21(DE3): pDSG287_sfGFP	This study
BL21: Nb	*E. coli* BL21(DE3): pDSG289 + pL1_WH002_EF	This study
BL21: Null	*E. coli* BL21(DE3): pDSG291 + pL1_WH002_EF	This study
BL21: constitutive T6SS	*E. coli* BL21(DE3): pT6S_NP	This study
BL21: induced T6SS	*E. coli* BL21(DE3): pT6S_Tet	This study
BL21: Nb+T6SS+	*E. coli* BL21(DE3): pDSG289 + pT6S_NP	This study
BL21: Nb+T6SS-	*E. coli* BL21(DE3): pDSG289 + pT6S_N3-NP	This study
BL21: Null T6SS+	*E. coli* BL21(DE3): pDSG291 + pT6S_NP	This study
BL21: Null T6SS-	*E. coli* BL21(DE3): pDSG291 + pT6S_N3-NP	This study
BL21: Nb+NahG	*E. coli* BL21(DE3): pNb_NahG	This study
BL21: Nb39_mRFP	*E. coli* BL21(DE3): pNb39_NahG	This study
SimCell	*E. coli* BL21(DE3): pRH12x	This study
Nb-SimCell	*E. coli* BL21(DE3): pRH12x + pDSG289	This study
Null-SimCell	*E. coli* BL21(DE3): pRH12x + pDSG291	This study
SimCell: constitutive T6SS	*E. coli* BL21(DE3): pRH12x + pT6S_NP	This study
SimCell: induced T6SS	*E. coli* BL21(DE3): pRH12x + pT6S_Tet	This study
SimCell: Nb+T6SS+	*E. coli* BL21(DE3): pRH12x + pDSG289 + pT6S_NP	This study
SimCell: Nb+T6SS-	*E. coli* BL21(DE3): pRH12x + pDSG289 + pT6S_N3-NP	This study
SimCell: Null T6SS+	*E. coli* BL21(DE3): pRH12x + pDSG291 + pT6S_NP	This study
SimCell: Null T6SS-	*E. coli* BL21(DE3): pRH12x + pDSG291 + pT6S_N3-NP	This study
SimCell: Nb+NahG	*E. coli* BL21(DE3): pRH12x + pNb_NahG	This study
Mini-SimCell (Mini-SimCell Null)	*E. coli* BL21(DE3) ΔminD	This study
Mini-SimCell: constitutive T6SS	*E. coli* BL21(DE3) ΔminD: pT6S_NP	This study
Mini-SimCell: induced T6SS	*E. coli* BL21(DE3) ΔminD: pT6S_Tet	This study
Mini-SimCell: Nb+T6SS+	*E. coli* BL21(DE3) ΔminD: pDSG289 + pT6S_NP	This study
Mini-SimCell: Nb+T6SS-	*E. coli* BL21(DE3) ΔminD: pDSG289 + pT6S_N3-NP	This study
Mini-SimCell: Null T6SS+	*E. coli* BL21(DE3) ΔminD: pDSG291 + pT6S_NP	This study
Mini-SimCell: Null T6SS-	*E. coli* BL21(DE3) ΔminD: pDSG291 + pT6S_N3-NP	This study
Mini-SimCell: Nb+NahG	*E. coli* BL21(DE3) ΔminD: pNb_NahG	This study

Mini-SimCell Nb39-T6SS	*E. coli* BL21(DE3) ΔminD: pTet_Nb39 + pT6S_NP	This study
Mini-SimCell Nb39-nahG	*E. coli* BL21(DE3) ΔminD: pNb39_NahG	This study

**Plasmid**	**Description**	**Source**
pDSG287	Plasmid with surface-displayed EPEA	Addgene #115606 (34)
pDSG287_sfGFP	Plasmid with surface-displayed EPEA and sfGFP	Dataset S1, this study
pDSG289	Plasmid with surface-displayed anti-EPEA	Addgene #115608 (34)
pDSG291	Plasmid with surface-displayed Null	Addgene #115605 (34)
pL1_WH002_EF	Plasmid with constitutive high expression of mRFP	Lab collection
pT6S_NP	Plasmid with T6SS gene cluster from *Aeromonas dhakensis* under native constitutive promoter	Gift from Tao Dong, Southern University of Science and Technology, China (35)
pT6S_Tet	Plasmid with T6SS gene cluster from *Aeromonas dhakensis* under tetracycline-inducible promoter	(35)
pT6S_N3-NP	Plasmid with inactive T6SS gene cluster from *Aeromonas dhakensis* under native constitutive promoter	(35)
pT6S_N3-Tet	Plasmid with inactive T6SS gene cluster from *Aeromonas dhakensis* under tetracycline-inducible promoter	(35)
pRH12x	Plasmid with I-CeuI endonuclease	Dataset S2, lab collection(1)
pNb_NahG	Plasmid with constitutive high expression of anti-EPEA, salicylate hydroxylase NahG and mRFP	Dataset S3, this study
pTet_Nb39	Plasmid with surface-displayed Nb39	Dataset S4, this study
pNb39_NahG	Plasmid with constitutive high expression of Nb39, salicylate hydroxylase NahG and mRFP	Dataset S5, this study

Table S3. Primers used in this study.

**Primer**	**Sequence 5' to 3'**
EPEA FWD	ATTTCACACAGGAACCTGACGTCTAAGAAACC
EPEA REV	CATCAAAAGATACATCAGAGCTTTTACGAG
EPEA-sfGFP FWD	CAGGAAAGAAACCATTATTATCATGACATTAAC
EPEA-sfGFP REV	CATCAAACATCAAGGGAAAACTGTCCATATGC
AmpR-mid-F	GATCCAGTTCGATGTAACCCACTCG
intimin-mid-R	CGGCAACAGTTTCACCAGTTTTCAAC
intiminN-test-F	GTCGAATGGTCAAGTTGTCGACCAG
Ag/Nb-seq-R	GCTGTTCACCATGAACAGATCGACAATG

nahG-Test-R	TCAACCAGATGTTCGTCGATCCC
mRFP-test-F	AGCTTCCACCGAACGTATG
vgrG-Seq-R	GTGCTCGTCATGCTTGATAT
vipA-Seq-F	GCCCAAAGAACGTATCAATATC
SP9	ATGTCAAACTTTATACTTAAACCGG
SP10	TGAGCTGCTTCTAAATTAGGAAA
SPGG_2 FWD	CAGATAAAAAAAATCCTTAGCTTTCGCT
SPGG_3 REV	TAACCGTATTACCGCCTTTGAGTG

Supplementary Figures

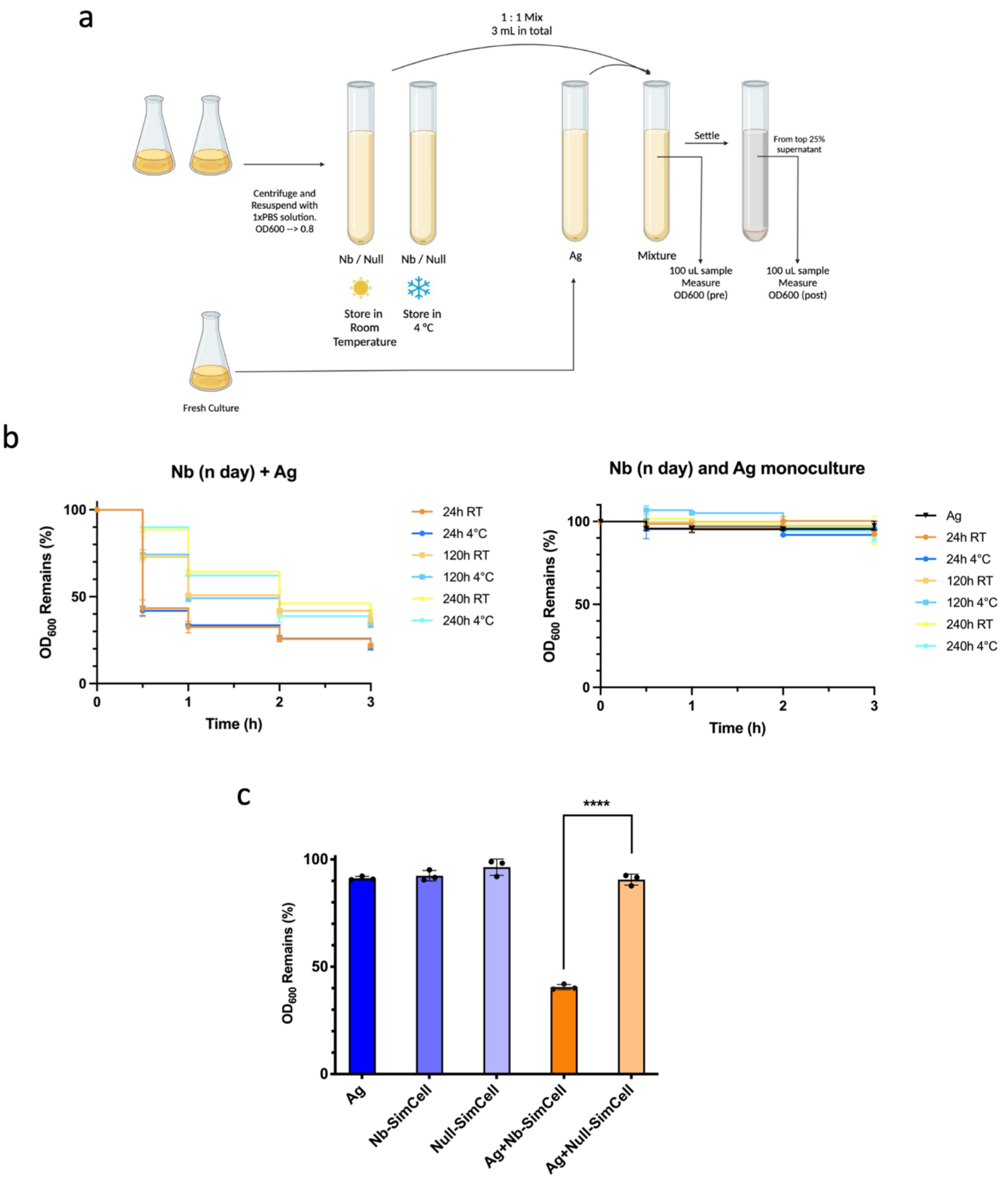

Figure S1. Stability of surface-displayed nanobody. (a) Macroscopic aggregation assay allows quantitative measurement of binding strength via optical density (OD_{600}). Adapted from previous work(34). **(b)** Macroscopic aggregation tests confirm that cells stored at room temperature (RT) or 4°C maintained the nanobody activity after 10 days. **(c)** SimCell Ag+Nb paired mixture shows significant aggregation-caused settling compared to unmixed and non-adhesion (Ag+Null) conditions after 3 hrs. For **(b)** and **(c)**, all cultures were normalised to the same initial OD_{600} and mixed in 1:1 v/v ratio. n = 3 replicates, Error bars, ±1 SD, ****$p < 0.0001$ according to a 2-tailed paired t-test.

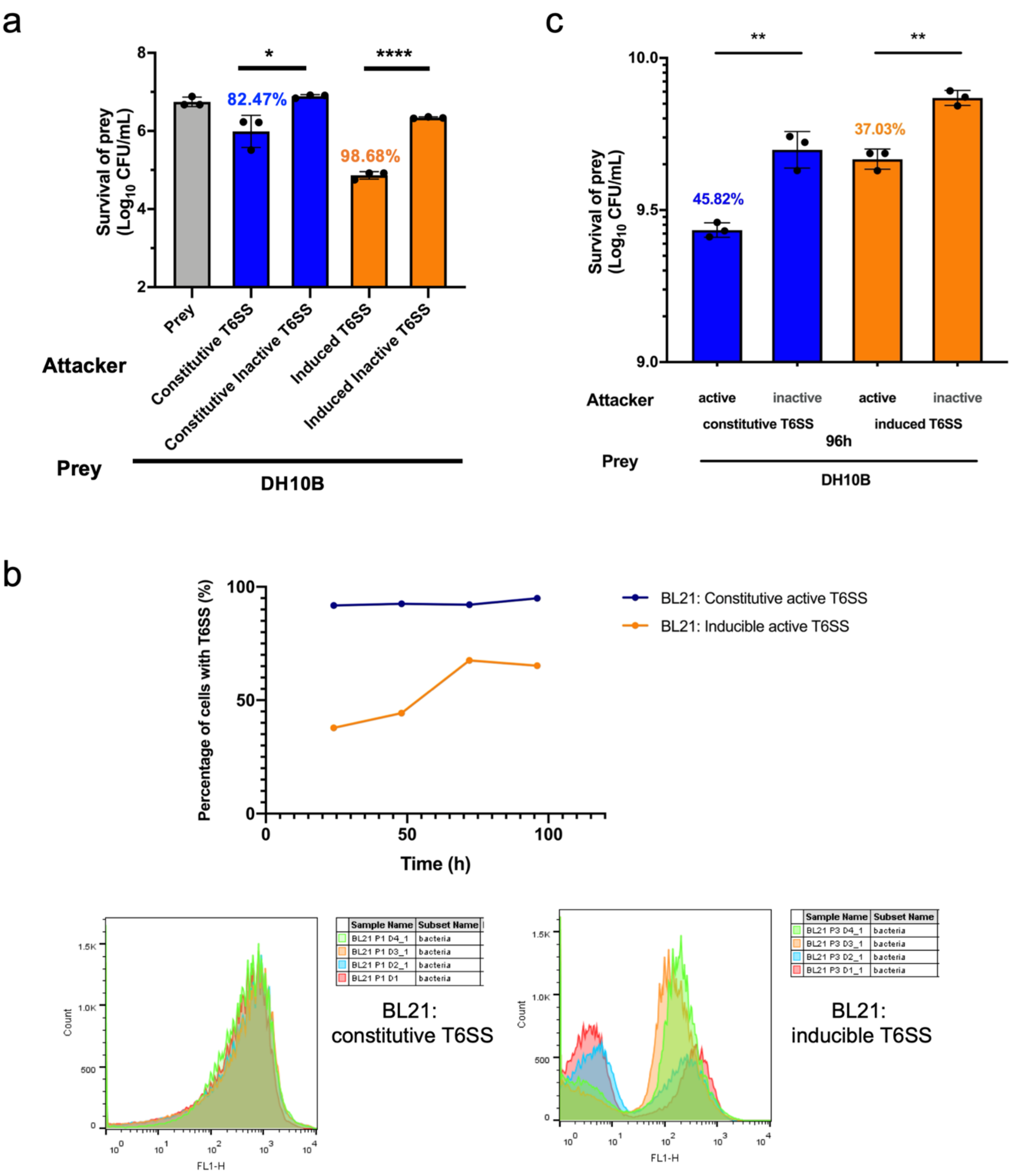

Figure S2. Function and stability of heterologous T6SS in *E. coli* BL21(DE3) cells. (a) Significant killing in solid (contact-promoted) condition indicates both constitutive and induced T6SS activity. **(b)** Flow cytometry results showing the GFP-labelling cells (T6SS-expressed cells) among the population and the change after continuous incubation. >97% constitutive expressed T6SS are stable for at least 96h. **(c)** After 96h continuous incubation, both constitutive and induced expressed-T6SS BL21 parental cells maintain certain activity. n = 3 replicates, Error bars, ±1 SD, $**p < 0.01$ according to a 2-tailed paired t-test. Killing efficiency was calculated as 1-survival cells/ total prey cells.

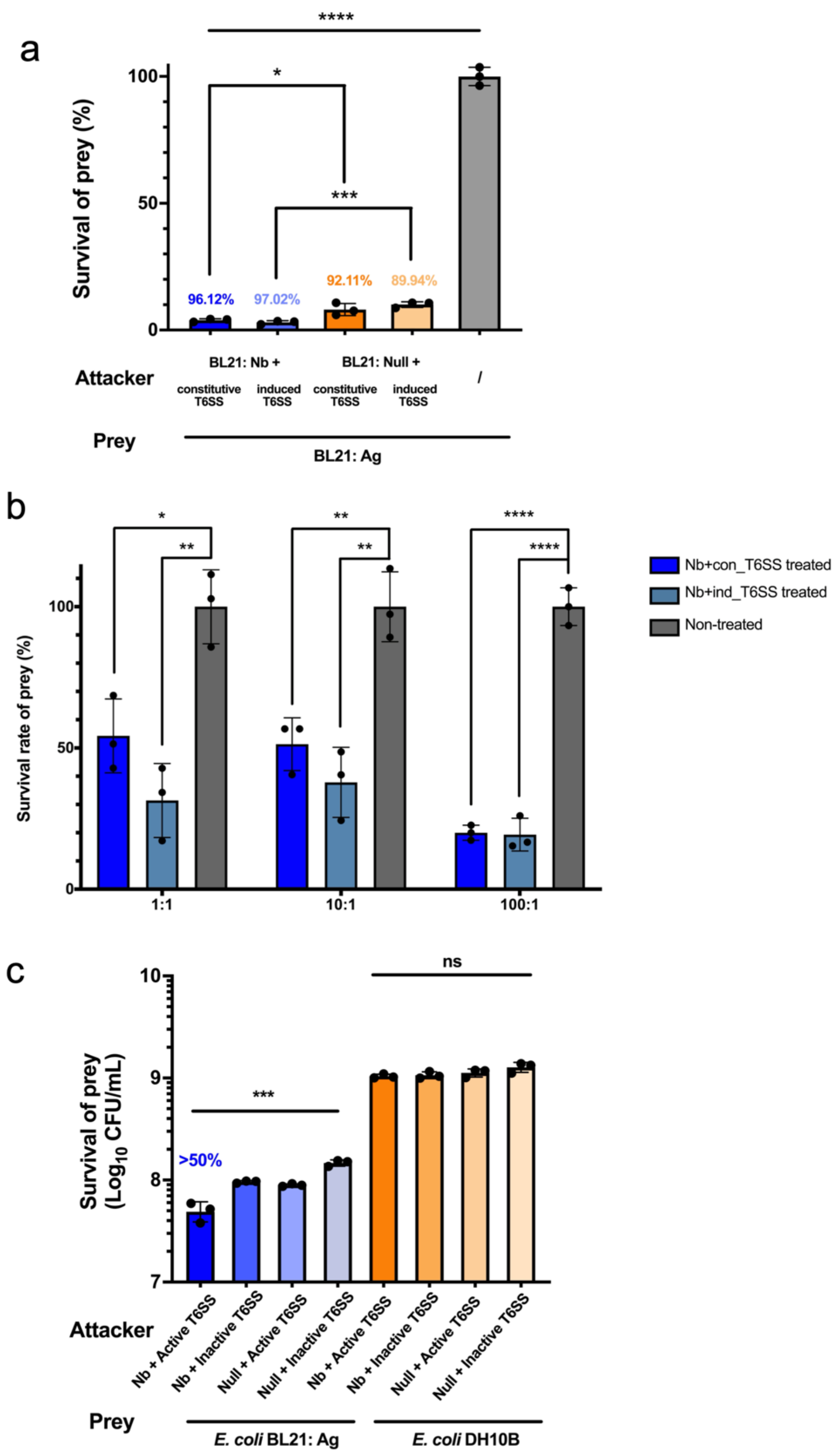

Figure S3. Nanobody-antigen binding enhanced T6SS in killing efficiency and enabled T6SS with killing specificity. (a) Nanobody-antigen interaction further improves T6SS-mediated killing in solid condition. **(b)** Significant inhibition efficiency was observed with a lower to attacker: prey = 1:1 n:n ratio of Nb-T6SS BL21 parental cells as attacker cells. The efficiency was improved by increasing n:n ratio. **(c)** Nb-Ag recognition enables a specific T6SS-killing effect. For **(a)** to **(c)**, n = 3 replicates, Error bars, ±1 SD, $*p < 0.1$, $**p < 0.01$, $***p < 0.001$, $****p < 0.0001$ according to a 2-tailed paired t-test. Survival rate was calculated as survival cells/ total prey cells. Killing efficiency was calculated as 1-survival rate.

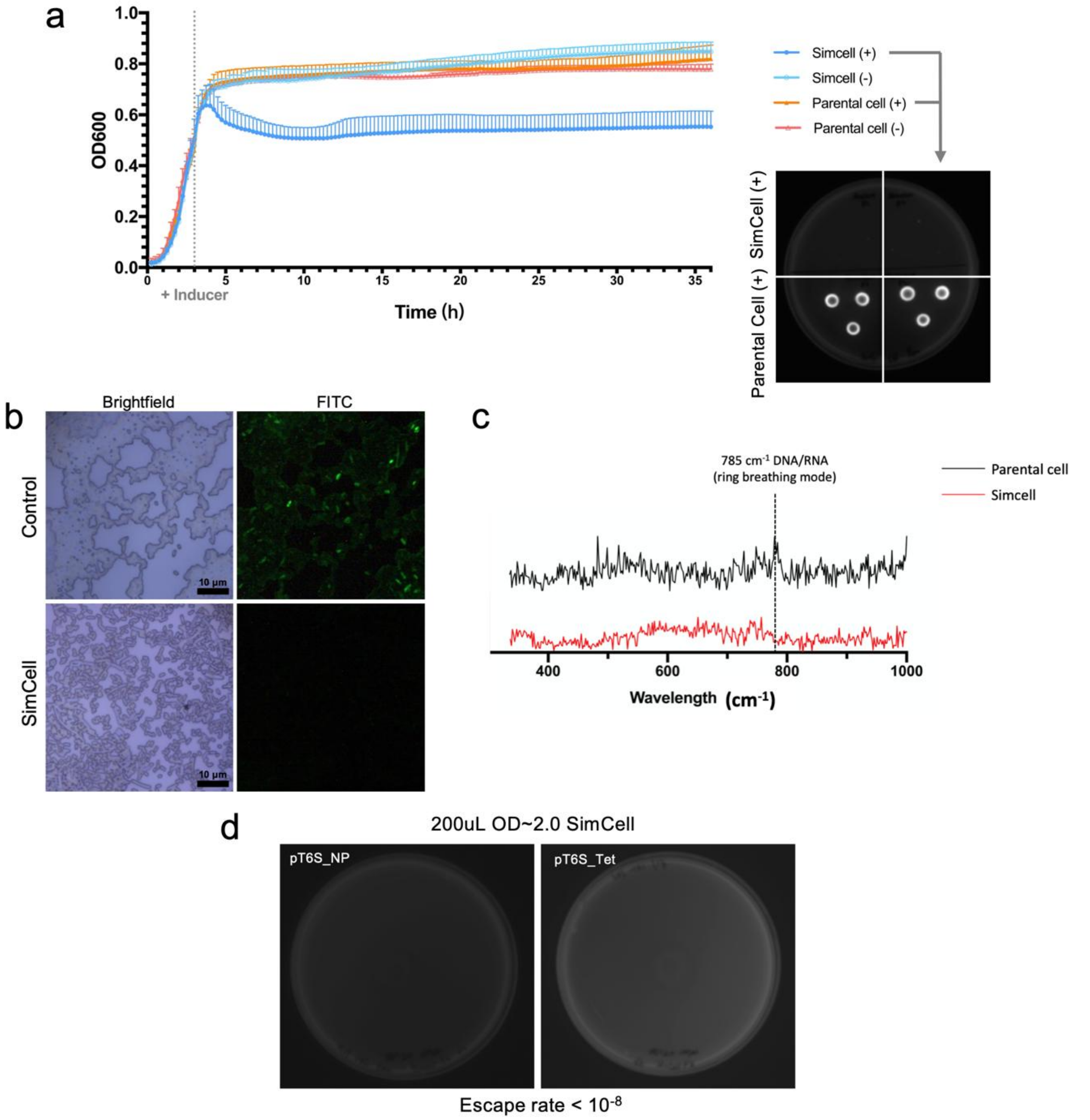

Figure S4. Characteriasation of T6SS-assembled SimCells. (a) SimCell conversion and purity check. Growth assays confirmed the successful SimCell conversion from the engineered parent cell. OD_{600} was continuously measured every 15min to monitor the growth curve over 35 hours. The grey dot vertical line indicates the time point (3h after initial incubation) of inducer addition (1 µM crystal violet). Growth arrest was observed shortly after inducer addition for induced SimCells. n = 3 replicates, Error bars, +1 SD. Agar plate culturing confirmed a high efficiency of SimCell conversion. All cultures were plated on LB agar plates and incubated for 24 hours at 37 °C. **(b)** SYTO 9 stain assay confirms successful SimCell conversion at single cell level. Green fluorescence can be observed in control cells due to double-strand chromosomes being stained while neglectable in SimCells. Scale bar, 10 µm. **(c)** Raman analysis confirms DNA reduction in SimCells. DNA/RNA peak in 785 cm^{-1}. **(d)** No colony is observed from plating 200 uL of OD_{600} = 2.0 SimCell culture in LB agar plates, confirming the escape rate of lower than 10^{-8}. All cultures were plated on LB agar plates and incubated for 24 hours at 37 °C.

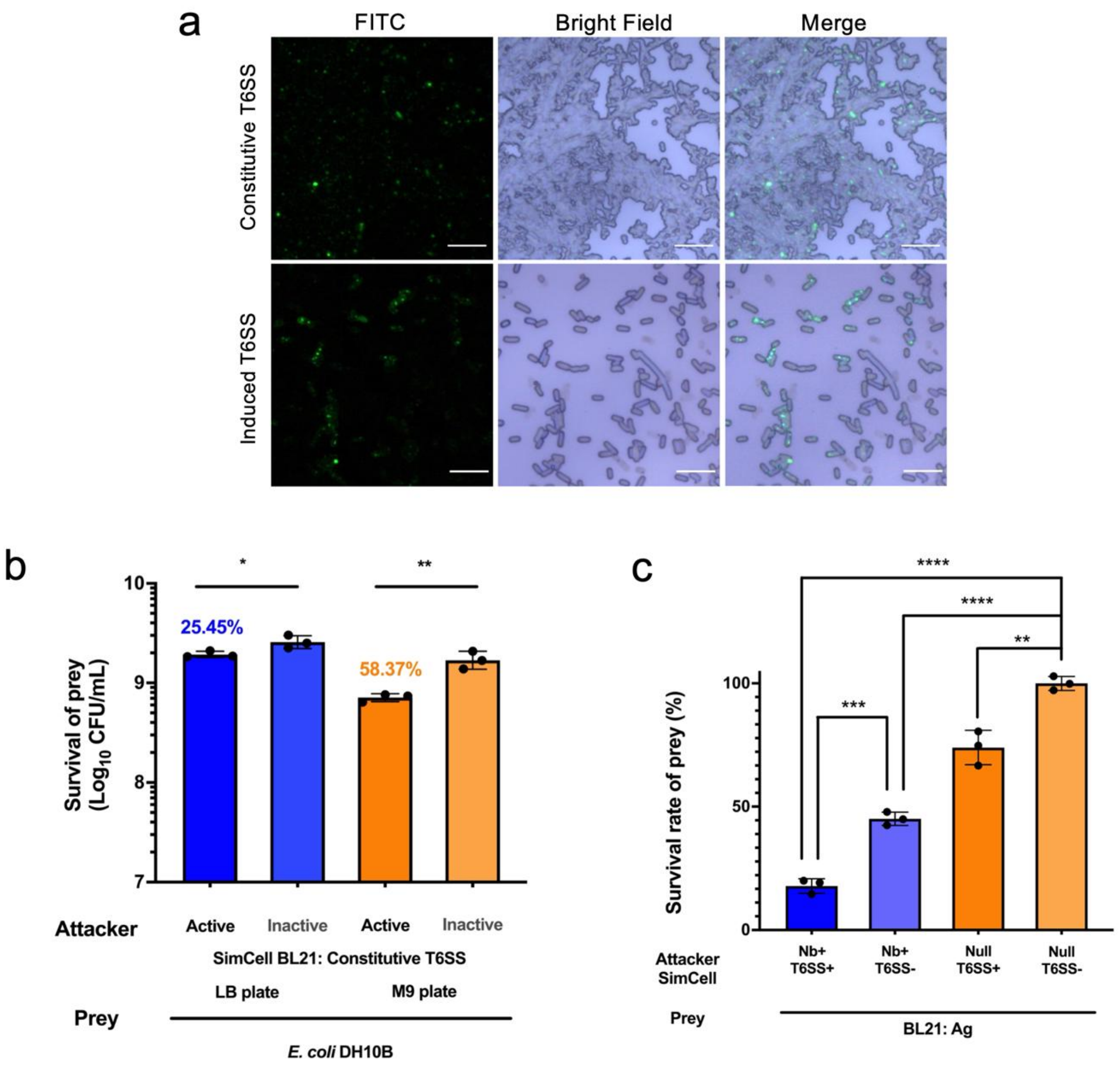

Figure S5. T6SS-mediated killing in reprogrammed SimCells. (a) Fluorescence microscopic analysis confirms the assembly of T6SS in *E. coli* strain BL21(DE3)-derived SimCells. Scale bar, 10 µm. **(b)** Function tests of T6SS-assembled SimCells in solid (contact-promoted) condition. Incubation time = 3h. Attacker : prey v/v ratio = 10:1. **(c)** Nanobody-antigen binding enhances T6SS-mediated killing in SimCells in liquid conditions. Incubation time = 6h. Attacker : prey v/v ratio = 10:1. For **(b)** and **(c)**, n = 3 replicates, Error bars, ±1 SD, *p < 0.1, **p < 0.01, ***p < 0.001, ****p < 0.0001 according to a 2-tailed paired t-test. Survival rate was calculated as survival cells/ total prey cells.

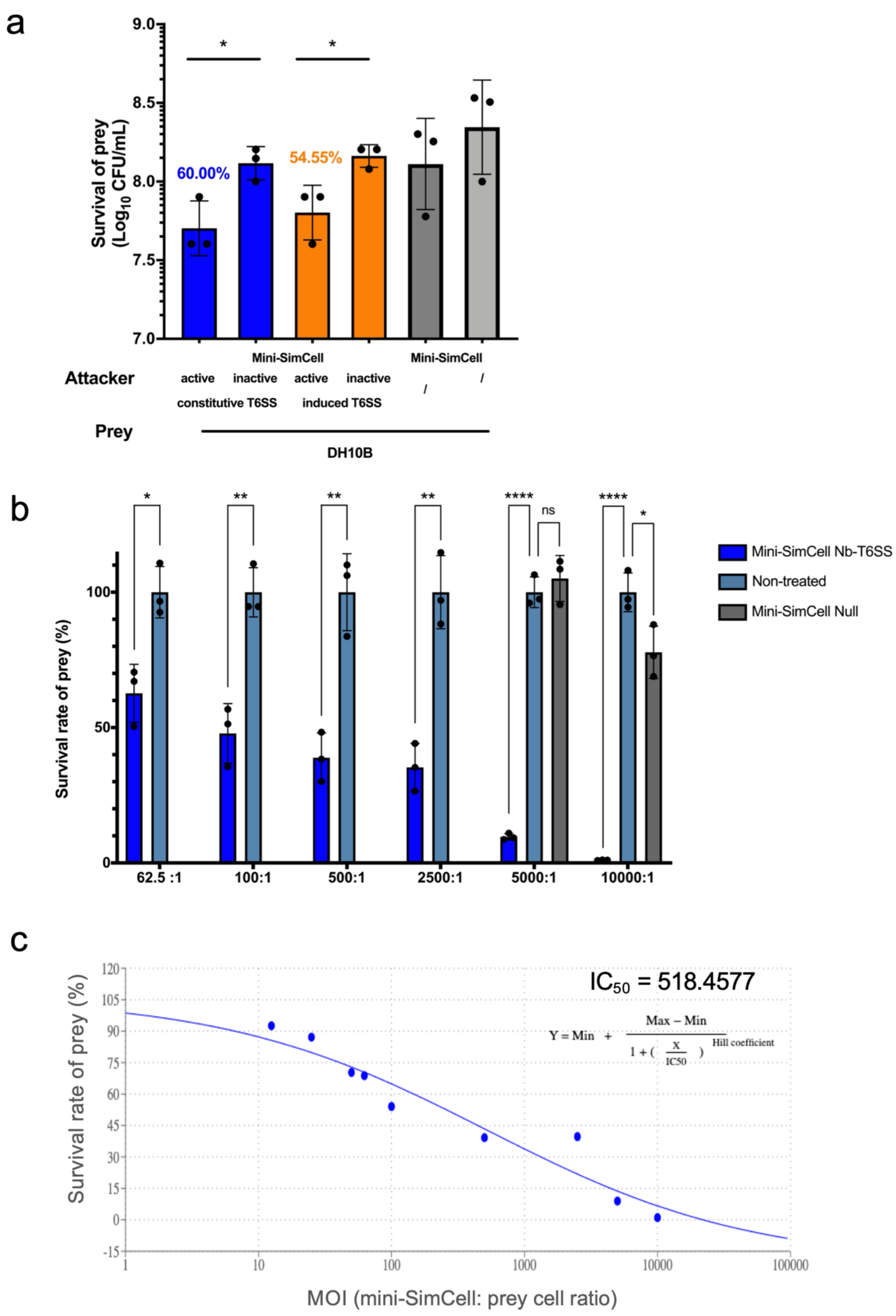

a
9.0
8.5
8.0
7.5
7.0
*
*
60.00%
54.55%
Survival of prey (Log10 CFU/mL)
Attacker
Mini-SimCell
active
inactive
active
inactive
constitutive T6SS
induced T6SS
Mini-SimCell
/
/
Prey
DH10B
b
Survival rate of prey (%)
100
50
0
62.5 :1
100:1
500:1
2500:1
5000:1
10000:1
ns
Mini-SimCell Nb-T6SS
Non-treated
Mini-SimCell Null
c
IC50 = 518.4577
Y = Min + (Max − Min) / (1 + (X / IC50)^Hill coefficient)
Survival rate of prey (%)
MOI (mini-SimCell: prey cell ratio)

Figure S6. Functional characterisation of T6SS-assembled mini-SimCell. (a) Function tests of T6SS-assembled mini-Simcells in solid (contact-promoted) condition. Incubation time = 3h. Attacker : prey v/v ratio = 10:1. **(b)** Limit of killing (LOK) of Nb-T6SS mini-SimCells. Attackers (mini-SimCell Nb-T6SS) were mixed in liquid condition with prey cells in n/n ratio of Attacker : prey = 62.5/100/500/2,500/5,000/10,000:1, total Attaker cells were $1*10^{10}$, the maximum tolerated dose reported in the previous human trial. Incubation time =6h. Significant inhibition efficiency was observed in all groups. The efficiency was improved by increasing n:n ratio. **(c)** The inhibitory concentration curve of T6SS-assembled mini-SimCells. A half-maximal inhibitory concentration (IC50) is around 518.5 T6SS-mini-SimCells per prey cell. For **(a)** to **(c)**, n = 3 replicates, Error bars, ±1 SD, *p < 0.1, **p < 0.01, ****p < 0.0001 according to a 2-tailed paired t-test. Survival rate was calculated as survival cells/ total prey cells. Killing efficiency was calculated as 1-survival rate.

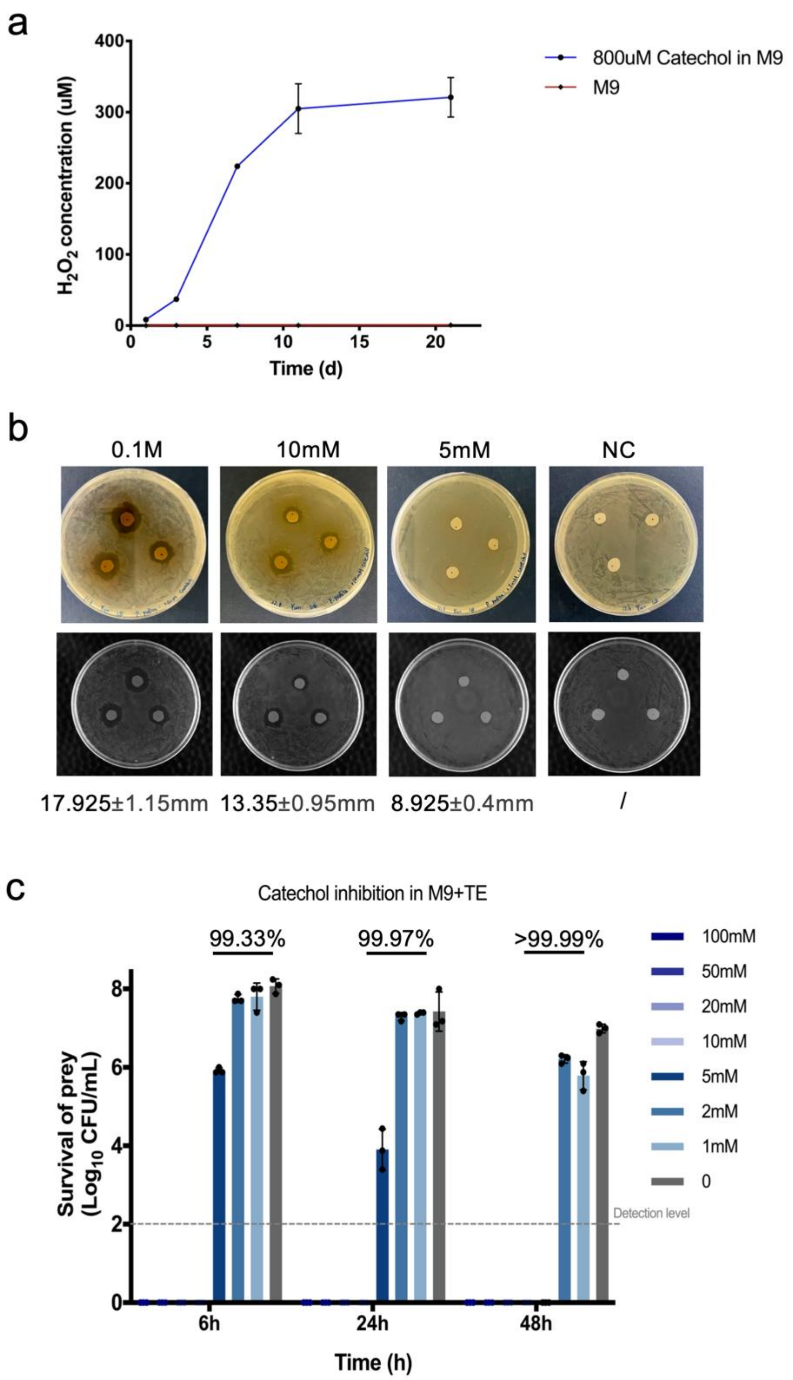

Figure S7. The antibacterial activity of catechol. (a) H_2O_2 production over time from 800 μM catechol in 1x PBS at 37°C. Samples were taken at Day 1, 3, 7, 11 and 21. n = 3 replicates, Error bars, ±1 SD. **(b)** The antimicrobial activity of catechol was determined by the disc diffusion method. Values above the figures are the concentration of catechol, and values below the figures are diameters of inhibition zones, DIZ (mm) ±1 SD, n = 3 replicates. **(c)** Inhibitory efficiency of different concentrations of catechol. 100/50/20/10/5/2/1mM of catechol was added into M9+TE (trace elements) medium and inoculated with 10^8 prey cells (*E coli* DH10B). After 6h, 24h and 48h incubation at 37°C, the survival of prey cells was determined by plating assay. n = 3 replicates, Error bars, ±1 SD. Killing efficiency was calculated as 1-(survival cells/ total prey cells).

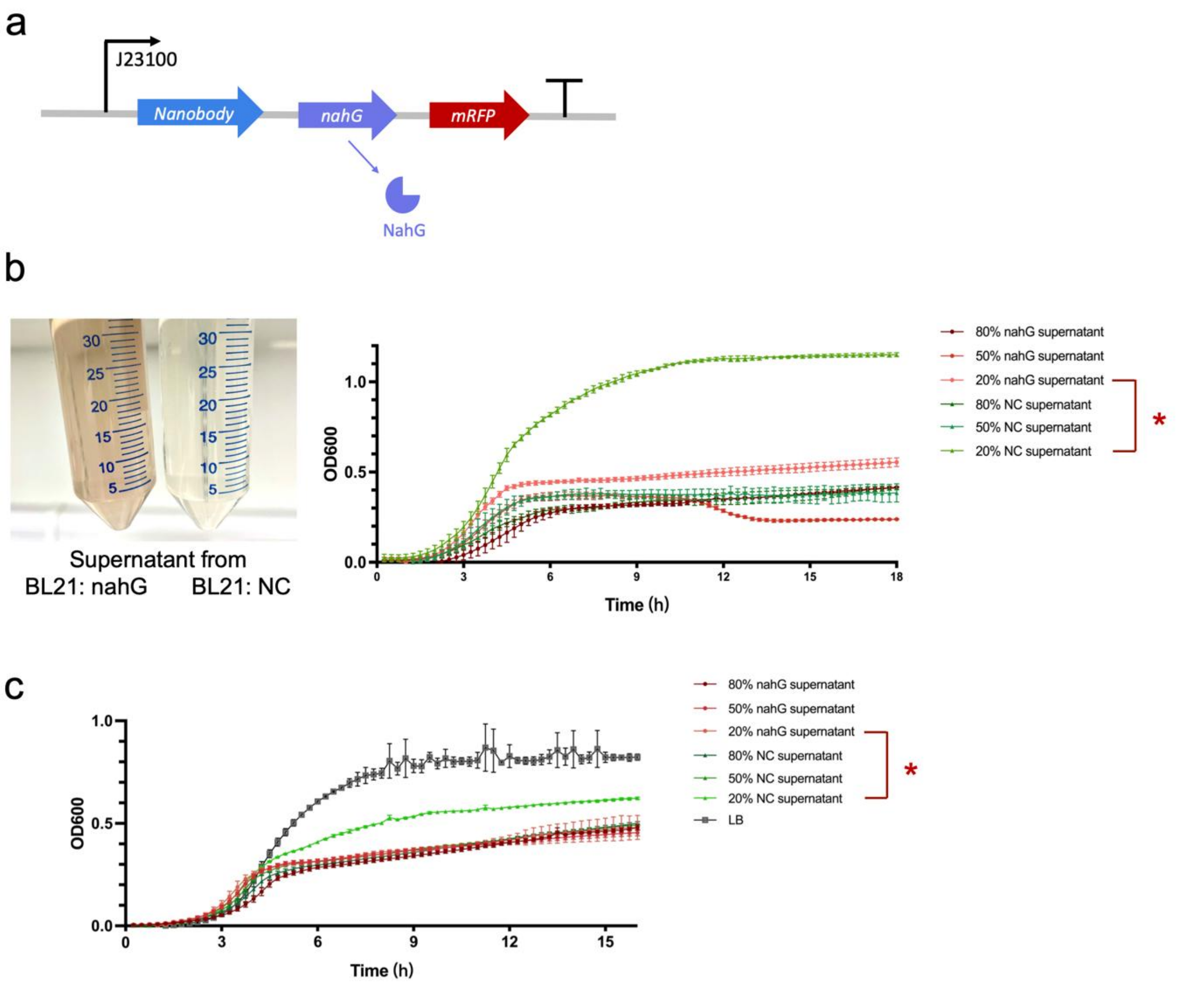

Figure S8. The antimicrobial activity of cell-produced catechol. (a) Engineered genetic circuit design for catechol generation. Nanobody and NahG enzyme are co-expressed under a strong constitutive promoter J23100. mRFP is co-expressed as an indicator for expression. **(b)** Supernatant from 37°C overnight 800µM Aspirin + BL21: nahG/NC culture. The brown colour indicates the generation of catechol and its oxidation products. The supernatants were collected, filtered for sterilisation and mixed in different ratios with concentrated LB to normalise the nutrition level. Then the supernatant-LBs were used as the growth medium for prey cells (E coli DH10B). 20% nahG supernatant showed a significant inhibition effect compared to the NC group. n = 3 replicates, Error bars, ±1 SD. **(c)** Supernatant from 37°C overnight 800µM Aspirin + BL21-derived SimCell: nahG/NC culture. Similar results with the parental cells. n = 3 replicates, Error bars, ±1 SD.

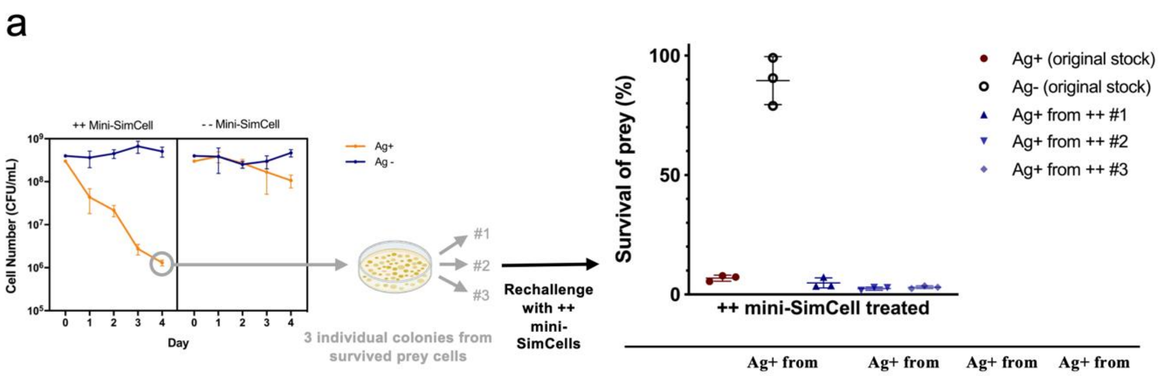

	Ag+ from original stock)	Ag+ from ++ #1	Ag+ from ++ #2	Ag+ from ++ #3
Killing efficiency	93.14 ±1.27%	95.14 ±2.11%	97.52 ±0.66%	96.96 ±0.56%

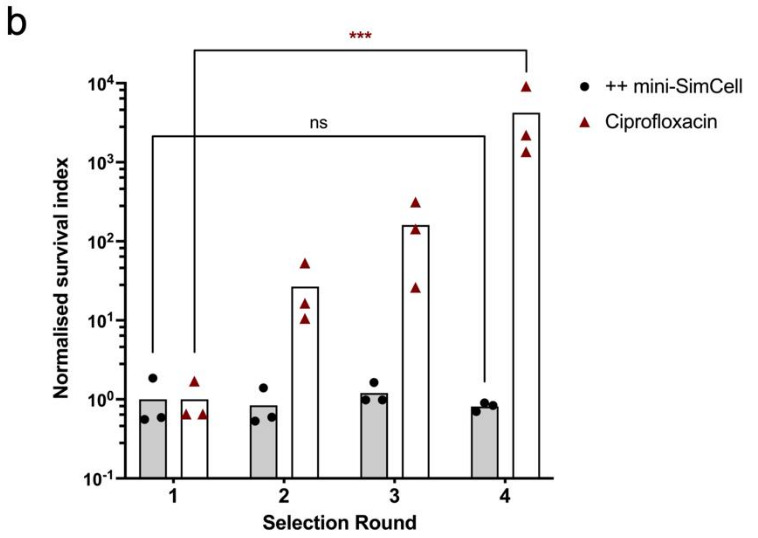

Figure S9. Slow emergence of resistance to reprogrammed mini-SimCells. (a) No significant resistance observed among survived Ag+ population from mixed community after 4 doses of ++ mini-SimCells treatment. MOI = 1,000:1. All cultures were incubated at 37 °C for 24 hrs. 800 µM Aspirin was added after 6h. Killing efficiency was calculated as 1-(survival cells/ total prey cells). **(b)** Normalised survival index (NSI) of 48 hrs ++ mini-SimCells (MOI of 1,000:1) and ciprofloxacin (0.00195µg/mL) treatment against Ag+ prey cells after indicated number of rounds of passage. NSI was calculated as the ratio of survived prey cells (CFU/mL) after selection round X to survived prey cells (CFU/mL) after the first selection round. Each round of selection was initiated with a fresh population of ++ mini-SimCells. ***$p < 0.001$ according to a 2-tailed unpaired t-test on log10-transformed NSI values. For **(a)** and **(b)**, n = 3 replicates, Error bars, ±1 SD.

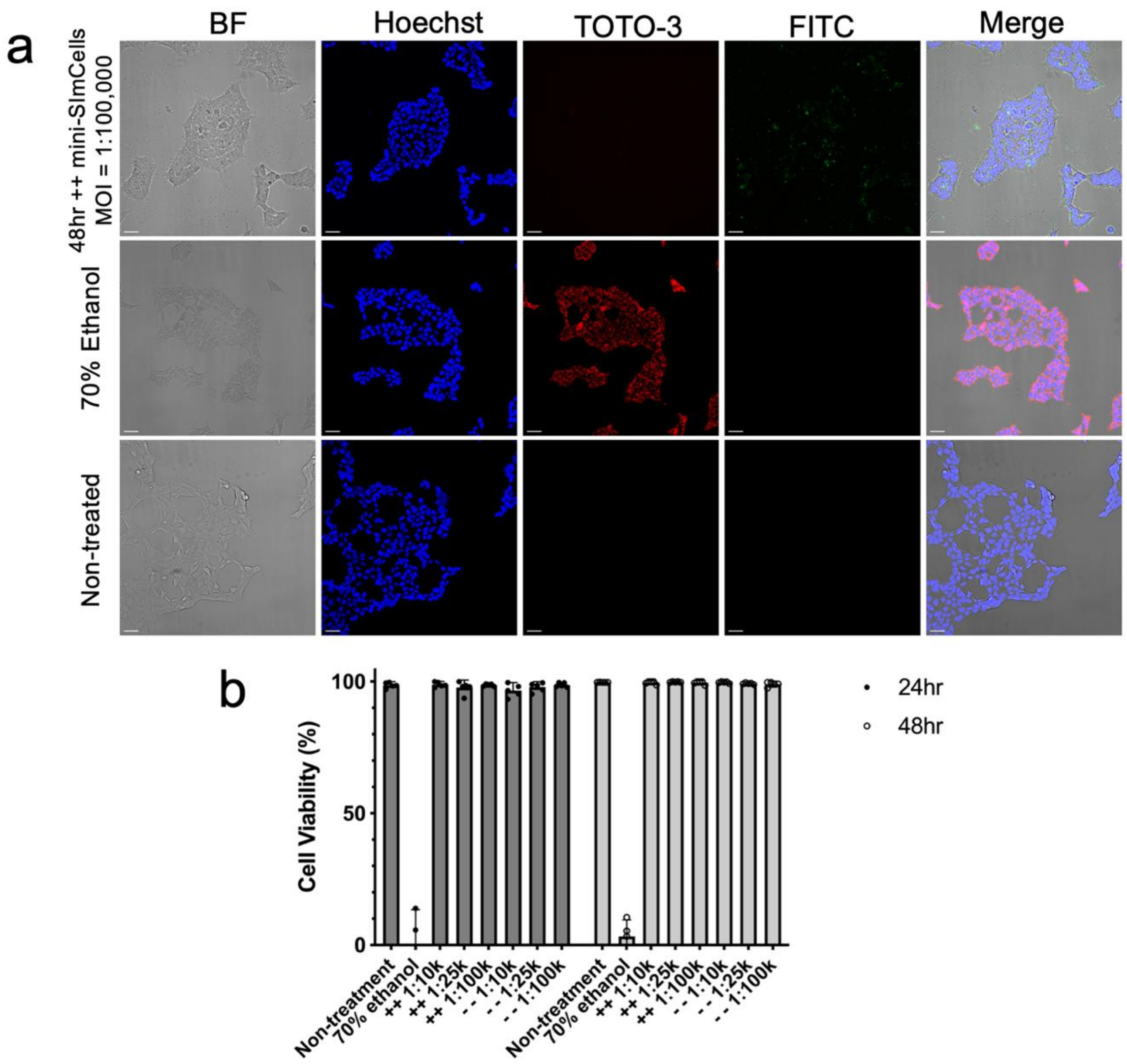

Figure S10. *In vitro* cytotoxicity assessment of reprogrammed mini-SimCells to mammalian cells. (a) Live/dead cell staining fluorescence microscopy of HEK293 cells following 48-hour treatment with reprogrammed ++ mini-SimCells at MOI 1:100,000 (top), 70% ethanol-treated positive control (middle), and untreated negative control (bottom). Nuclei stained with Hoechst 33258 (blue), dead cells stained with TOTO-3 (red), Nb+T6SS mini-SimCells expressing sfGFP (green). Scale bar, 50 μm. **(b)** Quantification of HEK293 cell viability following treatment with reprogrammed (++) or non-engineered (- -) mini-SimCells at MOI 1:10,000, 1:25,000, and 1:100,000 for 24 and 48 hours. n = 5 replicates, Error bars, ±1 SD.

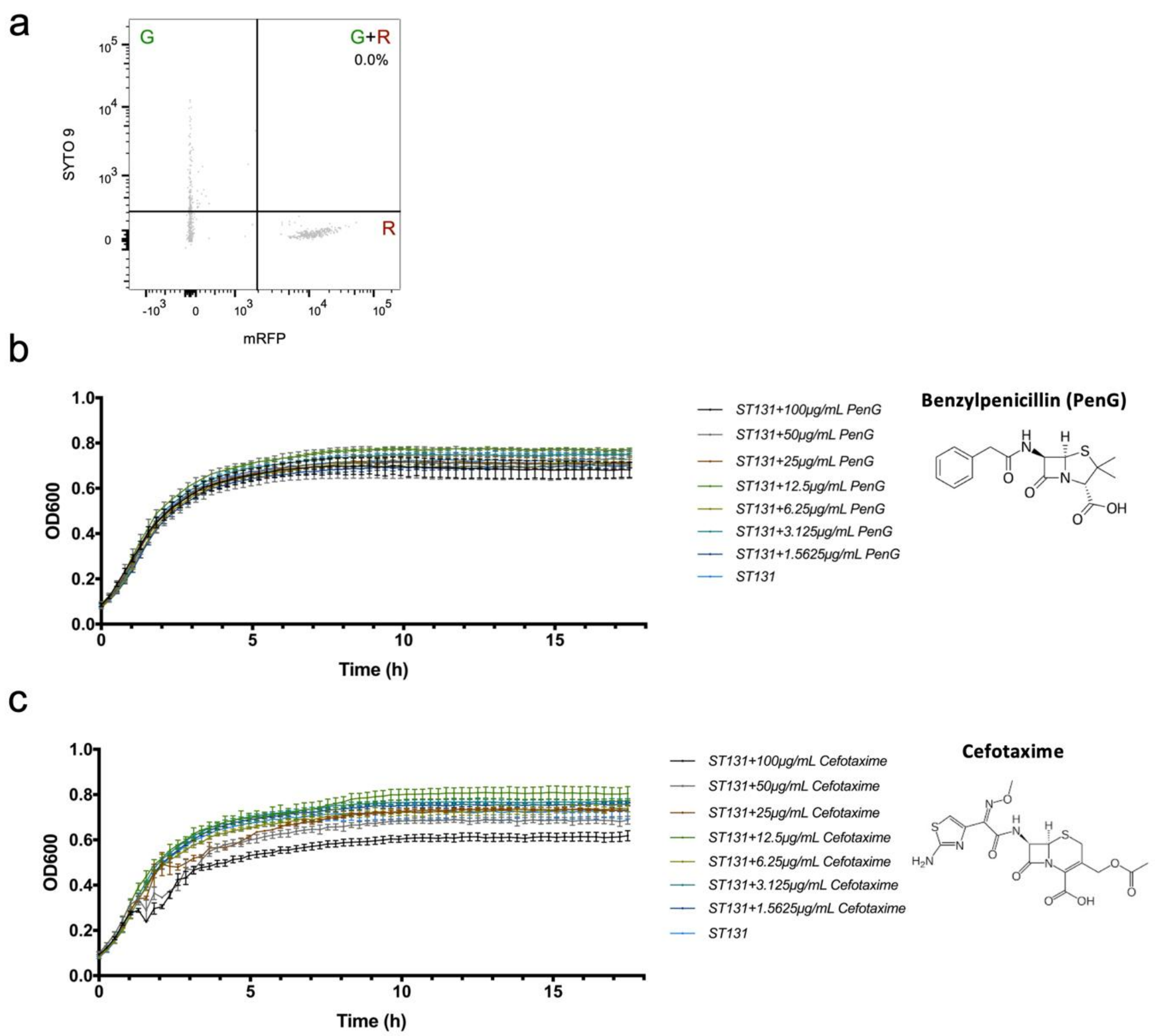

Figure S11. (a) Negative control result for binding quantification based on double-color flow cytometry assay. BL21: Ag and BL21: Nb39_mRFP mixture was set as unbinding negative control. Events of doublets formation (G+R) refers to the captured BL21: Ag cells by cells displaying Nb39 nanobody. BL21: Ag : BL21: Nb39_mRFP cell = 1:1. **(b)-(c) *E. coli* ST131 strain exhibits multi-drug resistance. (b)** *E. coli* ST131 growth remains unaffected by benzylpenicillin (PenG) with concentration up to 100 μg/mL. **(c)** *E. coli* ST131 growth shows no significant inhibition by cefotaxime with concentration up to 100 μg/mL. N = 3 replicates, Error bars, ±1 SD.

References

1. C. Fan, *et al.*, Chromosome-free bacterial cells are safe and programmable platforms for synthetic biology. *Proc Natl Acad Sci U S A* **117**, 6752–6761 (2020).
2. S. L. Liu, A. Hessel, K. E. Sanderson, Genomic mapping with I-Ceu I, an intron-encoded endonuclease specific for genes for ribosomal RNA, in Salmonella spp., Escherichia coli, and other bacteria. *Proc Natl Acad Sci U S A* **90**, 6874 (1993).
3. DEPARTMENT OF HEALTH AND HUMAN SERVICES National Institutes of Health, NIH GUIDELINES FOR RESEARCH INVOLVING RECOMBINANT OR SYNTHETIC NUCLEIC ACID MOLECULES (NIH GUIDELINES). (2024).
4. S. Razaviamri, K. Wang, B. Liu, B. P. Lee, Catechol-Based Antimicrobial Polymers. *Molecules 2021, Vol. 26, Page 559* **26**, 559 (2021).
5. I. Kocaçalıskocaçalıs¸kan, I. Talan, I. Terzi, "Antimicrobial Activity of Catechol and Pyrogallol as Allelochemicals" (2006).
6. N. Schweigert, A. J. B. Zehnder, R. I. L. Eggen, Chemical properties of catechols and their molecular modes of toxic action in cells, from microorganisms to mammals. *Environ Microbiol* **3**, 81–91 (2001).
7. J. Yang, M. A. Cohen Stuart, M. Kamperman, Jack of all trades: Versatile catechol crosslinking mechanisms. *Chem Soc Rev* [Preprint] (2014).
8. D. E. Fullenkamp, D. G. Barrett, D. R. Miller, J. W. Kurutz, P. B. Messersmith, pH-dependent cross-linking of catechols through oxidation via Fe3+ and potential implications for mussel adhesion. *RSC Adv* **4**, 25127–25134 (2014).
9. H. Meng, Y. Li, M. Faust, S. Konst, B. P. Lee, Hydrogen peroxide generation and biocompatibility of hydrogel-bound mussel adhesive moiety. *Acta Biomater* **17**, 160–169 (2015).
10. H. Meng, *et al.*, Biomimetic recyclable microgels for on-demand generation of hydrogen peroxide and antipathogenic application. *Acta Biomater* **83** (2019).
11. N. S. Devi, *et al.*, Overview of antimicrobial resistance and mechanisms: The relative status of the past and current. *The Microbe* **3**, 100083 (2024).
12. M. Miethke, *et al.*, Towards the sustainable discovery and development of new antibiotics. *Nature Reviews Chemistry 2021 5:10* **5**, 726–749 (2021).
13. L. L. Silver, Challenges of antibacterial discovery. *Clin Microbiol Rev* **24**, 71–109 (2011).
14. C. Bucataru, C. Ciobanasu, Antimicrobial peptides: Opportunities and challenges in overcoming resistance. *Microbiol Res* **286**, 127822 (2024).
15. C. D. Fjell, J. A. Hiss, R. E. W. Hancock, G. Schneider, Designing antimicrobial peptides: form follows function. *Nature Reviews Drug Discovery 2012 11:1* **11**, 37–51 (2011).
16. F. Plisson, O. Ramírez-Sánchez, C. Martínez-Hernández, Machine learning-guided discovery and design of non-hemolytic peptides. *Scientific Reports 2020 10:1* **10**, 1–19 (2020).
17. A. Bruttin, H. Brüssow, Human volunteers receiving Escherichia coli phage T4 orally: A safety test of phage therapy. *Antimicrob Agents Chemother* **49** (2005).
18. A. Wright, C. H. Hawkins, E. E. Änggård, D. R. Harper, A controlled clinical trial of a therapeutic bacteriophage preparation in chronic otitis due to antibiotic-resistant Pseudomonas aeruginosa; a preliminary report of efficacy. *Clinical Otolaryngology* **34**, 349–357 (2009).
19. D. Holger, *et al.*, Clinical Pharmacology of Bacteriophage Therapy: A Focus on Multidrug-Resistant Pseudomonas aeruginosa Infections. *Antibiotics 2021, Vol. 10, Page 556* **10**, 556 (2021).
20. S. J. Labrie, J. E. Samson, S. Moineau, Bacteriophage resistance mechanisms. *Nature Reviews Microbiology 2010 8:5* **8**, 317–327 (2010).

21. A. DiGiandomenico, B. R. Sellman, Antibacterial monoclonal antibodies: the next generation? *Curr Opin Microbiol* **27**, 78–85 (2015).
22. I. Lowy, *et al.*, Treatment with Monoclonal Antibodies against Clostridium difficile Toxins . *New England Journal of Medicine* **362**, 197–205 (2010).
23. M. Troisi, *et al.*, A new dawn for monoclonal antibodies against antimicrobial resistant bacteria. *Front Microbiol* **13** (2022).
24. R. Li, *et al.*, Antimicrobial nanoparticles: current landscape and future challenges. *RSC Pharmaceutics* **1**, 388–402 (2024).
25. J. R. Morones, *et al.*, The bactericidal effect of silver nanoparticles. *Nanotechnology* **16**, 2346 (2005).
26. M. Rai, A. Yadav, A. Gade, Silver nanoparticles as a new generation of antimicrobials. *Biotechnol Adv* **27**, 76–83 (2009).
27. T. Y. Wang, X. Y. Zhu, F. G. Wu, Antibacterial gas therapy: Strategies, advances, and prospects. *Bioact Mater* **23**, 129–155 (2023).
28. L. K. Wareham, R. K. Poole, M. Tinajero-Trejo, CO-releasing metal carbonyl compounds as antimicrobial agents in the post-antibiotic era. *Journal of Biological Chemistry* **290**, 18999–19007 (2015).
29. D. O. Schairer, J. S. Chouake, J. D. Nosanchuk, A. J. Friedman, The potential of nitric oxide releasing therapies as antimicrobial agents. *Virulence* **3**, 271–279 (2012).
30. U.S. Department of Health and Human Services Food and Drug Administration, Early Clinical Trials with Live Biotherapeutic Products: Chemistry, Manufacturing, and Control Information; Guidance for Industry. (2016).
31. D. T. Riglar, P. A. Silver, Engineering bacteria for diagnostic and therapeutic applications. *Nature Reviews Microbiology 2018 16:4* **16**, 214–225 (2018).
32. A. Cubillos-Ruiz, *et al.*, Engineering living therapeutics with synthetic biology. *Nature Reviews Drug Discovery 2021 20:12* **20**, 941–960 (2021).
33. A. Rouanet, *et al.*, Live Biotherapeutic Products, A Road Map for Safety Assessment. *Front Med (Lausanne)* **7** (2020).
34. D. S. Glass, I. H. Riedel-Kruse, A Synthetic Bacterial Cell-Cell Adhesion Toolbox for Programming Multicellular Morphologies and Patterns. *Cell* **174**, 649-658.e16 (2018).
35. Y. Cui, *et al.*, Heterologous Assembly of the Type VI Secretion System Empowers Laboratory Escherichia coli with Antimicrobial and Cell Penetration Capabilities. *Appl Environ Microbiol* **88** (2022).
