# Supplementary material for: Reprogrammed SimCells for antimicrobial therapy
Source: Proc Natl Acad Sci U S A. 2026 Mar 17;123(12):e2517118123. doi: 10.1073/pnas.2517118123 (PMC13012131; doi:10.1073/pnas.2517118123)
Supplement: Supplementary file 2 — Dataset S01 (PDF) [file pnas.2517118123.sd01.pdf]

pDSG287-sfGFP (7161 bp)

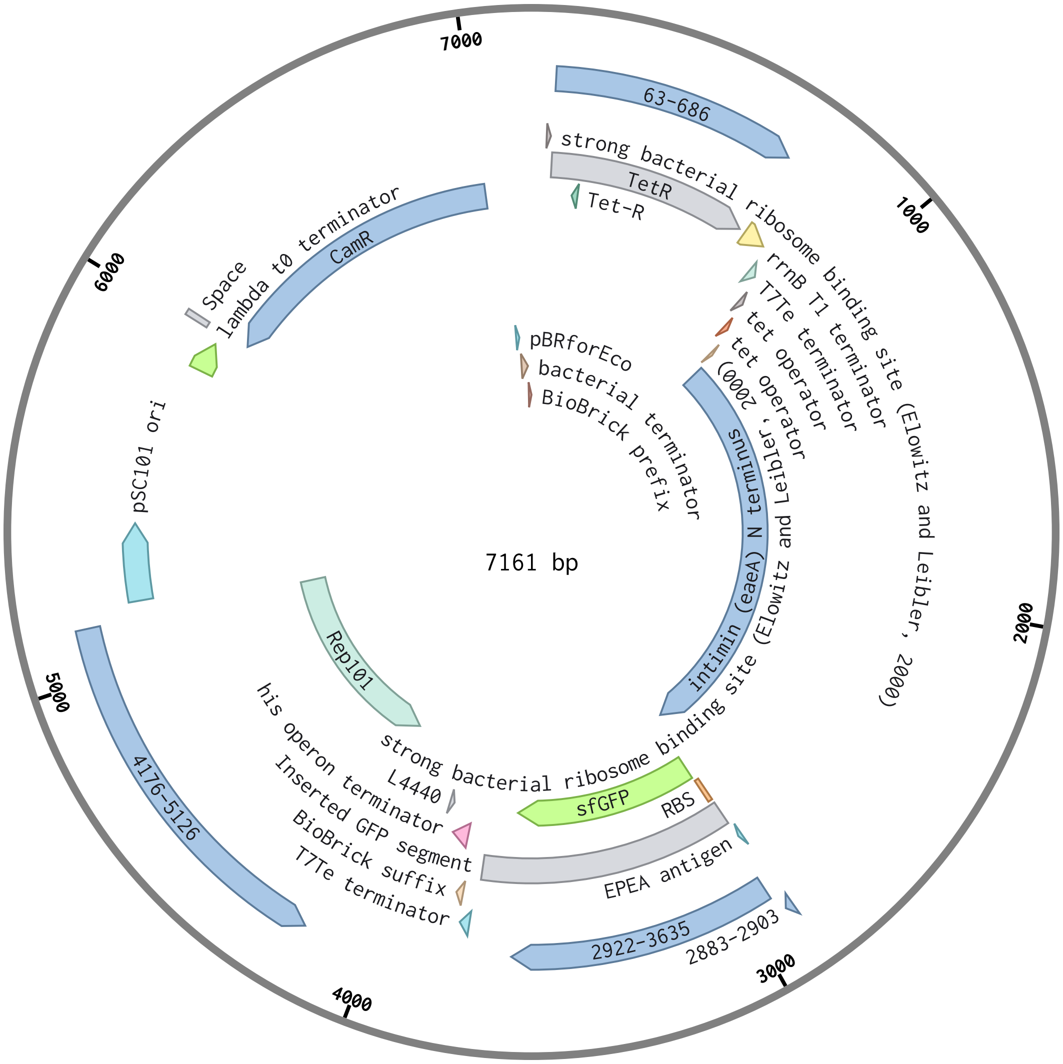

pDSG287-sfGFP (7161 bp)

```
tggtgcaaaacctttgcggtatggcatgatagcgcctactagagaaagaggagaaatactagatgtccagattagataaaagtaaagtgattaacagcgcattagag
accacgttttggaaacgccataccgtactatcgcggatgatctctttctcctctttatgatctacaggtctaatctattttcatttcactaattgtcgcgtaatctc
```

M S R L D K S K V I N S A L E

63-686

str...0) TetR

10 20 30 40 50 60 70 80 90 100

```
ctgcttaatgaggtcggaatcgaaggtttaacaacccgtaaactcgcccagaagctaggtgtagagcagcctacattgtattggcatgtaaaaaataagcgggcttt
gacgaattactccagccttagcttccaaattgttgggcatttgagcgggtcttcgatccacatctcgtcggatgtaacataaccgtacattttttattcgcccgaaa
```

L L N E V G I E G L T T R K L A Q K L G V E Q P T L Y W H V K N K R A L

63-686

Tet-R

TetR

110 120 130 140 150 160 170 180 190 200 210

```
gctcgacgccttagccattgagatgttagataggcaccatactcacttttgccctttagaaggggaaagctggcaagattttttacgtaataacgctaaaagtttta
cgagctgcggaatcggtaactctacaatctatccgtggtatgagtgaaaacgggaaatcttcccctttcgaccgttctaaaaaatgcattattgcgattttcaaaat
```

L D A L A I E M L D R H H T H F C P L E G E S W Q D F L R N N A K S F

63-686

TetR

220 230 240 250 260 270 280 290 300 310 320

```
gatgtgctttactaagtcatcgcgatggagcaaaagtacatttaggtacacggcctacagaaaaacagtatgaaactctcgaaaatcaattagccttttatgccaa
ctacacgaaatgattcagtagcgctacctcgttttcatgtaaatccatgtgccggatgtctttttgtcatactttgagagcttttagttaatcggaaaatacggtt
```

R C A L L S H R D G A K V H L G T R P T E K Q Y E T L E N Q L A F L C Q

63-686

TetR

330 340 350 360 370 380 390 400 410 420

```
caaggttttcactagagaatgcattatatgcactcagcgctgtggggcattttactttaggttgcgtattggaagatcaagagcatcaagtcgctaaagaagaaag
gttccaaaagtgatctcttacgtaatatacgtgagtcgcgacaccccgtaaaatgaaatccaacgcataaccttctagttctcgtagttcagcgatttcttctttc
```

Q G F S L E N A L Y A L S A V G H F T L G C V L E D Q E H Q V A K E E R

63-686

TetR

430 440 450 460 470 480 490 500 510 520 530

```
ggaaacacctactactgatagtatgccgccattattacgacaagctatcgaattatttgatcaccaaggtgcagagccagccttcttattcggccttgaattgatca
cctttgtggatgatgactatcatacggcggtaataatgctgttcgatagcttaataaactagtggttccacgtctcggtcggaagaataagccggaacttaactagt
```

E T P T T D S M P P L L R Q A I E L F D H Q G A E P A F L F G L E L I

63-686

TetR

540 550 560 570 580 590 600 610 620 630 640

tatgcggattagaaaaacaacttaaatgtgaaagtgggtcctaataatactagagccaggcatcaaataaaacgaaaggctcagtcgaaagactgggcctttcgttt
atacgcctaatctttttgttgaatttacactttcacccaggattattatgatctcggtccgtagtttattttgctttccgagtcagctttctgacccggaaagcaaa

I C G L E K Q L K C E S G S *

63-686

TetR

rrnB T1 terminator

650 660 670 680 690 700 710 720 730 740

tatctgttgtttgtcggtgaacgctctctactagagtcacactggctcaccttcgggtgggcctttctgcgtttatatactagagtccctatcagtgatagagattg
atagacaacaaacagccacttgcgagagatgatctcagtgtgaccgagtggaagcccacccggaaagacgcaaatatatgatctcagggatagtcactatctctaac

rrnB T1 terminator

T7Te terminator

tet operator

750 760 770 780 790 800 810 820 830 840 850

acatccctatcagtgatagagatactgagcactactagagaaagaggagaaatactagatgattactcatggttgttatacccggacccggcacaagcataagctaa
tgtagggatagtcactatctctatgactcgtgatgatctctttctcctctttatgatctactaatgagtaccaacaatatgggcctgggccgtgttcgtattcgatt

tet operator

str...0)

intimin (eaeA) N terminus

860 870 880 890 900 910 920 930 940 950 960

aaaaaacattgattatgcttagtgctggtttaggattgtttttttatgttaatcagaactcatttgcaaatggtgaaaattattttaaattgggttcggattcaaaa
ttttttgtaactaatacgaatcacgaccaaatcctaacaaaaaaatacaattagtcttgagtaaacgtttaccacttttaataaaatttaacccaagcctaagtttt

intimin (eaeA) N terminus

970 980 990 1,000 1,010 1,020 1,030 1,040 1,050 1,060 1,070

ctgttaactcatgatagctatcagaatcgcctttttttatacgttgaaaactggtgaaactgttgccgatctttctaaatcgcaagatattaatttatcgacgatttg
gacaattgagtactatcgatagtcttagcggaaaaaaatatgcaacttttgaccactttgacaacggctagaaagatttagcgttctataattaaatagctgctaaac

intimin (eaeA) N terminus

1,080 1,090 1,100 1,110 1,120 1,130 1,140 1,150 1,160 1,170

gtcgttgaataagcatttatacagttctgaaagcgaaatgatgaaggccgcgcctggtcagcagatcattttgccactcaaaaaacttccctttgaatacagtgcac
cagcaacttattcgtaaatatgtcaagactttcgctttactacttccggcgcggaccagtcgtctagtaaaacggtgagttttttgaagggaaacttatgtcacgtg

intimin (eaeA) N terminus

1,180 1,190 1,200 1,210 1,220 1,230 1,240 1,250 1,260 1,270 1,280

taccacttttaggttcggcacctcttgttgctgcgggtggtgttgctggtcacacgaataaactgactaaaatgtccccggacgtgaccaaaagcaacatgaccgat
atggtgaaaatccaagccgtggagaacaacgacgcccaccacaacgaccagtgtgcttatttgactgattttacaggggcctgcactggttttcgttgtactggcta

intimin (eaeA) N terminus

1,290 1,300 1,310 1,320 1,330 1,340 1,350 1,360 1,370 1,380 1,390

gacaaggcattaaattatgcggcacaacaggcggcgagtctcggtagccagcttcagtcgcgatctctgaacggcgattacgcgaaagataccgctcttggtatcgc
ctgttccgtaatttaatacgccgtgttgtccgccgctcagagccatcggtcgaagtcagcgctagagacttgccgctaatgcgctttctatggcgagaaccatagcg

intimin (eaeA) N terminus

1,400 1,410 1,420 1,430 1,440 1,450 1,460 1,470 1,480 1,490

tggtaaccaggcttcgtcacagttgcaggcctggttacaacattatggaacggcagaggttaatctgcaaagtggtaataactttgacggtagttcactggacttct
accattggtccgaagcagtgtcaacgtccggaccaatgttgtaataccttgccgtctccaattagacgtttcaccattattgaaactgccatcaagtgacctgaaga

intimin (eaeA) N terminus

1,500 1,510 1,520 1,530 1,540 1,550 1,560 1,570 1,580 1,590 1,600

tattaccgttctatgattccgaaaaaatgctggcatttggtcaggtcggagcgcgttacattgactcccgctttacggcaaatttaggtgcgggtcagcgtttttttc
ataatggcaagatactaaggctttttttacgaccgtaaaccagtccagcctcgcgcaatgtaactgagggcgaaatgccgtttaaatccacgcccagtcgcaaaaaag

intimin (eaeA) N terminus

1,610 1,620 1,630 1,640 1,650 1,660 1,670 1,680 1,690 1,700 1,710

cttcctgcaaacatgttgggctataacgtcttcattgatcaggatttttctggtgataatacccgtttaggtattggtggcgaatactggcgagactatttcaaaag
gaaggacgtttgtacaacccgatattgcagaagtaactagtcctaaaaagaccactattatgggcaaatccataaccaccgcttatgaccgctctgataaagttttc

intimin (eaeA) N terminus

1,720 1,730 1,740 1,750 1,760 1,770 1,780 1,790 1,800 1,810

tagcgttaacggctatttccgcatgagcggctggcatgagtcatacaataagaaagactatgatgagcgcccagcaaatggcttcgatatccgttttaatggctatc
atcgcaattgccgataaaggcgtactcgccgaccgtactcagtatgttattctttctgatactactcgcgggtcgtttaccgaagctataggcaaaattaccgatag

intimin (eaeA) N terminus

1,820 1,830 1,840 1,850 1,860 1,870 1,880 1,890 1,900 1,910 1,920

taccgtcatatccggcattaggcgccaagctgatatatgagcagtattatggtgataatgttgctttgtttaattctgataagctgcaatcgaatcctggtgcggcg
atggcagtataggccgtaatccgcggttcgactatatactcgtcataataccactattacaacgaaacaaattaagactattcgacgttagcttaggaccacgccgc

intimin (eaeA) N terminus

1,930 1,940 1,950 1,960 1,970 1,980 1,990 2,000 2,010 2,020 2,030

accgttggtgtaaactatactccgattcctctggtgacgatggggatcgattaccgtcatggtacgggtaatgaaaatgatctcctttactcaatgcagttccgtta
tggcaaccacatttgatatgaggctaaggagaccactgctacccctagctaatggcagtaccatgcccattacttttactagaggaaatgagttacgtcaaggcaat

intimin (eaeA) N terminus

2,040 2,050 2,060 2,070 2,080 2,090 2,100 2,110 2,120 2,130 2,140

tcagtttgataaatcgtggtctcagcaaattgaaccacagtatgttaacgagttaagaacattatcaggcagccgttacgatctggttcagcgtaataacaatatta
agtcaaactatttagcaccagagtcgtttaacttggtgtcatacaattgctcaattcttgtaatagtccgtcggcaatgctagaccaagtcgcattattgttataat

intimin (eaeA) N terminus

2,150 2,160 2,170 2,180 2,190 2,200 2,210 2,220 2,230 2,240

ttctggagtacaagaagcaggatattctttctctgaatattccgcatgatattaatggtactgaacacagtacgcagaagattcagttgatcgttaagagcaaatac
aagacctcatgttcttcgtcctataagaaagagacttataaggcgtactataattaccatgacttgtgtcatgcgtcttctaagtcaactagcaattctcgtttatg

intimin (eaeA) N terminus

2,250 2,260 2,270 2,280 2,290 2,300 2,310 2,320 2,330 2,340 2,350

ggtctggatcgtatcgtctgggatgatagtgcattacgcagtcagggcggtcagattcagcatagcggaagccaaagcgcacaagactaccaggctattttgcctgc
ccagacctagcatagcagaccctactatcacgtaatgcgtcagtcccgccagtctaagtcgtatcgccttcggtttcgcgtgttctgatggtccgataaaacggacg

intimin (eaeA) N terminus

2,360 2,370 2,380 2,390 2,400 2,410 2,420 2,430 2,440 2,450 2,460

ttatgtgcaaggtggcagcaatatttataaagtgacggctcgcgcctatgaccgtaatggcaatagctctaacaatgtacagcttactattaccgttctgtcgaatg
aatacacgttccaccgtcgttataaatatttcactgccgagcgcggatactggcattaccgttatcgagattgttacatgtcgaatgataatggcaagacagcttac

intimin (eaeA) N terminus

2,470 2,480 2,490 2,500 2,510 2,520 2,530 2,540 2,550 2,560

gtcaagttgtcgaccaggttggggtaacggactttacggcggataagacttcggctaaagcggataacgccgataccattacttataccgcgacggtgaaaaagaat
cagttcaacagctggtccaaccccattgcctgaaatgccgcctattctgaagccgatttcgcctattgcggctatggtaatgaatatggcgctgccactttttctta

intimin (eaeA) N terminus

2,570 2,580 2,590 2,600 2,610 2,620 2,630 2,640 2,650 2,660 2,670

ggggtagctcaggctaatgtccctgtttcatttaatattgtttcaggaactgcaactcttggggcaaatagtgccaaaacggatgctaacggtaaggcaaccgtaac
ccccatcgagtccgattacagggacaaagtaaattataacaaagtccttgacgttgagaaccccgtttatcacggttttgcctacgattgccattccgttggcattg

intimin (eaeA) N terminus

2,680 2,690 2,700 2,710 2,720 2,730 2,740 2,750 2,760 2,770 2,780

gttgaagtcgagtacgccaggacaggtcgtcgtgtctgctaaaaccgcggagatgacttcagcacttaatgccagtgcggttatatttttgatggtgcgactagag
caacttcagctcatgcggtcctgtccagcagcacagacgattttggcgcctctactgaagtcgtgaattacggtcacgccaatataaaaaactaccacgctgatctc

T R
28...

intimin (eaeA) N terminus

2,790 2,800 2,810 2,820 2,830 2,840 2,850 2,860 2,870 2,880

aaccagaggcttaaAAAGAGGAGAAAGGTACCATGAGCAAAGGAGAAGAACTTTTCACTGGAGTTGTCCCAATTCTTGTTGAATTAGATGGTGATGTTAATGGGCAC
ttggtctccgaattTTTCTCCTCTTTCCATGGTACTCGTTTCCTCTTCTTGAAAAGTGACCTCAACAGGGTTAAGAACAACTTAATCTACCACTACAATTACCCGTG

E P E A * M S K G E E L F T G V V P I L V E L D G D V N G H
2883-2903 2922-3635

Inserted GFP segment
EP...n RBS sfGFP

2,890 2,900 2,910 2,920 2,930 2,940 2,950 2,960 2,970 2,980 2,990

AAATTTTCTGTCCGTGGAGAGGGTGAAGGTGATGCTACAAACGGAAAACTCACCCTTAAATTTATTTGCACTACTGGAAAACTACCTGTTCCGTGGCCAACACTTGT
TTTAAAAGACAGGCACCTCTCCCACTTCCACTACGATGTTTGCCTTTTGAGTGGGAATTTAAATAAACGTGATGACCTTTTGATGGACAAGGCACCGGTTGTGAACA

K F S V R G E G E G D A T N G K L T L K F I C T T G K L P V P W P T L V
2922-3635

Inserted GFP segment
sfGFP

3,000 3,010 3,020 3,030 3,040 3,050 3,060 3,070 3,080 3,090 3,100

CACTACTCTGACCTATGGTGTTCAATGCTTTTCCCGTTATCCGGATCACATGAAACGGCATGACTTTTTCAAGAGTGCCATGCCCGAAGGTTATGTACAGGAACGCA
GTGATGAGACTGGATACCACAAGTTACGAAAAGGGCAATAGGCCTAGTGTACTTTGCCGTACTGAAAAAGTTCTCACGGTACGGGCTTCCAATACATGTCCTTGCGT
T T L T Y G V Q C F S R Y P D H M K R H D F F K S A M P E G Y V Q E R
2922-3635
Inserted GFP segment
sfGFP
3,110 3,120 3,130 3,140 3,150 3,160 3,170 3,180 3,190 3,200 3,210

CTATATCTTTCAAAGATGACGGGACCTACAAGACGCGTGCTGAAGTCAAGTTTGAAGGTGATACCCTTGTTAATCGTATCGAGTTAAAGGGTATTGATTTTAAAGAA
GATATAGAAAGTTTCTACTGCCCTGGATGTTCTGCGCACGACTTCAGTTCAAACTTCCACTATGGGAACAATTAGCATAGCTCAATTTCCCATAACTAAAATTTCTT
T I S F K D D G T Y K T R A E V K F E G D T L V N R I E L K G I D F K E
2922-3635
Inserted GFP segment
sfGFP
3,220 3,230 3,240 3,250 3,260 3,270 3,280 3,290 3,300 3,310

GATGGAAACATTCTTGGACACAAACTCGAGTACAACTTTAACTCACACAATGTATACATCACGGCAGACAAACAAAAGAATGGAATCAAAGCTAACTTCAAAATTCG
CTACCTTTGTAAGAACCTGTGTTTGAGCTCATGTTGAAATTGAGTGTGTTACATATGTAGTGCCGTCTGTTTGTTTTCTTACCTTAGTTTCGATTGAAGTTTTAAGC
D G N I L G H K L E Y N F N S H N V Y I T A D K Q K N G I K A N F K I R
2922-3635
Inserted GFP segment
sfGFP
3,320 3,330 3,340 3,350 3,360 3,370 3,380 3,390 3,400 3,410 3,420

CCACAACGTTGAAGATGGTTCCGTTCAACTAGCAGACCATTATCAACAAAATACTCCAATTGGCGATGGCCCTGTCCTTTTACCAGACAACCATTACCTGTCGACAC
GGTGTTGCAACTTCTACCAAGGCAAGTTGATCGTCTGGTAATAGTTGTTTTATGAGGTTAACCGCTACCGGGACAGGAAAATGGTCTGTTGGTAATGGACAGCTGTG
H N V E D G S V Q L A D H Y Q Q N T P I G D G P V L L P D N H Y L S T
2922-3635
Inserted GFP segment
sfGFP
3,430 3,440 3,450 3,460 3,470 3,480 3,490 3,500 3,510 3,520 3,530

AATCTGTCCTTTCGAAAGATCCCAACGAAAAGCGTGACCACATGGTCCTTCTTGAGTTTGTAACTGCTGCTGGGATTACACATGGCATGGATGAGCTCTACAAAaag
TTAGACAGGAAAGCTTTCTAGGGTTGCTTTTCGCACTGGTGTACCAGGAAGAACTCAAACATTGACGACGACCCTAATGTGTACCGTACCTACTCGAGATGTTTttc
Q S V L S K D P N E K R D H M V L L E F V T A A G I T H G M D E L Y K
2922-3635
Inserted GFP segment
sfGFP
3,540 3,550 3,560 3,570 3,580 3,590 3,600 3,610 3,620 3,630

cttgacctgtgaagtgaaaaatggcgcacattgtgcgacatttttttgtctgccgtttaccgctactgcgtcacggatccccacgcgccctgtagcggcgcattaa
gaactggacacttcactttttaccgcgtgtaacacgctgtaaaaaaacagacggcaaatggcgatgacgcagtgcctaggggtgcgcgggacatcgccgcgtaatt
Inserted GFP segment
3,640 3,650 3,660 3,670 3,680 3,690 3,700 3,710 3,720 3,730 3,740

tactagagtcacactggctcaccttcgggtgggcctttctgcgtttatatactagtagcggccgctgcagtccggcaaaaaagggcaaggtgtcaccaccctgccct
atgatctcagtgtgaccgagtggaagcccacccggaaagacgcaaatatatgatcatcgccggcgacgtcaggccgttttttcccgttccacagtggtgggacggga

T7Te terminator BioBrick suffix his operon terminator

3,750 3,760 3,770 3,780 3,790 3,800 3,810 3,820 3,830 3,840 3,850

ttttctttaaaaccgaaaagattacttcgcgttatgcaggcttcctcgctcactgactcgctgcgctcggtcgttcggctgcggcgagcggtatcagctcactcaaa
aaaagaaattttggcttttctaatgaagcgcaatacgtccgaaggagcgagtgactgagcgacgcgagccagcaagccgacgccgctcgccatagtcgagtgagttt

his ope...inator L4440

3,860 3,870 3,880 3,890 3,900 3,910 3,920 3,930 3,940 3,950

ggcggtaatctcgaggttacattgtcgatctgttcatggtgaacagctttgaatgcaccaaaaactcgtaaaagctctgatgtatctatcttttttacaccgttttc
ccgccattagagctccaatgtaacagctagacaagtaccacttgtcgaaacttacgtggtttttgagcattttcgagactacatagatagaaaaaatgtggcaaaag

3,960 3,970 3,980 3,990 4,000 4,010 4,020 4,030 4,040 4,050 4,060

atctgtgcatatggacagttttccctttgatatgtaacggtgaacagttgttctactttgtttgttagtcttgatgcttcactgatagatacaagagccataagaa
tagacacgtatacctgtcaaaagggaaactatacattgccacttgtcaacaagatgaaacaaacaatcagaactacgaagtgactatctatgttctcggtattctt

4,070 4,080 4,090 4,100 4,110 4,120 4,130 4,140 4,150 4,160 4,170

cctcagatccttccgtatttagccagtatgttctctagtgtggttcgttgttttgcgtgagccatgagaacgaaccattgagatcatacttactttgcatgtcact
ggagtctaggaaggcataaatcggtcatacaagagatcacaccaagcaacaaaaacgcactcggtactcttgcttggtaactctagtatgaatgaaacgtacagtga

* I R G Y K A L I N E L T T R Q K Q T L W S F S G N L D Y K S Q M D S

4176-5126

Rep101

4,180 4,190 4,200 4,210 4,220 4,230 4,240 4,250 4,260 4,270 4,280

caaaaattttgcctcaaaactggtgagctgaatttttgcagttaaagcatcgtgtagtgtttttcttagtccgttatgtaggtaggaatctgatgtaatggttgttg
gtttttaaaacggagttttgaccactcgacttaaaaacgtcaatttcgtagcacatcacaaaaagaatcaggcaatacatccatccttagactacattaccaacaac

L F K A E F S T L Q I K A T L A D H L T K R L G N H L Y S D S T I T T P

4176-5126

Rep101

4,290 4,300 4,310 4,320 4,330 4,340 4,350 4,360 4,370 4,380

gtattttgtcaccattcatttttatctggttgttctcaagttcggttacgagatccatttgtctatctagttcaacttggaaaatcaacgtatcagtcgggcggcct
cataaaacagtggtaagtaaaaatagaccaacaagagttcaagccaatgctctaggtaaacagatagatcaagttgaaccttttagttgcatagtcagcccgccgga

I K D G N M K I Q N N E L E T V L D M Q R D L E V Q F I L T D T P R G

4176-5126

Rep101

4,390 4,400 4,410 4,420 4,430 4,440 4,450 4,460 4,470 4,480 4,490

cgcttatcaaccaccaatttcatattgctgtaagtgtttaaatctttacttattggtttcaaaacccattggttaagccttttaaactcatggtagttattttcaag
gcgaatagttggtggttaaagtataacgacattcacaaatttagaaatgaataaccaaagttttgggtaaccaattcggaaaatttgagtaccatcaataaaagttc
F K D V V L K M N S Y T N L D K S I P K L V W Q N L R K F E H Y N N E L
4176-5126
Rep101
4,500 4,510 4,520 4,530 4,540 4,550 4,560 4,570 4,580 4,590 4,600

cattaacatgaacttaaattcatcaaggctaatctctatatttgccttgtgagttttcttttgtgttagttcttttaataaccactcataaatcctcatagagtatt
gtaattgtacttgaatttaagtagttccgattagagatataaacggaacactcaaaagaaaacacaatcaagaaaattattggtgagtatttaggagtatctcataa
M L M F K F E D L S I E I N A K H T K K Q T L E K L L W E Y I R M S Y K
4176-5126
Rep101
4,610 4,620 4,630 4,640 4,650 4,660 4,670 4,680 4,690 4,700

tgttttcaaaagacttaacatgttccagattatattttatgaatttttttaactggaaaagataaggcaatatctcttcactaaaactaattctaatttttcgctt
acaaaagttttctgaattgtacaaggtctaatataaaatacttaaaaaaattgaccttttctattccgttatagagaagtgattttgattaagattaaaaagcgaa
N E F S K V H E L N Y K I F K K L Q F L Y P L I E E S F V L E L K E S
4176-5126
Rep101
4,710 4,720 4,730 4,740 4,750 4,760 4,770 4,780 4,790 4,800 4,810

gagaacttggcatagtttgtccactggaaaatctcaaagcctttaaccaaaggattcctgatttccacagttctcgtcatcagctctctggttgctttagctaatac
ctcttgaaccgtatcaaacaggtgaccttttagagtttcggaaattggtttcctaaggactaaaggtgtcaagagcagtagtcgagagaccaacgaaatcgattatg
S F K A Y N T W Q F I E F G K V L P N R I E V T R T M L E R T A K A L V
4176-5126
Rep101
4,820 4,830 4,840 4,850 4,860 4,870 4,880 4,890 4,900 4,910 4,920

accataagcattttccctactgatgttcatcatctgagcgtattggttataagtgaacgataccgtccgttctttccttgtagggttttcaatcgtggggttgagta
tggtattcgtaaaagggatgactacaagtagtagactcgcataaccaatattcacttgctatggcaggcaagaaaggaacatcccaaaagttagcaccccaactcat
G Y A N E R S I N M M Q A Y Q N Y T F S V T R E K R T P N E I T P N L L
4176-5126
Rep101
4,930 4,940 4,950 4,960 4,970 4,980 4,990 5,000 5,010 5,020

gtgccacacagcataaaattagcttggtttcatgctccgttaagtcatagcgactaatcgctagttcatttgctttgaaaacaactaattcagacatacatctcaat
cacggtgtgtcgtattttaatcgaaccaaagtacgaggcaattcagtatcgctgattagcgatcaagtaaacgaaacttttgttgattaagtctgtatgtagagtta
A V C C L I L K T E H E T L D Y R S I A L E N A K F V V L E S M
4176-5126
Rep101
5,030 5,040 5,050 5,060 5,070 5,080 5,090 5,100 5,110 5,120 5,130

tggtctaggtgattttaatcactataccaattgagatgggctagtcaatgataattacatgtccttttcctttgagttgtgggtatctgtaaattctgctagacctt
accagatccactaaaattagtgatatggttaactctacccgatcagttactattaatgtacaggaaaaggaaactcaacacccatagacatttaagacgatctggaa

pSC101 ori

5,140 5,150 5,160 5,170 5,180 5,190 5,200 5,210 5,220 5,230 5,240

tgctggaaaacttgtaaattctgctagaccctctgtaaattccgctagacctttgtgtgttttttttgtttatattcaagtggttataatttatagaataaagaaag
acgaccttttgaacatttaagacgatctgggagacatttaaggcgatctggaaacacacaaaaaaaacaaatataagttcaccaatattaaatatcttatttctttc

pSC101 ori

5,250 5,260 5,270 5,280 5,290 5,300 5,310 5,320 5,330 5,340 5,350

aataaaaaaagataaaaagaatagatcccagccctgtgtataactcactactttagtcagttccgcagtattacaaaaggatgtcgcaaacgctgtttgctcctcta
ttatttttttctatttttcttatctagggtcgggacacatattgagtgatgaaatcagtcaaggcgtcataatgttttcctacagcgtttgcgacaaacgaggagat

pSC101 ori

5,360 5,370 5,380 5,390 5,400 5,410 5,420 5,430 5,440 5,450

caaaacagaccttaaaaccctaaaggcttaagtagcaccctcgcaagctcgggcaaatcgctgaatattccttttgtctccgaccatcaggcacctgagtcgctgtc
gttttgtctggaattttgggatttccgaattcatcgtgggagcgttcgagcccgtttagcgacttataaggaaaacagaggctggtagtccgtggactcagcgacag

5,460 5,470 5,480 5,490 5,500 5,510 5,520 5,530 5,540 5,550 5,560

tttttcgtgacattcagttcgctgcgctcacggctctggcagtgaatgggggtaaatggcactacaggcgccttttatggattcatgcaaggaaactacccataata
aaaaagcactgtaagtcaagcgacgcgagtgccgagaccgtcacttacccccatttaccgtgatgtccgcggaaaatacctaagtacgttcctttgatgggtattat

5,570 5,580 5,590 5,600 5,610 5,620 5,630 5,640 5,650 5,660 5,670

caagaaaagcccgtcacgggcttctcagggcgttttatggcgggtctgctatgtggtgctatctgactttttgctgttcagcagttcctgccctctgattttccagt
gttcttttcgggcagtgcccgaagagtcccgcaaaataccgcccagacgatacaccacgatagactgaaaaacgacaagtcgtcaaggacgggagactaaaaggtca

5,680 5,690 5,700 5,710 5,720 5,730 5,740 5,750 5,760 5,770

ctgaccacttcggattatcccgtgacaggtcattcagactggctaatgcacccagtaaggcagcggtatcatcaacaggcttacccgtcttactgtccctagtgctt
gactggtgaagcctaatagggcactgtccagtaagtctgaccgattacgtgggtcattccgtcgccatagtagttgtccgaatgggcagaatgacagggatcacgaa

5,780 5,790 5,800 5,810 5,820 5,830 5,840 5,850 5,860 5,870 5,880

ggattctcaccaataaaaaacgcccggcggcaaccgagcgttctgaacaaatccagatggagttctgaggtcattactggatctatcaacaggagtccaagcgagct
cctaagagtggttattttttgcgggccgccgttggctcgcaagacttgtttaggtctacctcaagactccagtaatgacctagatagttgtcctcaggttcgctcga

lambda t0 terminator

5,890 5,900 5,910 5,920 5,930 5,940 5,950 5,960 5,970 5,980 5,990

cgtaaacttggtctgacagataactgccttaaaaaaattacgccccgccctgccactcatcgcagtactgttgtaattcattaagcattctgccgacatggaagcca
gcatttgaaccagactgtctattgacggaatttttttaatgcggggcgggacggtgagtagcgtcatgacaacattaagtaattcgtaagacggctgtaccttcggt

Space CamR

6,000 6,010 6,020 6,030 6,040 6,050 6,060 6,070 6,080 6,090

tcacagacggcatgatgaacctgaatcgccagcggcatcagcaccttgtcgccttgcgtataatatttgcccatggtgaaaacgggggcgaagaagttgtccatatt
agtgtctgccgtactacttggacttagcggtcgccgtagtcgtggaacagcggaacgcatattataaacgggtaccacttttgcccccgcttcttcaacaggtataa
CamR
6,100 6,110 6,120 6,130 6,140 6,150 6,160 6,170 6,180 6,190 6,200

ggccacgtttaaatcaaaactggtgaaactcacccagggattggctgagacgaaaaacatattctcaataaaccctttagggaaataggccaggttttcaccgtaac
ccggtgcaaatttagttttgaccactttgagtgggtccctaaccgactctgctttttgtataagagttatttgggaaatccctttatccggtccaaaagtggcattg
CamR
6,210 6,220 6,230 6,240 6,250 6,260 6,270 6,280 6,290 6,300 6,310

acgccacatcttgcgaatatatgtgtagaaactgccggaaatcgtcgtggtattcactccagagcgatgaaaacgtttcagtttgctcatggaaaacggtgtaacaa
tgcggtgtagaacgcttatatacacatctttgacggcctttagcagcaccataagtgaggtctcgctacttttgcaaagtcaaacgagtaccttttgccacattgtt
CamR
6,320 6,330 6,340 6,350 6,360 6,370 6,380 6,390 6,400 6,410 6,420

gggtgaacactatcccatatcaccagctcaccgtctttcattgccatacggaactccggatgagcattcatcaggcgggcaagaatgtgaataaaggccggataaaa
cccacttgtgatagggtatagtggtcgagtggcagaaagtaacggtatgccttgaggcctactcgtaagtagtccgcccgttcttacacttatttccggcctatttt
CamR
6,430 6,440 6,450 6,460 6,470 6,480 6,490 6,500 6,510 6,520

cttgtgcttatttttctttacggtctttaaaaaggccgtaatatccagctgaacggtctggttataggtacattgagcaactgactgaaatgcctcaaaatgttctt
gaacacgaataaaaagaaatgccagaaatttttccggcattataggtcgacttgccagaccaatatccatgtaactcgttgactgactttacggagttttacaagaa
CamR
6,530 6,540 6,550 6,560 6,570 6,580 6,590 6,600 6,610 6,620 6,630

tacgatgccattgggatatatcaacggtggtatatccagtgatttttttctccattttagcttccttagctcctgaaaatctcgataactcaaaaaatacgcccggt
atgctacggtaaccctatatagttgccaccatataggtcactaaaaaaagaggtaaaatcgaaggaatcgaggacttttagagctattgagttttttatgcgggcca
CamR
6,640 6,650 6,660 6,670 6,680 6,690 6,700 6,710 6,720 6,730 6,740

agtgatcttatttcattatggtgaaagttggaacctcacccaacgtctcgacatgagcggatacatatttgaatgtatttagaaaaataaacaaataggggttccgc
tcactagaataaagtaataccactttcaaccttggagtgggttgcagagctgtactcgcctatgtataaacttacataaatctttttatttgtttatccccaaggcg
CamR
6,750 6,760 6,770 6,780 6,790 6,800 6,810 6,820 6,830 6,840

gcacatttccccgaaaagtgccacctgaaattgtaaacgttaatattttgttaaaattcgcgttaaatttttgttaaatcagctcattttttaaccaataggccgaa
cgtgtaaaggggcttttcacggtggactttaacatttgcaattataaaacaattttaagcgcaatttaaaaacaatttagtcgagtaaaaaattggttatccggctt
CamR
6,850 6,860 6,870 6,880 6,890 6,900 6,910 6,920 6,930 6,940 6,950

```
atcggcaaaatcccttataaatcaaaagaatagaccgagatagggttgagtgcacatttcccatggtgccacctgacgtctaagaaaccattattatcatgacatta
tagccgttttagggaatatttagttttcttatctggctctatcccaactcacgtgtaaagggtaccacggtggactgcagattctttggtaataatagtactgtaat
```

CamR

6,960 6,970 6,980 6,990 7,000 7,010 7,020 7,030 7,040 7,050 7,060

```
acctataaaaataggcgtatcacgaggcagaatttcagataaaaaaaatccttagctttcgctaaggatgatttctggaattcgcggccgcttctagag
tggatatttttatccgcatagtgctccgtcttaaagtctattttttttaggaatcgaaagcgattcctactaaagaccttaagcgccggcgaagatctc
```

pBRforEco bacterial terminator BioBrick prefix

7,070 7,080 7,090 7,100 7,110 7,120 7,130 7,140 7,150 7,160
