# Supplementary material for: Reprogrammed SimCells for antimicrobial therapy
Source: Proc Natl Acad Sci U S A. 2026 Mar 17;123(12):e2517118123. doi: 10.1073/pnas.2517118123 (PMC13012131; doi:10.1073/pnas.2517118123)
Supplement: Supplementary file 3 — Dataset S02 (PDF) [file pnas.2517118123.sd02.pdf]

pRH12x (4699 bp)

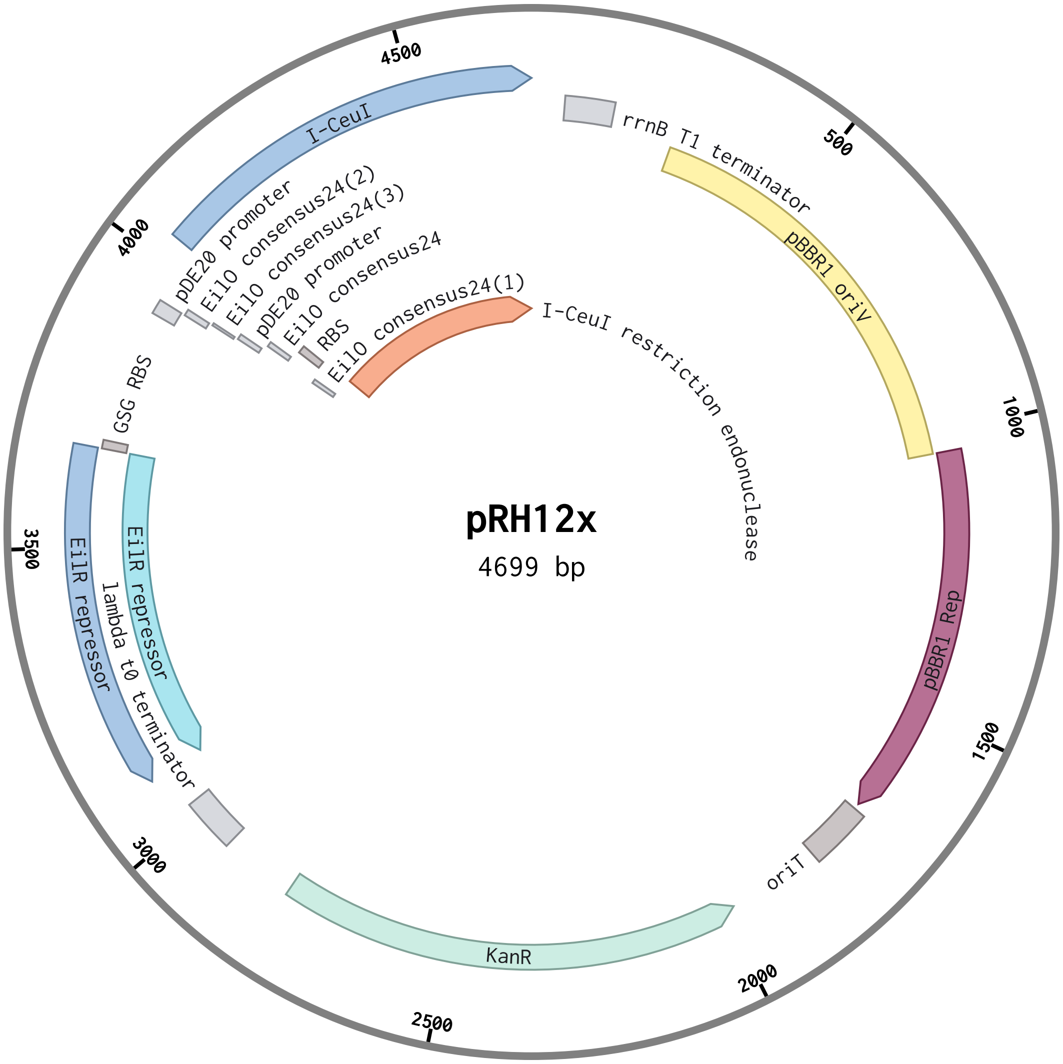

pRH12x (4699 bp)

cttaaGGGCCCATCCTAGGctcctgtgtgaaattgttatccgctttaattaaaggcatcaaataaaacgaaaggctcagtcgaaagactgggcctttcgttttatct
gaattCCCGGGTAGGATCCgaggacacactttaacaataggcgaaattaatttccgtagtttattttgctttccgagtcagctttctgacccggaaagcaaaataga

rrnB T1 terminator

10 20 30 40 50 60 70 80 90 100

gttgtttgtcggtgaacgctctcctgagtaggacaaatccgccgccctagacagctgggcgcgccccccctacgggcttgctctccgggcttcgccctgcgcggtcg
caacaaacagccacttgcgagaggactcatcctgtttaggcggcgggatctgtcgacccgcgcgggggggatgcccgaacgagaggcccgaagcgggacgcgccagc

rrnB T1 terminator

110 120 130 140 150 160 170 180 190 200 210

ctgcgctcccttgccagcccgtggatatgtggacgatggccgcgagcggccaccggctggctcgcttcgctcggcccgtggacaaccctgctggacaagctgatgga
gacgcgagggaacggtcgggcacctatacacctgctaccggcgctcgccggtggccgaccgagcgaagcgagccgggcacctgttgggacgacctgttcgactacct

pBBR1 oriV

220 230 240 250 260 270 280 290 300 310 320

caggctgcgcctgcccacgagcttgaccacagggattgcccaccggctacccagccttcgaccacatacccaccggctccaactgcgcggcctgcggccttgcccca
gtccgacgcggacgggtgctcgaactggtgtccctaacgggtggccgatgggtcggaagctggtgtatgggtggccgaggttgacgcgccggacgccggaacggggt

pBBR1 oriV

330 340 350 360 370 380 390 400 410 420

tcaatttttttaattttctctggggaaaagcctccggcctgcggcctgcgcgcttcgcttgccggttggacaccaagtggaaggcgggtcaaggctcgcgcagcgac
agttaaaaaaattaaaagagaccccttttcggaggccggacgccggacgcgcgaagcgaacggccaacctgtggttcaccttccgcccagttccgagcgcgtcgctg

pBBR1 oriV

430 440 450 460 470 480 490 500 510 520 530

cgcgcagcggcttggccttgacgcgcctggaacgacccaagcctatgcgagtgggggcagtcgaagggcgaagcccgcccgcctgcccccgagcctcacggcggcg
gcgcgtcgccgaaccggaactgcgcggaccttgctgggttcggatacgctcacccccgtcagcttcccgcttcgggcgggcggacgggggctcggagtgccgccgc

pBBR1 oriV

540 550 560 570 580 590 600 610 620 630 640

agtgcgggggttccaagggggcagcgccaccttgggcaaggccgaaggccgcgcagtcgatcaacaagccccggaggggccactttttgccggagggggagccgcgc
tcacgcccccaaggttcccccgtcgcggtggaacccgttccggcttccggcgcgtcagctagttgttcggggcctccccggtgaaaaacggcctccccctcggcgcg

pBBR1 oriV

650 660 670 680 690 700 710 720 730 740

cgaaggcgtgggggaaccccgcaggggtgcccttctttgggcaccaaagaactagatatagggcgaaatgcgaaagacttaaaaatcaacaacttaaaaaaggggg
gcttccgcacccccttggggcgtccccacgggaagaaacccgtggtttcttgatctatatcccgctttacgctttctgaatttttagttgttgaatttttttcccccc

pBBR1 oriV

750 760 770 780 790 800 810 820 830 840 850

tacgcaacagctcattgcggcacccccccgcaatagctcattgcgtaggttaaagaaaatctgtaattgactgccacttttacgcaacgcataattgttgtcgcgctg
atgcgttgtcgagtaacgccgtgggggggcgttatcgagtaacgcatccaatttcttttagacattaactgacggtgaaaatgcgttgcgtattaacaacagcgcgac

pBBR1 oriV

860 870 880 890 900 910 920 930 940 950 960

ccgaaaagttgcagctgattgcgcatggtgccgcaaccgtgcggcacccctaccgcatggagataagcatggccacgcagtccagagaaatcggcattcaagccaag
ggcttttcaacgtcgactaacgcgtaccacggcgttggcacgccgtggggatggcgtacctctattcgtaccggtgcgtcaggtctctttagccgtaagttcggttc

pBBR1 oriV | pBBR1 Rep

970 980 990 1,000 1,010 1,020 1,030 1,040 1,050 1,060 1,070

aacaagcccggtcactgggtgcaaacggaacgcaaagcgcatgaggcgtgggccgggcttattgcgaggaaacccacggcggcaatgctgctgcatcacctcgtggc
ttgttcgggccagtgacccacgtttgccttgcgtttcgcgtactccgcacccggcccgaataacgctcctttgggtgccgccgttacgacgacgtagtggagcaccg

pBBR1 Rep

1,080 1,090 1,100 1,110 1,120 1,130 1,140 1,150 1,160 1,170

gcagatgggccaccagaacgccgtggtggtcagccagaagacactttccaagctcatcggacgttctttgcggacggtccaatacgcagtcaaggacttggtggccg
cgtctacccggtggtcttgcggcaccaccagtcggtcttctgtgaaaggttcgagtagcctgcaagaaacgcctgccaggttatgcgtcagttcctgaaccaccggc

pBBR1 Rep

1,180 1,190 1,200 1,210 1,220 1,230 1,240 1,250 1,260 1,270 1,280

agcgctggatctccgtcgtgaagctcaacggccccggcaccgtgtcggcctacgtggtcaatgaccgcgtggcgtggggccagccccgcgaccagttgcgcctgtcg
tcgcgacctagaggcagcacttcgagttgccggggccgtggcacagccggatgcaccagttactggcgcaccgcaccccggtcggggcgctggtcaacgcggacagc

pBBR1 Rep

1,290 1,300 1,310 1,320 1,330 1,340 1,350 1,360 1,370 1,380 1,390

gtgttcagtgccgccgtggtggttgatcacgacgaccaggacgaatcgctgttggggcatggcgacctgcgccgcatcccgaccctgtatccgggcgagcagcaact
cacaagtcacggcggcaccaccaactagtgctgctggtcctgcttagcgacaaccccgtaccgctggacgcggcgtagggctgggacataggcccgctcgtcgttga

pBBR1 Rep

1,400 1,410 1,420 1,430 1,440 1,450 1,460 1,470 1,480 1,490

accgaccggccccggcgaggagccgcccagccagcccggcattccgggcatggaaccagacctgccagccttgaccgaaacggaggaatgggaacggcgcgggcagc
tggctggccggggccgctcctcggcgggtcggtcgggccgtaaggcccgtaccttggtctggacggtcggaactggctttgcctccttacccttgccgcgcccgtcg

pBBR1 Rep

1,500 1,510 1,520 1,530 1,540 1,550 1,560 1,570 1,580 1,590 1,600

agcgcctgccgatgcccgatgagccgtgttttctggacgatggcgagccgttggagccgccgacacgggtaacgctgccgcgccggtagggccggcctacggccagc
tcgcggacggctacgggctactcggcacaaaagacctgctaccgctcggcaacctcggcggctgtgcccattgcgacggcgcggccatcccggccggatgccggtcg

pBBR1 Rep

1,610 1,620 1,630 1,640 1,650 1,660 1,670 1,680 1,690 1,700 1,710

ctcgcagagcaggattcccgttgagcaccgccaggtgcgaataagggacagtgaagaaggaacacccgctcgcgggtgggcctacttcacctatcctgcccggctga
gagcgtctcgtcctaagggcaactcgtggcggtccacgcttattccctgtcacttcttccttgtgggcgagcgcccacccggatgaagtggataggacgggccgact

oriT

1,720 1,730 1,740 1,750 1,760 1,770 1,780 1,790 1,800 1,810

cgccgttggatacaccaaggaaagtctacacgaaccctttggcaaaatcctgtatatcgtgcgaaaaaggatggatataccgaaaaaatcgctataatgaccccgaa
gcggcaacctatgtggttcctttcagatgtgcttgggaaaccgttttaggacatatagcacgctttttcctacctatatggcttttttagcgatattactggggctt

1,820 1,830 1,840 1,850 1,860 1,870 1,880 1,890 1,900 1,910 1,920

gcagggttatgcagcggaaaaggacaacgcgcggaccgcggtccaattaattattagaaaaattcatccagcatcagatgaaattgcagtttgttcatatccggatt
cgtcccaatacgtcgccttttcctgttgcgcgcctggcgccaggttaattaataatctttttaagtaggtcgtagtctactttaacgtcaaacaagtataggcctaa

KanR

1,930 1,940 1,950 1,960 1,970 1,980 1,990 2,000 2,010 2,020 2,030

atcaatgccatatttctgaaacagacgtttttgcaggctcgggctaaattcgcccaggcagttccacagaatggccagatcctgataacgatccgcaatgcccacac
tagttacggtataaagactttgtctgcaaaaacgtccgagcccgatttaagcgggtccgtcaaggtgtcttaccggtctaggactattgctaggcgttacgggtgtg

KanR

2,040 2,050 2,060 2,070 2,080 2,090 2,100 2,110 2,120 2,130 2,140

ggcccacatcaatgcagccaatcagtttgccttcatcgaaaatcaggttatccaggctaaaatcgccgtgggtcaccacgctatccgggctaaacggcagcagttta
ccgggtgtagttacgtcggttagtcaaacggaagtagcttttagtccaataggtccgattttagcggcacccagtggtgcgataggcccgatttgccgtcgtcaaat

KanR

2,150 2,160 2,170 2,180 2,190 2,200 2,210 2,220 2,230 2,240

tgcatttctttccacacctgttccaccggccagccgttacgttcatcatcaaaatcgctcgcatccaccaggccgttgttcatacggctctgcgcctgggccagacg
acgtaaagaaaggtgtggacaaggtggccggtcggcaatgcaagtagtagttttagcgagcgtaggtggtccggcaacaagtatgccgagacgcggacccggtctgc

KanR

2,250 2,260 2,270 2,280 2,290 2,300 2,310 2,320 2,330 2,340 2,350

aaacacacgatcgctgttaaacgggcagttgcacaccggaatgctatgcagacgacgcagaaacacggccagcgcatccacaatgttttcgccgctatccggatatt
tttgtgtgctagcgacaatttgcccgtcaacgtgtggccttacgatacgtctgctgcgtctttgtgccggtcgcgtaggtgttacaaaagcggcgataggcctataa

KanR

2,360 2,370 2,380 2,390 2,400 2,410 2,420 2,430 2,440 2,450 2,460

cttccagcacctgaaacgcggttttgcccggaatcgcggtggtcagcagccacgcatcatccggggtgcgaataaaatgtttaatggtcggcagcggcataaattcg
gaaggtcgtggactttgcgccaaaacgggccttagcgccaccagtcgtcggtgcgtagtaggccccacgcttattttacaaattaccagccgtcgccgtatttaagc

KanR

2,470 2,480 2,490 2,500 2,510 2,520 2,530 2,540 2,550 2,560

gtcagccagttcagacgcaccatttcatcggtcacatcgttcgccacgctgcctttgccatgtttcagaaacagttccggcgcatccggtttgccatacagacgata
cagtcggtcaagtctgcgtggtaaagtagccagtgtagcaagcggtgcgacggaaacggtacaaagtctttgtcaaggccgcgtaggccaaacggtatgtctgctat

KanR

2,570 2,580 2,590 2,600 2,610 2,620 2,630 2,640 2,650 2,660 2,670

aatggtcgcgccgctctgacccacgttatcacgcgcccatttatagccatacagatccgcatccatgttgctgttcagacgcggacggctacagctcgtttcacgct
ttaccagcgcggcgagactgggtgcaatagtgcgcgggtaaatatcggtatgtctaggcgtaggtacaacgacaagtctgcgcctgccgatgtcgagcaaagtgcga

KanR

2,680 2,690 2,700 2,710 2,720 2,730 2,740 2,750 2,760 2,770 2,780

gaatatggctcataacaccccttgtattactgtttatgtaagcagacagttttattgttcatgatgatatatttttatcttgtgcaatgtaacatcagagattttga
cttataccgagtattgtggggaacataatgacaaatacattcgtctgtcaaaataacaagtactactatataaaaatagaacacgttacattgtagtctctaaaact

KanR

2,790 2,800 2,810 2,820 2,830 2,840 2,850 2,860 2,870 2,880

gacacaaatttaaatcgtaattattggggacccctggattctcaccaataaaaaacgcccggcggcaaccgagcgttctgaacaaatccagatggagttctgaggtc
ctgtgtttaaatttagcattaataacccctggggacctaagagtggttattttttgcgggccgccgttggctcgcaagacttgtttaggtctacctcaagactccag

lambda t0 terminator

2,890 2,900 2,910 2,920 2,930 2,940 2,950 2,960 2,970 2,980 2,990

attactggatctatcaacaggagtccaagactagtcgccagggttttcccagtcacgacgcggccgcaagcttgcatgcctgcaggtgacgtcttacgaaaataact
taatgacctagatagttgtcctcaggttctgatcagcggtcccaaaagggtcagtgctgcgccggcgttcgaacgtacggacgtccactgcagaatgcttttattga

* S F L E

EilR repr...

lambda t0 terminator

EilR...sor

3,000 3,010 3,020 3,030 3,040 3,050 3,060 3,070 3,080 3,090 3,100

caagctgaataacgtgctgcaggtggcgggtgaatgcggcatcgtctacctccggcattcccagcacgtaaatcccgtccagtccacagaccagcgaaatcagccgc
gttcgacttattgcacgacgtccaccgcccacttacgccgtagcagatggaggccgtaagggtcgtgcatttagggcaggtcaggtgtctggtcgctttagtcggcg

L Q I V H Q L H R T F A A D D V E P M G L V Y I G D L G C V L S I L R

EilR repressor

EilR repressor

3,110 3,120 3,130 3,140 3,150 3,160 3,170 3,180 3,190 3,200 3,210

caggcgatattttcggcgctatcgcgcagggtaaattcgccagcggcatggcccgcgcgaatgatcctgaccgcttcgtcatgccacaggttcatggtcagcaggta
gtccgctataaaagccgcgatagcgcgtcccatttaagcggtcgccgtaccgggcgcgcttactaggactggcgaagcagtacggtgtccaagtaccagtcgtccat

W A I N E A S D R L T F E G A A H G A R I I R V A E D H W L N M T L L Y

EilR repressor

EilR repressor

3,220 3,230 3,240 3,250 3,260 3,270 3,280 3,290 3,300 3,310

cgcgcttttgatttccggatcgctgtcggccagcagctgcgcctggcgccacaggcggatatagggctccagccgtccgtcctcgctgccgagggcggaaaacagct
gcgcgaaaactaaaggcctagcgacagccggtcgtcgacgcggaccgcggtgtccgcctatatcccgaggtcggcaggcaggagcgacggctcccgccttttgtcga

A S K I E P D S D A L L Q A Q R W L R I Y P E L R G D E S G L A S F L Q

EilR repressor

EilR repressor

3,320 3,330 3,340 3,350 3,360 3,370 3,380 3,390 3,400 3,410 3,420

gttcacgccagccggcggtgcgcgaaaggcgctgcagatccatcatctcgcggatcacgcggataaacgcctgggattttagctcgccggaggaggtgaaatggtgg
caagtgcggtcggccgccacgcgctttccgcgacgtctaggtagtagagcgcctagtgcgcctatttgcggaccctaaaatcgagcggcctcctccactttaccacc

E R W G A T R S L R Q L D M M E R I V R I F A Q S K L E G S S T F H H

EilR repressor

EilR repressor

3,430 3,440 3,450 3,460 3,470 3,480 3,490 3,500 3,510 3,520 3,530

tggacctggcctgcggcgacgcccgcggcggtggcgatgttgcggaccgtcataccggtaaacccttgatccagcgcgacgcgcatggcggcctgcatgatggtttc
acctggaccggacgccgctgcgggcgccgccaccgctacaacgcctggcagtatggccatttgggaactaggtcgcgctgcgcgtaccgccggacgtactaccaaag

H V Q G A A V G A A T A I N R V T M G T F G Q D L A V R M A A Q M I T E

EilR repressor

EilR repressor

3,540 3,550 3,560 3,570 3,580 3,590 3,600 3,610 3,620 3,630

ccgacgttcttcgcgattcagatagcccatattgtcacctccttatccacacattatacgagccggatgattaatggggaaccttaccagagggcgccccagctggc
ggctgcaagaagcgctaagtctatcgggtataacagtggaggaataggtgtgtaatatgctcggcctactaattaccccttggaatggtctcccgcggggtcgaccg

R R E E R N L Y G M

EilR repressor

EilR repressor GSG RBS

3,640 3,650 3,660 3,670 3,680 3,690 3,700 3,710 3,720 3,730 3,740

aattcctcttaagtaagtaagagtatacgtatatcggctaataacgtattaaggcgcttcggcgccttttttttatgggggtattttcatcccaatccacacgtccaa
ttaaggagaattcattcattctcatatgcatatagccgattattgcataattccgcgaagccgcggaaaaaaaatacccccataaaagtagggttaggtgtgcaggtt

3,750 3,760 3,770 3,780 3,790 3,800 3,810 3,820 3,830 3,840 3,850

cgcacagcaaacaccacgtcgaccctatcagctgcgtgctttctatgagtcgttgctgcataacaaaaaactgcaaaaaatagtttgacaggacacgtgtccaactt
gcgtgtcgtttgtggtgcagctgggatagtcgacgcacgaaagatactcagcaacgacgtattgttttttgacgttttttatcaaactgtcctgtgcacaggttgaa

pDE20 promoter Eil...2)

3,860 3,870 3,880 3,890 3,900 3,910 3,920 3,930 3,940 3,950

taagatgtacccaaagttggacacgtgtccaactttgaattcaaaagatcttttaactttaagaaggagatatacatgagctcatgtcaaactttatacttaaaccg
attctacatgggtttcaacctgtgcacaggttgaaacttaagttttctagaaaattgaaattcttcctctatatgtactcgagtacagtttgaaatatgaatttggc

M S N F I L K P

I-CeuI

pDE...er | Eil...1) | Eil...24 | RBS | I-CeuI re...nuclease

3,960 3,970 3,980 3,990 4,000 4,010 4,020 4,030 4,040 4,050 4,060

ggcgaaaaactaccccaagacaaactagaagaattaaaaaaaattaatgatgctgttaaaaaaacgaaaaatttctcaaaatacttgattgacttaagaaaactttt
ccgctttttgatggggttctgtttgatcttcttaattttttttaattactacgacaatttttttgctttttaaagagttttatgaactaactgaattcttttgaaaa

G E K L P Q D K L E E L K K I N D A V K K T K N F S K Y L I D L R K L F

I-CeuI

I-CeuI restriction endonuclease

4,070 4,080 4,090 4,100 4,110 4,120 4,130 4,140 4,150 4,160 4,170

tcaaattgacgaagtccaagtaacttctgaatcaaaactctttttagctggtttttttagaaggtgaagcttctctaaatattagcactaaaaagctcgctacttcta
agtttaactgcttcaggttcattgaagacttagttttgagaaaaatcgaccaaaaaatcttccacttcgaagagatttataatcgtgatttttcgagcgatgaagat

Q I D E V Q V T S E S K L F L A G F L E G E A S L N I S T K K L A T S

I-CeuI

I-CeuI restriction endonuclease

4,180 4,190 4,200 4,210 4,220 4,230 4,240 4,250 4,260 4,270 4,280

aatttggtttggtggttgatcctgaattcaatgtgactcaacatgtcaatggggttaaagtgctttatttagcattagaagtatttaaaacagggcgtattcgtcat
ttaaaccaaaccaccaactaggacttaagttacactgagttgtacagttaccccaatttcacgaaataaatcgtaatcttcataaattttgtcccgcataagcagta

K F G L V V D P E F N V T Q H V N G V K V L Y L A L E V F K T G R I R H

I-CeuI

I-CeuI restriction endonuclease

4,290 4,300 4,310 4,320 4,330 4,340 4,350 4,360 4,370 4,380

aaaagtggtagtaatgcaactttagttttaactattgacaatcgtcaaagtttggaagaaaaagtaattccttttatgaacaatatgttgttgccttcagttctcc
ttttcaccatcattacgttgaaatcaaaattgataactgttagcagtttcaaaccttctttttcattaaggaaaaatacttgttatacaacaacggaagtcaagagg

K S G S N A T L V L T I D N R Q S L E E K V I P F Y E Q Y V V A F S S P

I-CeuI

I-CeuI restriction endonuclease

4,390 4,400 4,410 4,420 4,430 4,440 4,450 4,460 4,470 4,480 4,490

agaaaaagtcaaacgtgtagctaattttaaagctttgttagaattatttaataatgacgctcaccaagatttagaacaattggtaaacaaaatcctaccaatttggg
tctttttcagtttgcacatcgattaaaatttcgaaacaatcttaataaattattactgcgagtggttctaaatcttgttaaccatttgttttaggatggttaaaccc

E K V K R V A N F K A L L E L F N N D A H Q D L E Q L V N K I L P I W

I-CeuI

I-CeuI restriction endonuclease

4,500 4,510 4,520 4,530 4,540 4,550 4,560 4,570 4,580 4,590 4,600

atcaaatgcgtaaacaacaaggacaaagtaacgaaggctttcctaatttagaagcagctcaagactttgctcgtaattataaaaaaggtataaagtag
tagtttacgcatttgttgttcctgtttcattgcttccgaaaggattaaatcttcgtcgagttctgaaacgagcattaatatttttttccatatttcatc

D Q M R K Q Q G Q S N E G F P N L E A A Q D F A R N Y K K G I K *

I-CeuI

I-CeuI restriction endonuclease

4,610 4,620 4,630 4,640 4,650 4,660 4,670 4,680 4,690
