# Supplementary material for: Reprogrammed SimCells for antimicrobial therapy
Source: Proc Natl Acad Sci U S A. 2026 Mar 17;123(12):e2517118123. doi: 10.1073/pnas.2517118123 (PMC13012131; doi:10.1073/pnas.2517118123)
Supplement: Supplementary file 5 — Dataset S04 (PDF) [file pnas.2517118123.sd04.pdf]

pTet_Nb39 (6722 bp)

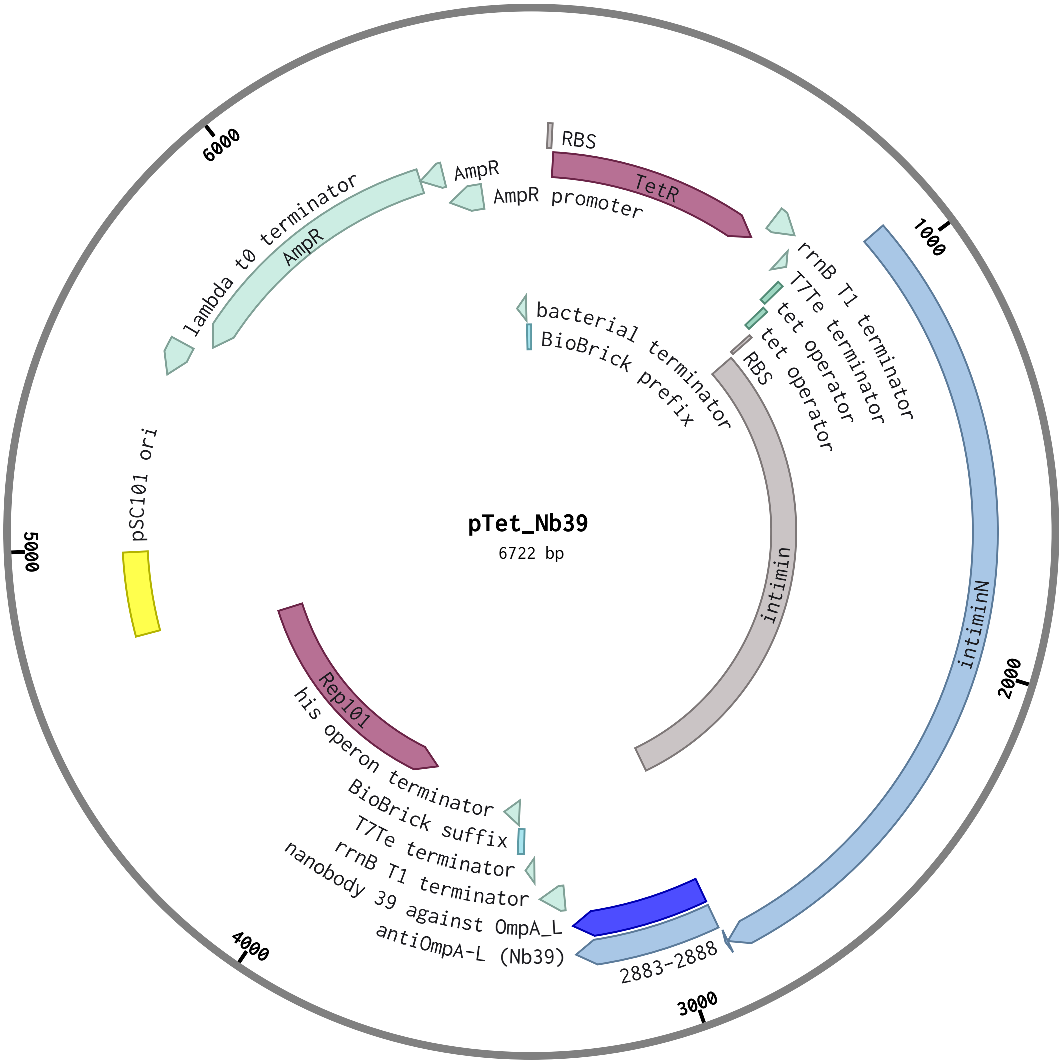

pTet_Nb39 (6722 bp)

tggtgcaaaacctttgcggtatggcatgatagcgcctactagagaaagaggagaaatactagatgtccagattagataaaagtaaagtgattaacagcgcattagag
accacgttttggaaacgccataccgtactatcgcggatgatctctttctcctctttatgatctacaggtctaatctattttcatttcactaattgtcgcgtaatctc

RBS TetR

10 20 30 40 50 60 70 80 90 100

ctgcttaatgaggtcggaatcgaaggtttaacaacccgtaaactcgcccagaagctaggtgtagagcagcctacattgtattggcatgtaaaaaataagcgggcttt
gacgaattactccagccttagcttccaaattgttgggcatttgagcgggtcttcgatccacatctcgtcggatgtaacataaccgtacattttttattcgcccgaaa

TetR

110 120 130 140 150 160 170 180 190 200 210

gctcgacgccttagccattgagatgttagataggcaccatactcacttttgccctttagaaggggaaagctggcaagattttttacgtaataacgctaaaagtttta
cgagctgcggaatcggtaactctacaatctatccgtggtatgagtgaaaacgggaaatcttcccctttcgaccgttctaaaaaatgcattattgcgattttcaaaat

TetR

220 230 240 250 260 270 280 290 300 310 320

gatgtgctttactaagtcatcgcgatggagcaaaagtacatttaggtacacggcctacagaaaaacagtatgaaactctcgaaaatcaattagccttttttatgccaa
ctacacgaaatgattcagtagcgctacctcgttttcatgtaaatccatgtgccggatgtctttttgtcatactttgagagcttttagttaatcggaaaaaatacggtt

TetR

330 340 350 360 370 380 390 400 410 420

caaggtttttcactagagaatgcattatatgcactcagcgctgtggggcattttactttaggttgcgtattggaagatcaagagcatcaagtcgctaaagaagaaag
gttccaaaaagtgatctcttacgtaatatacgtgagtcgcgacaccccgtaaaatgaaatccaacgcataaccttctagttctcgtagttcagcgatttcttctttc

TetR

430 440 450 460 470 480 490 500 510 520 530

ggaaacacctactactgatagtatgccgccattattacgacaagctatcgaattatttgatcaccaaggtgcagagccagccttcttattcggccttgaattgatca
cctttgtggatgatgactatcatacggcggtaataatgctgttcgatagcttaataaactagtggttccacgtctcggtcggaagaataagccggaacttaactagt

TetR

540 550 560 570 580 590 600 610 620 630 640

tatgcggattagaaaaacaacttaaatgtgaaagtgggtcctaataatactagagccaggcatcaaataaaacgaaaggctcagtcgaaagactgggcctttcgttt
atacgcctaatctttttgttgaatttacactttcacccaggattattatgatctcggtccgtagtttattttgctttccgagtcagctttctgacccggaaagcaaa

TetR rrnB T1 terminator

650 660 670 680 690 700 710 720 730 740

tatctgttgtttgtcggtgaacgctctctactagagtcacactggctcaccttcgggtgggcctttctgcgtttatatactagagtccctatcagtgatagagattg
atagacaacaaacagccacttgcgagagatgatctcagtgtgaccgagtggaagcccacccggaaagacgcaaatatatgatctcagggatagtcactatctctaac

rrnB T1 terminator T7Te terminator tet operator

750 760 770 780 790 800 810 820 830 840 850

```
acatccctatcagtgatagagatactgagcactactagagaaagaggagaaatactagatgattactcatggttgttatacccggacccggcacaagcataagctaa
tgtagggatagtcactatctctatgactcgtgatgatctctttctcctctttatgatctactaatgagtaccaacaatatgggcctgggccgtgttcgtattcgatt
                                                          M  I  T  H  G  C  Y  T  R  T  R  H  K  H  K  L
                                                          intiminN
   tet operator                        RBS                intimin
   860       870       880       890       900       910       920       930       940       950       960
```

```
aaaaaacattgattatgcttagtgctggtttaggattgtttttttatgttaatcagaactcatttgcaaatggtgaaaattattttaaattgggttcggattcaaaa
ttttttgtaactaatacgaatcacgaccaaatcctaacaaaaaaatacaattagtcttgagtaaacgtttaccacttttaataaaatttaacccaagcctaagtttt
K K T L I M L S A G L G L F F Y V N Q N S F A N G E N Y F K L G S D S K
intiminN
intimin
    970       980       990     1,000     1,010     1,020     1,030     1,040     1,050     1,060   1,070
```

```
ctgttaactcatgatagctatcagaatcgcctttttatacgttgaaaactggtgaaactgttgccgatctttctaaatcgcaagatattaatttatcgacgatttg
gacaattgagtactatcgatagtcttagcggaaaaaatatgcaacttttgaccactttgacaacggctagaaagatttagcgttctataattaaatagctgctaaac
L L T H D S Y Q N R L F Y T L K T G E T V A D L S K S Q D I N L S T I W
intiminN
intimin
      1,080     1,090     1,100     1,110     1,120     1,130     1,140     1,150     1,160     1,170
```

```
gtcgttgaataagcatttatacagttctgaaagcgaaatgatgaaggccgcgcctggtcagcagatcattttgccactcaaaaacttccctttgaatacagtgcac
cagcaacttattcgtaaatatgtcaagactttcgctttactacttccggcgcggaccagtcgtctagtaaaacggtgagtttttgaagggaaacttatgtcacgtg
S L N K H L Y S S E S E M M K A A P G Q Q I I L P L K K L P F E Y S A
intiminN
intimin
1,180     1,190     1,200     1,210     1,220     1,230     1,240     1,250     1,260     1,270     1,280
```

```
taccacttttaggttcggcacctcttgttgctgcgggtggtgttgctggtcacacgaataaactgactaaaatgtccccggacgtgaccaaaagcaacatgaccgat
atggtgaaaatccaagccgtggagaacaacgacgcccaccacaacgaccagtgtgcttatttgactgattttacaggggcctgcactggttttcgttgtactggcta
L P L L G S A P L V A A G G V A G H T N K L T K M S P D V T K S N M T D
intiminN
intimin
  1,290     1,300     1,310     1,320     1,330     1,340     1,350     1,360     1,370     1,380   1,390
```

```
gacaaggcattaaattatgcggcacaacaggcggcgagtctcggtagccagcttcagtcgcgatctctgaacggcgattacgcgaaagataccgctcttggtatcgc
ctgttccgtaatttaatacgccgtgttgtccgccgctcagagccatcggtcgaagtcagcgctagagacttgccgctaatgcgctttctatggcgagaaccatagcg
D K A L N Y A A Q Q A A S L G S Q L Q S R S L N G D Y A K D T A L G I A
intiminN
intimin
      1,400     1,410     1,420     1,430     1,440     1,450     1,460     1,470     1,480     1,490
```

```
tggtaaccaggcttcgtcacagttgcaggcctggttacaacattatggaacggcagaggttaatctgcaaagtggtaataactttgacggtagttcactggacttct
accattggtccgaagcagtgtcaacgtccggaccaatgttgtaataccttgccgtctccaattagacgtttcaccattattgaaactgccatcaagtgacctgaaga
 G  N  Q  A  S  S  Q  L  Q  A  W  L  Q  H  Y  G  T  A  E  V  N  L  Q  S  G  N  N  F  D  G  S  S  L  D  F
intiminN
intimin
1,500 1,510 1,520 1,530 1,540 1,550 1,560 1,570 1,580 1,590 1,600

tattaccgttctatgattccgaaaaaatgctggcatttggtcaggtcggagcgcgttacattgactcccgctttacggcaaatttaggtgcgggtcagcgtttttc
ataatggcaagatactaaggcttttttacgaccgtaaaccagtccagcctcgcgcaatgtaactgagggcgaaatgccgtttaaatccacgcccagtcgcaaaaag
L L  P  F  Y  D  S  E  K  M  L  A  F  G  Q  V  G  A  R  Y  I  D  S  R  F  T  A  N  L  G  A  G  Q  R  F  F
intiminN
intimin
1,610 1,620 1,630 1,640 1,650 1,660 1,670 1,680 1,690 1,700 1,710

cttcctgcaaacatgttgggctataacgtcttcattgatcaggatttttctggtgataatacccgtttaggtattggtggcgaatactggcgagactatttcaaaag
gaaggacgtttgtacaacccgatattgcagaagtaactagtcctaaaaagaccactattatgggcaaatccataaccaccgcttatgaccgctctgataaagttttc
L  P  A  N  M  L  G  Y  N  V  F  I  D  Q  D  F  S  G  D  N  T  R  L  G  I  G  G  E  Y  W  R  D  Y  F  K  S
intiminN
intimin
1,720 1,730 1,740 1,750 1,760 1,770 1,780 1,790 1,800 1,810

tagcgttaacggctatttccgcatgagcggctggcatgagtcatacaataagaaagactatgatgagcgcccagcaaatggcttcgatatccgttttaatggctatc
atcgcaattgccgataaaggcgtactcgccgaccgtactcagtatgttattctttctgatactactcgcgggtcgtttaccgaagctataggcaaaattaccgatag
 S  V  N  G  Y  F  R  M  S  G  W  H  E  S  Y  N  K  K  D  Y  D  E  R  P  A  N  G  F  D  I  R  F  N  G  Y
intiminN
intimin
1,820 1,830 1,840 1,850 1,860 1,870 1,880 1,890 1,900 1,910 1,920

taccgtcatatccggcattaggcgccaagctgatatatgagcagtattatggtgataatgttgctttgtttaattctgataagctgcaatcgaatcctggtgcggcg
atggcagtataggccgtaatccgcggttcgactatatactcgtcataataccactattacaacgaaacaaattaagactattcgacgttagcttaggaccacgccgc
L P  S  Y  P  A  L  G  A  K  L  I  Y  E  Q  Y  Y  G  D  N  V  A  L  F  N  S  D  K  L  Q  S  N  P  G  A  A
intiminN
intimin
1,930 1,940 1,950 1,960 1,970 1,980 1,990 2,000 2,010 2,020 2,030

accgttggtgtaaactatactccgattcctctggtgacgatggggatcgattaccgtcatggtacgggtaatgaaaatgatctcctttactcaatgcagttccgtta
tggcaaccacatttgatatgaggctaaggagaccactgctacccctagctaatggcagtaccatgcccattactttactagaggaaatgagttacgtcaaggcaat
T  V  G  V  N  Y  T  P  I  P  L  V  T  M  G  I  D  Y  R  H  G  T  G  N  E  N  D  L  L  Y  S  M  Q  F  R  Y
intiminN
intimin
2,040 2,050 2,060 2,070 2,080 2,090 2,100 2,110 2,120 2,130 2,140
```

tcagtttgataaatcgtggtctcagcaaattgaaccacagtatgttaacgagttaagaacattatcaggcagccgttacgatctggttcagcgtaataacaatatta
agtcaaactatttagcaccagagtcgtttaacttggtgtcatacaattgctcaattcttgtaatagtccgtcggcaatgctagaccaagtcgcattattgttataat
Q F D K S W S Q Q I E P Q Y V N E L R T L S G S R Y D L V Q R N N N I
intiminN
intimin
2,150 2,160 2,170 2,180 2,190 2,200 2,210 2,220 2,230 2,240

ttctggagtacaagaagcaggatattctttctctgaatattccgcatgatattaatggtactgaacacagtacgcagaagattcagttgatcgttaagagcaaatac
aagacctcatgttcttcgtcctataagaaagagacttataaggcgtactataattaccatgacttgtgtcatgcgtcttctaagtcaactagcaattctcgtttatg
I L E Y K K Q D I L S L N I P H D I N G T E H S T Q K I Q L I V K S K Y
intiminN
intimin
2,250 2,260 2,270 2,280 2,290 2,300 2,310 2,320 2,330 2,340 2,350

ggtctggatcgtatcgtctgggatgatagtgcattacgcagtcagggcggtcagattcagcatagcggaagccaaagcgcacaagactaccaggctattttgcctgc
ccagacctagcatagcagaccctactatcacgtaatgcgtcagtcccgccagtctaagtcgtatcgccttcggtttcgcgtgttctgatggtccgataaaacggacg
G L D R I V W D D S A L R S Q G G Q I Q H S G S Q S A Q D Y Q A I L P A
intiminN
intimin
2,360 2,370 2,380 2,390 2,400 2,410 2,420 2,430 2,440 2,450 2,460

ttatgtgcaaggtggcagcaatatttataaagtgacggctcgcgcctatgaccgtaatggcaatagctctaacaatgtacagcttactattaccgttctgtcgaatg
aatacacgttccaccgtcgttataaatatttcactgccgagcgcggatactggcattaccgttatcgagattgttacatgtcgaatgataatggcaagacagcttac
Y V Q G G S N I Y K V T A R A Y D R N G N S S N N V Q L T I T V L S N
intiminN
intimin
2,470 2,480 2,490 2,500 2,510 2,520 2,530 2,540 2,550 2,560

gtcaagttgtcgaccaggttggggtaacggactttacggcggataagacttcggctaaagcggataacgccgataccattacttataccgcgacggtgaaaaagaat
cagttcaacagctggtccaaccccattgcctgaaatgccgcctattctgaagccgatttcgcctattgcggctatggtaatgaatatggcgctgccactttttctta
G Q V V D Q V G V T D F T A D K T S A K A D N A D T I T Y T A T V K K N
intiminN
intimin
2,570 2,580 2,590 2,600 2,610 2,620 2,630 2,640 2,650 2,660 2,670

ggggtagctcaggctaatgtccctgtttcatttaatattgtttcaggaactgcaactcttggggcaaatagtgccaaaacggatgctaacggtaaggcaaccgtaac
ccccatcgagtccgattacagggacaaagtaaattataacaaagtccttgacgttgagaaccccgtttatcacggttttgcctacgattgccattccgttggcattg
G V A Q A N V P V S F N I V S G T A T L G A N S A K T D A N G K A T V T
intiminN
intimin
2,680 2,690 2,700 2,710 2,720 2,730 2,740 2,750 2,760 2,770 2,780

gttgaagtcgagtacgccaggacaggtcgtcgtgtctgctaaaaccgcggagatgacttcagcacttaatgccagtgcggttatatttttgatggtgcgactagaT
caacttcagctcatgcggtcctgtccagcagcacagacgattttggcgcctctactgaagtcgtgaattacggtcacgccaatataaaaactaccacgctgatctA
L K S S T P G Q V V V S A K T A E M T S A L N A S A V I F F D G A T R
intiminN
intimin
2,790 2,800 2,810 2,820 2,830 2,840 2,850 2,860 2,870 2,880

CGCAGCGTCAACTGGTTGAATCTGGCGGGGGTCTGGTCCATACTGGTGGCTCATTAAAACTTTCGTGCGTCCCGAATGGTAGCATCTTCAACTTCAACCCGATGGGT
GCGTCGCAGTTGACCAACTTAGACCGCCCCCAGACCAGGTATGACCACCGAGTAATTTTGAAAGCACGCAGGGCTTACCATCGTAGAAGTTGAAGTTGGGCTACCCA
S Q R Q L V E S G G G L V H T G G S L K L S C V P N G S I F N F N P M G
antiOmpA-L (Nb39)
nanobody 39 against OmpA_L
2,890 2,900 2,910 2,920 2,930 2,940 2,950 2,960 2,970 2,980 2,990

TGGTATCGTCAAGTTTCGGGCCAACAGCGTGAACTTGTCGCTACCCTGACGCGTGACGGTGTGGAAAACTATGCGTCCTCGGTGAAAGGCCGGTTTACGATTAGTCG
ACCATAGCAGTTCAAAGCCCGGTTGTCGCACTTGAACAGCGATGGGACTGCGCACTGCCACACCTTTTGATACGCAGGAGCCACTTTCCGGCCAAATGCTAATCAGC
W Y R Q V S G Q Q R E L V A T L T R D G V E N Y A S S V K G R F T I S R
antiOmpA-L (Nb39)
nanobody 39 against OmpA_L
3,000 3,010 3,020 3,030 3,040 3,050 3,060 3,070 3,080 3,090 3,100

TGATTCCGCGAAAAATACTCTCTATCTGCAGATGACGGATGTTAAACCGGGTGACGCGGCGGTCTATATCTGTCACGCAAATTATCGTATTGGCCGTAATGACCTTC
ACTAAGGCGCTTTTTATGAGAGATAGACGTCTACTGCCTACAATTTGGCCCACTGCGCCGCCAGATATAGACAGTGCGTTTAATAGCATAACCGGCATTACTGGAAG
D S A K N T L Y L Q M T D V K P G D A A V Y I C H A N Y R I G R N D L
antiOmpA-L (Nb39)
nanobody 39 against OmpA_L
3,110 3,120 3,130 3,140 3,150 3,160 3,170 3,180 3,190 3,200 3,210

CTGTGTGGGGCAAAGGGACACCAGTTACGGTCAGCTAAtactagagccaggcatcaaataaaacgaaaggctcagtcgaaagactgggcctttcgttttatctgttg
GACACACCCCGTTTCCCTGTGGTCAATGCCAGTCGATTatgatctcggtccgtagtttattttgctttccgagtcagctttctgacccggaaagcaaaatagacaac
P V W G K G T P V T V S *
antiOmpA-L (Nb39)
nanobody 39 against OmpA_L
rrnB T1 terminator
3,220 3,230 3,240 3,250 3,260 3,270 3,280 3,290 3,300 3,310

tttgtcggtgaacgctctctactagagtcacactggctcaccttcgggtgggcctttctgcgtttatatactagtagcggccgctgcagtccggcaaaaaagggcaa
aaacagccacttgcgagagatgatctcagtgtgaccgagtggaagcccacccggaaagacgcaaatatatgatcatcgccggcgacgtcaggccgtttttcccgtt
rrnB T...nator
T7Te terminator
BioBrick suffix
his op...nator
3,320 3,330 3,340 3,350 3,360 3,370 3,380 3,390 3,400 3,410 3,420

ggtgtcaccaccctgcccttttctttaaaaccgaaaagattacttcgcgttatgcaggcttcctcgctcactgactcgctgcgctcggtcgttcggctgcggcgag
ccacagtggtgggacgggaaaagaaattttggcttttctaatgaagcgcaatacgtccgaaggagcgagtgactgagcgacgcgagccagcaagccgacgccgctc

his operon terminator

3,430 3,440 3,450 3,460 3,470 3,480 3,490 3,500 3,510 3,520 3,530

cggtatcagctcactcaaaggcggtaatctcgaggttacattgtcgatctgttcatggtgaacagctttgaatgcaccaaaaactcgtaaaagctctgatgtatcta
gccatagtcgagtgagtttccgccattagagctccaatgtaacagctagacaagtaccacttgtcgaaacttacgtggtttttgagcattttcgagactacatagat

3,540 3,550 3,560 3,570 3,580 3,590 3,600 3,610 3,620 3,630

tctttttacaccgttttcatctgtgcatatggacagttttccctttgatatgtaacggtgaacagttgttctactttgtttgttagtcttgatgcttcactgata
agaaaaaatgtggcaaaagtagacacgtatacctgtcaaaagggaaactatacattgccacttgtcaacaagatgaaaacaaacaatcagaactacgaagtgactat

3,640 3,650 3,660 3,670 3,680 3,690 3,700 3,710 3,720 3,730 3,740

gatacaagagccataagaacctcagatccttccgtatttagccagtatgttctctagtgtggttcgttgtttttgcgtgagccatgagaacgaaccattgagatcat
ctatgttctcggtattcttggagtctaggaaggcataaatcggtcatacaagagatcacaccaagcaacaaaaacgcactcggtactcttgcttggtaactctagta

Rep101

3,750 3,760 3,770 3,780 3,790 3,800 3,810 3,820 3,830 3,840 3,850

acttactttgcatgtcactcaaaaatttgcctcaaaactggtgagctgaatttttgcagttaaagcatcgtgtagtgtttttcttagtccgttatgtaggtaggaa
tgaatgaaacgtacagtgagtttttaaacggagttttgaccactcgacttaaaaacgtcaatttcgtagcacatcacaaaaagaatcaggcaatacatccatcctt

Rep101

3,860 3,870 3,880 3,890 3,900 3,910 3,920 3,930 3,940 3,950

tctgatgtaatggttgttggtattttgtcaccattcatttttatctggttgttctcaagttcggttacgagatccatttgtctatctagttcaacttggaaaatcaa
agactacattaccaacaaccataaaacagtggtaagtaaaaatagaccaacaagagttcaagccaatgctctaggtaaacagatagatcaagttgaaccttttagtt

Rep101

3,960 3,970 3,980 3,990 4,000 4,010 4,020 4,030 4,040 4,050 4,060

cgtatcagtcgggcggcctcgcttatcaaccaccaatttcatattgctgtaagtgtttaaatctttacttattggtttcaaaacccattggttaagccttttaaact
gcatagtcagcccgccggagcgaatagttggtggttaaagtataacgacattcacaaatttagaaatgaataaccaaagttttgggtaaccaattcggaaaatttga

Rep101

4,070 4,080 4,090 4,100 4,110 4,120 4,130 4,140 4,150 4,160 4,170

catggtagttattttcaagcattaacatgaacttaaattcatcaaggctaatctctatatttgccttgtgagttttcttttgtgttagttcttttaataaccactca
gtaccatcaataaaagttcgtaattgtacttgaatttaagtagttccgattagagatataaacggaacactcaaaagaaaacacaatcaagaaaattattggtgagt

Rep101

4,180 4,190 4,200 4,210 4,220 4,230 4,240 4,250 4,260 4,270 4,280

taaatcctcatagagtatttgttttcaaaagacttaacatgttccagattatattttatgaatttttttaactggaaaagataaggcaatatctcttcactaaaaac
atttaggagtatctcataaacaaaagttttctgaattgtacaaggtctaatataaaatacttaaaaaaattgaccttttctattccgttatagagaagtgatttttg

Rep101

4,290 4,300 4,310 4,320 4,330 4,340 4,350 4,360 4,370 4,380

taattctaatttttcgcttgagaacttggcatagtttgtccactggaaaatctcaaagcctttaaccaaaggattcctgatttccacagttctcgtcatcagctctc
attaagattaaaaagcgaactcttgaaccgtatcaaacaggtgaccttttagagtttcggaaattggtttcctaaggactaaaggtgtcaagagcagtagtcgagag

Rep101

4,390 4,400 4,410 4,420 4,430 4,440 4,450 4,460 4,470 4,480 4,490

tggttgctttagctaatacaccataagcattttccctactgatgttcatcatctgagcgtattggttataagtgaacgataccgtccgttctttccttgtagggttt
accaacgaaatcgattatgtggtattcgtaaaagggatgactacaagtagtagactcgcataaccaatattcacttgctatggcaggcaagaaaggaacatcccaaa

Rep101

4,500 4,510 4,520 4,530 4,540 4,550 4,560 4,570 4,580 4,590 4,600

tcaatcgtggggttgagtagtgccacacagcataaaattagcttggtttcatgctccgttaagtcatagcgactaatcgctagttcatttgctttgaaaacaactaa
agttagcaccccaactcatcacggtgtgtcgtattttaatcgaaccaaagtacgaggcaattcagtatcgctgattagcgatcaagtaaacgaaacttttgttgatt

Rep101

4,610 4,620 4,630 4,640 4,650 4,660 4,670 4,680 4,690 4,700

ttcagacatacatctcaattggtctaggtgattttaatcactataccaattgagatgggctagtcaatgataattacatgtccttttcctttgagttgtgggtatct
aagtctgtatgtagagttaaccagatccactaaaattagtgatatggttaactctacccgatcagttactattaatgtacaggaaaaggaaactcaacacccataga

pSC101 ori

4,710 4,720 4,730 4,740 4,750 4,760 4,770 4,780 4,790 4,800 4,810

gtaaattctgctagacctttgctggaaaacttgtaaattctgctagaccctctgtaaattccgctagacctttgtgtgttttttttgtttatattcaagtggttata
catttaagacgatctggaaacgaccttttgaacatttaagacgatctgggagacatttaaggcgatctggaaacacacaaaaaaaacaaatataagttcaccaatat

pSC101 ori

4,820 4,830 4,840 4,850 4,860 4,870 4,880 4,890 4,900 4,910 4,920

atttatagaataaagaaagaataaaaaaagataaaaagaatagatcccagccctgtgtataactcactactttagtcagttccgcagtattacaaaaggatgtcgca
taaatatcttatttctttcttatttttttctatttttcttatctagggtcgggacacatattgagtgatgaaatcagtcaaggcgtcataatgttttcctacagcgt

pSC101 ori

4,930 4,940 4,950 4,960 4,970 4,980 4,990 5,000 5,010 5,020

aacgctgtttgctcctctacaaaacagaccttaaaaccctaaaggcttaagtagcaccctcgcaagctcgggcaaatcgctgaatattccttttgtctccgaccatc
ttgcgacaaacgaggagatgttttgtctggaattttgggatttccgaattcatcgtgggagcgttcgagcccgtttagcgacttataaggaaaacagaggctggtag

5,030 5,040 5,050 5,060 5,070 5,080 5,090 5,100 5,110 5,120 5,130

aggcacctgagtcgctgtctttttcgtgacattcagttcgctgcgctcacggctctggcagtgaatgggggtaaatggcactacaggcgccttttatggattcatgc
tccgtggactcagcgacagaaaaagcactgtaagtcaagcgacgcgagtgccgagaccgtcacttacccccatttaccgtgatgtccgcggaaaatacctaagtacg

5,140 5,150 5,160 5,170 5,180 5,190 5,200 5,210 5,220 5,230 5,240

aaggaaactacccataatacaagaaaagcccgtcacgggcttctcagggcgttttatggcgggtctgctatgtggtgctatctgactttttgctgttcagcagttcc
ttcctttgatgggtattatgttcttttcgggcagtgcccgaagagtcccgcaaaataccgcccagacgatacaccacgatagactgaaaaacgacaagtcgtcaagg

5,250 5,260 5,270 5,280 5,290 5,300 5,310 5,320 5,330 5,340 5,350

tgccctctgattttccagtctgaccacttcggattatcccgtgacaggtcattcagactggctaatgcacccagtaaggcagcggtatcatcaacaggcttacccgt
acgggagactaaaaggtcagactggtgaagcctaatagggcactgtccagtaagtctgaccgattacgtgggtcattccgtcgccatagtagttgtccgaatgggca

5,360 5,370 5,380 5,390 5,400 5,410 5,420 5,430 5,440 5,450

cttactgtccctagtgcttggattctcaccaataaaaaacgcccggcggcaaccgagcgttctgaacaaatccagatggagttctgaggtcattactggatctatca
gaatgacagggatcacgaacctaagagtggttattttttgcgggccgccgttggctcgcaagacttgtttaggtctacctcaagactccagtaatgacctagatagt

lambda t0 terminator

5,460 5,470 5,480 5,490 5,500 5,510 5,520 5,530 5,540 5,550 5,560

acaggagtccaagcgagctcgtaaacttggtctgacagttaccaatgcttaatcagtgaggcacctatctcagcgatctgtctatttcgttcatccatagttgcctg
tgtcctcaggttcgctcgagcatttgaaccagactgtcaatggttacgaattagtcactccgtggatagagtcgctagacagataaagcaagtaggtatcaacggac

AmpR

5,570 5,580 5,590 5,600 5,610 5,620 5,630 5,640 5,650 5,660 5,670

actccccgtcgtgtagataactacgatacgggagggcttaccatctggccccagtgctgcaatgataccgcgagacccacgctcaccggctccagatttatcagcaa
tgaggggcagcacatctattgatgctatgccctcccgaatggtagaccggggtcacgacgttactatggcgctctgggtgcgagtggccgaggtctaaatagtcgtt

AmpR

5,680 5,690 5,700 5,710 5,720 5,730 5,740 5,750 5,760 5,770

taaaccagccagccggaagggccgagcgcagaagtggtcctgcaactttatccgcctccatccagtctattaattgttgccgggaagctagagtaagtagttcgcca
atttggtcggtcggccttcccggctcgcgtcttcaccaggacgttgaaataggcggaggtaggtcagataattaacaacggcccttcgatctcattcatcaagcggt

AmpR

5,780 5,790 5,800 5,810 5,820 5,830 5,840 5,850 5,860 5,870 5,880

gttaatagtttgcgcaacgttgttgccattgctacaggcatcgtggtgtcacgctcgtcgtttggtatggcttcattcagctccggttcccaacgatcaaggcgagt
caattatcaaacgcgttgcaacaacggtaacgatgtccgtagcaccacagtgcgagcagcaaaccataccgaagtaagtcgaggccaagggttgctagttccgctca

AmpR

5,890 5,900 5,910 5,920 5,930 5,940 5,950 5,960 5,970 5,980 5,990

tacatgatcccccatgttgtgcaaaaaagcggttagctccttcggtcctccgatcgttgtcagaagtaagttggccgcagtgttatcactcatggttatggcagcac
atgtactagggggtacaacacgttttttcgccaatcgaggaagccaggaggctagcaacagtcttcattcaaccggcgtcacaatagtgagtaccaataccgtcgtg

AmpR

6,000 6,010 6,020 6,030 6,040 6,050 6,060 6,070 6,080 6,090

tgcataattctcttactgtcatgccatccgtaagatgcttttctgtgactggtgagtactcaaccaagtcattctgagaatagtgtatgcggcgaccgagttgctct
acgtattaagagaatgacagtacggtaggcattctacgaaaagacactgaccactcatgagttggttcagtaagactcttatcacatacgccgctggctcaacgaga

AmpR

6,100 6,110 6,120 6,130 6,140 6,150 6,160 6,170 6,180 6,190 6,200

tgcccggcgtcaatacgggataataccgcgccacatagcagaactttaaaagtgctcatcattggaaaacgttcttcggggcgaaaactctcaaggatcttaccgct
acgggccgcagttatgccctattatggcgcggtgtatcgtcttgaaattttcacgagtagtaaccttttgcaagaagccccgcttttgagagttcctagaatggcga

AmpR

6,210 6,220 6,230 6,240 6,250 6,260 6,270 6,280 6,290 6,300 6,310

gttgagatccagttcgatgtaacccactcgtgcacccaactgatcttcagcatcttttactttcaccagcgtttctgggtgagcaaaaacaggaaggcaaaatgccg
caactctaggtcaagctacattgggtgagcacgtgggttgactagaagtcgtagaaaatgaaagtggtcgcaaagacccactcgtttttgtccttccgttttacggc

AmpR AmpR

6,320 6,330 6,340 6,350 6,360 6,370 6,380 6,390 6,400 6,410 6,420

caaaaaagggaataagggcgacacggaaatgttgaatactcatactcttcctttttcaatattattgaagcatttatcagggttattgtctcatgagcggatacata
gtttttcccttattcccgctgtgcctttacaacttatgagtatgagaaggaaaaagttataataacttcgtaaatagtcccaataacagagtactcgcctatgtat

AmpR AmpR promoter

6,430 6,440 6,450 6,460 6,470 6,480 6,490 6,500 6,510 6,520

tttgaatgtatttagaaaaataaacaaataggggttccgcgcacatttccccatggtgccacctgacgtctaagaaaccattattatcatgacattaacctataaaaa
aaacttacataaatctttttatttgtttatccccaaggcgcgtgtaaagggtaccacggtggactgcagattctttggtaataatagtactgtaattggatattttt

AmpR promoter

6,530 6,540 6,550 6,560 6,570 6,580 6,590 6,600 6,610 6,620 6,630

taggcgtatcacgaggcagaatttcagataaaaaaaatccttagctttcgctaaggatgatttctggaattcgcggccgcttctagag
atccgcatagtgctccgtcttaaagtctattttttttaggaatcgaaagcgattcctactaaagaccttaagcgccggcgaagatctc

bacterial terminator BioBrick prefix

6,640 6,650 6,660 6,670 6,680 6,690 6,700 6,710 6,720
