# Supplementary material for: Reprogrammed SimCells for antimicrobial therapy
Source: Proc Natl Acad Sci U S A. 2026 Mar 17;123(12):e2517118123. doi: 10.1073/pnas.2517118123 (PMC13012131; doi:10.1073/pnas.2517118123)
Supplement: Supplementary file 6 — Dataset S05 (PDF) [file pnas.2517118123.sd05.pdf]

pNb39_NahG (8042 bp)

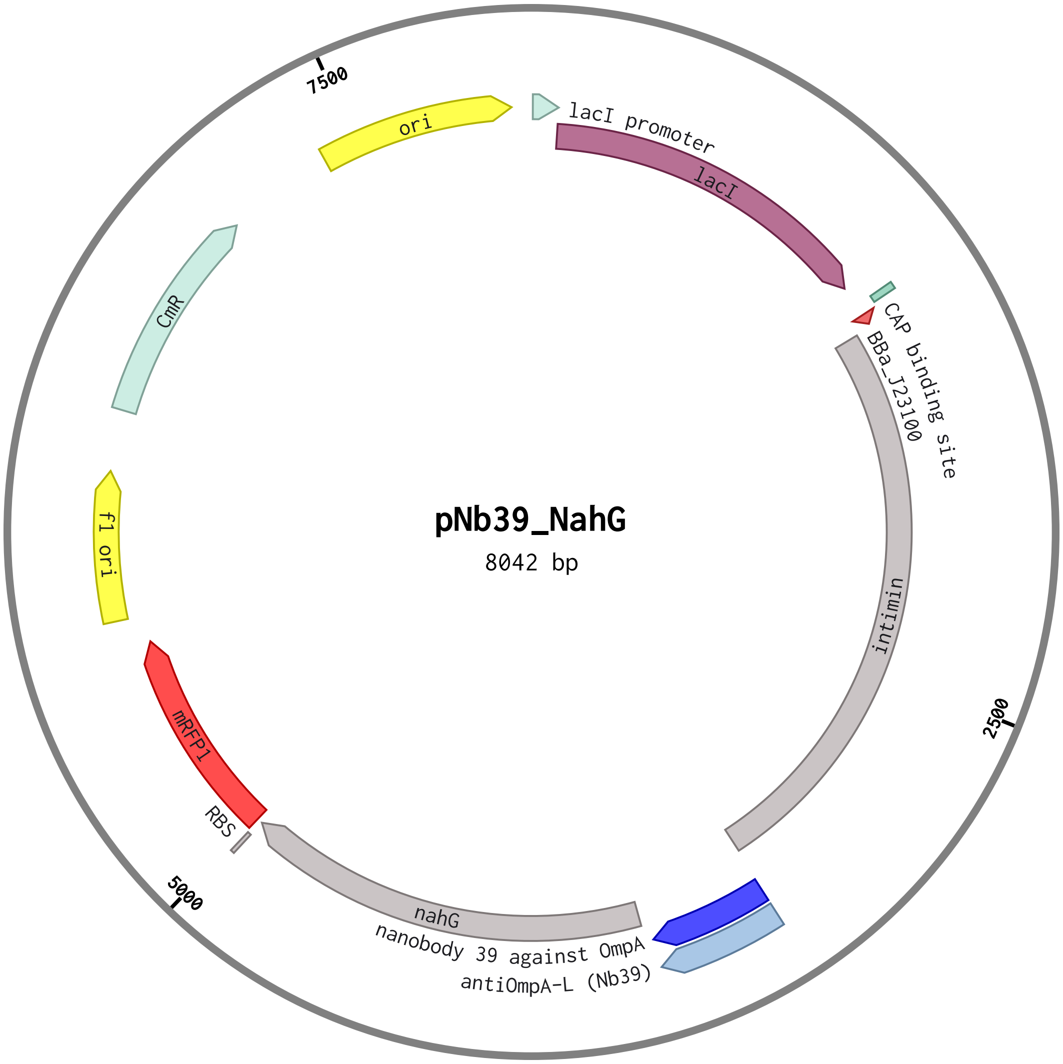

pNb39_NahG (8042 bp)

acccgacaccatcgaatggcgcaaaacctttcgcggtatggcatgatagcgcccggaagagagtcaattcagggtggtgaatgtgaaaccagtaacgttatacgatg
tgggctgtggtagcttaccgcgttttggaaagcgccataccgtactatcgcgggccttctctcagttaagtcccaccacttacactttggtcattgcaatatgctac

lacI promoter lacI

10 20 30 40 50 60 70 80 90 100

tcgcagagtatgccggtgtctcttatcagaccgtttcccgcgtggtgaaccaggccagccacgtttctgcgaaaacgcgggaaaaagtggaagcggcgatggcggag
agcgtctcatacggccacagagaatagtctggcaaagggcgcaccacttggtccggtcggtgcaaagacgcttttgcgccctttttcaccttcgccgctaccgcctc

lacI

110 120 130 140 150 160 170 180 190 200 210

ctgaattacattcccaaccgcgtggcacaacaactggcgggcaaacagtcgttgctgattggcgttgccacctccagtctggccctgcacgcgccgtcgcaaattgt
gacttaatgtaagggttggcgcaccgtgttgttgaccgcccgtttgtcagcaacgactaaccgcaacggtggaggtcagaccgggacgtgcgcggcagcgtttaaca

lacI

220 230 240 250 260 270 280 290 300 310 320

cgcggcgattaaatctcgcgccgatcaactgggtgccagcgtggtggtgtcgatggtagaacgaagcggcgtcgaagcctgtaaagcggcggtgcacaatcttctcg
gcgccgctaatttagagcgcggctagttgacccacggtcgcaccaccacagctaccatcttgcttcgccgcagcttcggacatttcgccgccacgtgttagaagagc

lacI

330 340 350 360 370 380 390 400 410 420

cgcaacgcgtcagtgggctgatcattaactatccgctggatgaccaggatgccattgctgtggaagctgcctgcactaatgttccggcgttatttcttgatgtctct
gcgttgcgcagtcacccgactagtaattgataggcgacctactggtcctacggtaacgacaccttcgacggacgtgattacaaggccgcaataaagaactacagaga

lacI

430 440 450 460 470 480 490 500 510 520 530

gaccagacacccatcaacagtattattttctcccatgaagacggtacgcgactgggcgtggagcatctggtcgcattgggtcaccagcaaatcgcgctgttagcggg
ctggtctgtgggtagttgtcataataaaagagggtacttctgccatgcgctgacccgcacctcgtagaccagcgtaacccagtggtcgtttagcgcgacaatcgccc

lacI

540 550 560 570 580 590 600 610 620 630 640

cccattaagttctgtctcggcgcgtctgcgtctggctggctggcataaatatctcactcgcaatcaaattcagccgatagcggaacgggaaggcgactggagtgcca
gggtaattcaagacagagccgcgcagacgcagaccgaccgaccgtatttatagagtgagcgttagtttaagtcggctatcgccttgcccttccgctgacctcacggt

lacI

650 660 670 680 690 700 710 720 730 740

tgtccggttttcaacaaaccatgcaaatgctgaatgagggcatcgttcccactgcgatgctggttgccaacgatcagatggcgctgggcgcaatgcgcgccattacc
acaggccaaaagttgtttggtacgtttacgacttactcccgtagcaagggtgacgctacgaccaacggttgctagtctaccgcgacccgcgttacgcgcggtaatgg

lacI

750 760 770 780 790 800 810 820 830 840 850

gagtccgggctgcgcgttggtgcggacatctcggtagtgggatacgacgataccgaagacagctcatgttatatcccgccgttaaccaccatcaaacaggattttcg
ctcaggcccgacgcgcaaccacgcctgtagagccatcaccctatgctgctatggcttctgtcgagtacaatatagggcggcaattggtggtagtttgtcctaaaagc

lacI

860 870 880 890 900 910 920 930 940 950 960

cctgctggggcaaaccagcgtggaccgcttgctgcaactctctcagggccaggcggtgaagggcaatcagctgttgcccgtctcactggtgaaaagaaaaaccaccc
ggacgaccccgtttggtcgcacctggcgaacgacgttgagagagtcccggtccgccacttcccgttagtcgacaacgggcagagtgaccacttttcttttttggtggg

lacI

970 980 990 1,000 1,010 1,020 1,030 1,040 1,050 1,060 1,070

tggcgcccaatacgcaaaccgcctctccccgcgcgttggccgattcattaatgcagctggcacgacaggtttcccgactggaaagcgggcagtgagcggtacccgat
accgcgggttatgcgtttggcggagaggggcgcgcaaccggctaagtaattacgtcgaccgtgctgtccaaagggctgacctttcgcccgtcactcgccatgggcta

lacI

1,080 1,090 1,100 1,110 1,120 1,130 1,140 1,150 1,160 1,170

aaaagcggcttcctgacaggaggccgttttgttttgcagcccacctcaacgcaattaatgtgagttagctcactcattaggcaccccaggcttgacggctagctcag
ttttcgccgaaggactgtcctccggcaaaacaaaacgtcgggtggagttgcgttaattacactcaatcgagtgagtaatccgtggggtccgaactgccgatcgagtc

CAP binding site BBa_J23100

1,180 1,190 1,200 1,210 1,220 1,230 1,240 1,250 1,260 1,270 1,280

tcctaggtacagtgctagcAAAGGAAATCTAATGATTACTCATGGTTGTTATACCCGGACCCGGCACAAGCATAAGCTAAAAAAAACATTGATTATGCTTAGTGCTG
aggatccatgtcacgatcgTTTCCTTTAGATTACTAATGAGTACCAACAATATGGGCCTGGGCCGTGTTCGTATTCGATTTTTTTTGTAACTAATACGAATCACGAC

BBa_J23100 intimin

1,290 1,300 1,310 1,320 1,330 1,340 1,350 1,360 1,370 1,380 1,390

GTTTAGGATTGTTTTTTTATGTTAATCAGAACTCATTTGCAAATGGTGAAAATTATTTTAAATTGGGTTCGGATTCAAAACTGTTAACTCATGATAGCTATCAGAAT
CAAATCCTAACAAAAAAATACAATTAGTCTTGAGTAAACGTTTACCACTTTTAATAAAATTTAACCCAAGCCTAAGTTTTGACAATTGAGTACTATCGATAGTCTTA

intimin

1,400 1,410 1,420 1,430 1,440 1,450 1,460 1,470 1,480 1,490

CGCCTTTTTTATACGTTGAAAACTGGTGAAACTGTTGCCGATCTTTCTAAATCGCAAGATATTAATTTATCGACGATTTGGTCGTTGAATAAGCATTTATACAGTTC
GCGGAAAAAATATGCAACTTTTGACCACTTTGACAACGGCTAGAAAGATTTAGCGTTCTATAATTAAATAGCTGCTAAACCAGCAACTTATTCGTAAATATGTCAAG

intimin

1,500 1,510 1,520 1,530 1,540 1,550 1,560 1,570 1,580 1,590 1,600

TGAAAGCGAAATGATGAAGGCCGCGCCTGGTCAGCAGATCATTTTGCCACTCAAAAAACTTCCCTTTGAATACAGTGCACTACCACTTTTAGGTTCGGCACCTCTTG
ACTTTCGCTTTACTACTTCCGGCGCGGACCAGTCGTCTAGTAAAACGGTGAGTTTTTTGAAGGGAAACTTATGTCACGTGATGGTGAAAATCCAAGCCGTGGAGAAC

intimin

1,610 1,620 1,630 1,640 1,650 1,660 1,670 1,680 1,690 1,700 1,710

TTGCTGCGGGTGGTGTTGCTGGTCACACGAATAAACTGACTAAAATGTCCCCGGACGTGACCAAAAGCAACATGACCGATGACAAGGCATTAAATTATGCGGCACAA
AACGACGCCCACCACAACGACCAGTGTGCTTATTTGACTGATTTTACAGGGGCCTGCACTGGTTTTCGTTGTACTGGCTACTGTTCCGTAATTTAATACGCCGTGTT

intimin

1,720 1,730 1,740 1,750 1,760 1,770 1,780 1,790 1,800 1,810

CAGGCGGCGAGTCTCGGTAGCCAGCTTCAGTCGCGATCTCTGAACGGCGATTACGCGAAAGATACCGCTCTTGGTATCGCTGGTAACCAGGCTTCGTCACAGTTGCA
GTCCGCCGCTCAGAGCCATCGGTCGAAGTCAGCGCTAGAGACTTGCCGCTAATGCGCTTTCTATGGCGAGAACCATAGCGACCATTGGTCCGAAGCAGTGTCAACGT

intimin

1,820 1,830 1,840 1,850 1,860 1,870 1,880 1,890 1,900 1,910 1,920

GGCCTGGTTACAACATTATGGAACGGCAGAGGTTAATCTGCAAAGTGGTAATAACTTTGACGGTAGTTCACTGGACTTCTTATTACCGTTCTATGATTCCGAAAAAA
CCGGACCAATGTTGTAATACCTTGCCGTCTCCAATTAGACGTTTCACCATTATTGAAACTGCCATCAAGTGACCTGAAGAATAATGGCAAGATACTAAGGCTTTTTT

intimin

1,930 1,940 1,950 1,960 1,970 1,980 1,990 2,000 2,010 2,020 2,030

TGCTGGCATTTGGTCAGGTCGGAGCGCGTTACATTGACTCCCGCTTTACGGCAAATTTAGGTGCGGGTCAGCGTTTTTTCCTTCCTGCAAACATGTTGGGCTATAAC
ACGACCGTAAACCAGTCCAGCCTCGCGCAATGTAACTGAGGGCGAAATGCCGTTTAAATCCACGCCCAGTCGCAAAAAAGGAAGGACGTTTGTACAACCCGATATTG

intimin

2,040 2,050 2,060 2,070 2,080 2,090 2,100 2,110 2,120 2,130 2,140

GTCTTCATTGATCAGGATTTTTCTGGTGATAATACCCGTTTAGGTATTGGTGGCGAATACTGGCGAGACTATTTCAAAAGTAGCGTTAACGGCTATTTCCGCATGAG
CAGAAGTAACTAGTCCTAAAAAGACCACTATTATGGGCAAATCCATAACCACCGCTTATGACCGCTCTGATAAAGTTTTCATCGCAATTGCCGATAAAGGCGTACTC

intimin

2,150 2,160 2,170 2,180 2,190 2,200 2,210 2,220 2,230 2,240

CGGCTGGCATGAGTCATACAATAAGAAAGACTATGATGAGCGCCCAGCAAATGGCTTCGATATCCGTTTTAATGGCTATCTACCGTCATATCCGGCATTAGGCGCCA
GCCGACCGTACTCAGTATGTTATTCTTTCTGATACTACTCGCGGGTCGTTTACCGAAGCTATAGGCAAAATTACCGATAGATGGCAGTATAGGCCGTAATCCGCGGT

intimin

2,250 2,260 2,270 2,280 2,290 2,300 2,310 2,320 2,330 2,340 2,350

AGCTGATATATGAGCAGTATTATGGTGATAATGTTGCTTTGTTTAATTCTGATAAGCTGCAATCGAATCCTGGTGCGGCGACCGTTGGTGTAAACTATACTCCGATT
TCGACTATATACTCGTCATAATACCACTATTACAACGAAACAAATTAAGACTATTCGACGTTAGCTTAGGACCACGCCGCTGGCAACCACATTTGATATGAGGCTAA

intimin

2,360 2,370 2,380 2,390 2,400 2,410 2,420 2,430 2,440 2,450 2,460

CCTCTGGTGACGATGGGGATCGATTACCGTCATGGTACGGGTAATGAAAATGATCTCCTTTACTCAATGCAGTTCCGTTATCAGTTTGATAAATCGTGGTCTCAGCA
GGAGACCACTGCTACCCCTAGCTAATGGCAGTACCATGCCCATTACTTTTACTAGAGGAAATGAGTTACGTCAAGGCAATAGTCAAACTATTTAGCACCAGAGTCGT

intimin

2,470 2,480 2,490 2,500 2,510 2,520 2,530 2,540 2,550 2,560

AATTGAACCACAGTATGTTAACGAGTTAAGAACATTATCAGGCAGCCGTTACGATCTGGTTCAGCGTAATAACAATATTATTCTGGAGTACAAGAAGCAGGATATTC
TTAACTTGGTGTCATACAATTGCTCAATTCTTGTAATAGTCCGTCGGCAATGCTAGACCAAGTCGCATTATTGTTATAATAAGACCTCATGTTCTTCGTCCTATAAG

intimin

2,570 2,580 2,590 2,600 2,610 2,620 2,630 2,640 2,650 2,660 2,670

TTTCTCTGAATATTCCGCATGATATTAATGGTACTGAACACAGTACGCAGAAGATTCAGTTGATCGTTAAGAGCAAATACGGTCTGGATCGTATCGTCTGGGATGAT
AAAGAGACTTATAAGGCGTACTATAATTACCATGACTTGTGTCATGCGTCTTCTAAGTCAACTAGCAATTCTCGTTTATGCCAGACCTAGCATAGCAGACCCTACTA

intimin

2,680 2,690 2,700 2,710 2,720 2,730 2,740 2,750 2,760 2,770 2,780

AGTGCATTACGCAGTCAGGGCGGTCAGATTCAGCATAGCGGAAGCCAAAGCGCACAAGACTACCAGGCTATTTTGCCTGCTTATGTGCAAGGTGGCAGCAATATTTA
TCACGTAATGCGTCAGTCCCGCCAGTCTAAGTCGTATCGCCTTCGGTTTCGCGTGTTCTGATGGTCCGATAAAACGGACGAATACACGTTCCACCGTCGTTATAAAT

intimin

2,790 2,800 2,810 2,820 2,830 2,840 2,850 2,860 2,870 2,880

TAAAGTGACGGCTCGCGCCTATGACCGTAATGGCAATAGCTCTAACAATGTACAGCTTACTATTACCGTTCTGTCGAATGGTCAAGTTGTCGACCAGGTTGGGGTAA
ATTTCACTGCCGAGCGCGGATACTGGCATTACCGTTATCGAGATTGTTACATGTCGAATGATAATGGCAAGACAGCTTACCAGTTCAACAGCTGGTCCAACCCCATT

intimin

2,890 2,900 2,910 2,920 2,930 2,940 2,950 2,960 2,970 2,980 2,990

CGGACTTTACGGCGGATAAGACTTCGGCTAAAGCGGATAACGCCGATACCATTACTTATACCGCGACGGTGAAAAAGAATGGGGTAGCTCAGGCTAATGTCCCTGTT
GCCTGAAATGCCGCCTATTCTGAAGCCGATTTCGCCTATTGCGGCTATGGTAATGAATATGGCGCTGCCACTTTTTCTTACCCCATCGAGTCCGATTACAGGGACAA

intimin

3,000 3,010 3,020 3,030 3,040 3,050 3,060 3,070 3,080 3,090 3,100

TCATTTAATATTGTTTCAGGAACTGCAACTCTTGGGGCAAATAGTGCCAAAACGGATGCTAACGGTAAGGCAACCGTAACGTTGAAGTCGAGTACGCCAGGACAGGT
AGTAAATTATAACAAAGTCCTTGACGTTGAGAACCCCGTTTATCACGGTTTTGCCTACGATTGCCATTCCGTTGGCATTGCAACTTCAGCTCATGCGGTCCTGTCCA

intimin

3,110 3,120 3,130 3,140 3,150 3,160 3,170 3,180 3,190 3,200 3,210

CGTCGTGTCTGCTAAAACCGCGGAGATGACTTCAGCACTTAATGCCAGTGCGGTTATATTTTTTGATGGTGCGACTAGATCGCAGCGTCAACTGGTTGAATCTGGCG
GCAGCACAGACGATTTTGGCGCCTCTACTGAAGTCGTGAATTACGGTCACGCCAATATAAAAAACTACCACGCTGATCTAGCGTCGCAGTTGACCAACTTAGACCGC

S Q R Q L V E S G

antiOmpA-L (Nb39)

intimin

nanobody 39...ainst OmpA

3,220 3,230 3,240 3,250 3,260 3,270 3,280 3,290 3,300 3,310

GGGGTCTGGTCCATACTGGTGGCTCATTAAAACTTTCGTGCGTCCCGAATGGTAGCATCTTCAACTTCAACCCGATGGGTTGGTATCGTCAAGTTTCGGGCCAACAG
CCCCAGACCAGGTATGACCACCGAGTAATTTTGAAAGCACGCAGGGCTTACCATCGTAGAAGTTGAAGTTGGGCTACCCAACCATAGCAGTTCAAAGCCCGGTTGTC
G G L V H T G G S L K L S C V P N G S I F N F N P M G W Y R Q V S G Q Q
antiOmpA-L (Nb39)
nanobody 39 against OmpA
3,320 3,330 3,340 3,350 3,360 3,370 3,380 3,390 3,400 3,410 3,420

CGTGAACTTGTCGCTACCCTGACGCGTGACGGTGTGGAAAACTATGCGTCCTCGGTGAAAGGCCGGTTTACGATTAGTCGTGATTCCGCGAAAAATACTCTCTATCT
GCACTTGAACAGCGATGGGACTGCGCACTGCCACACCTTTTGATACGCAGGAGCCACTTTCCGGCCAAATGCTAATCAGCACTAAGGCGCTTTTTATGAGAGATAGA
R E L V A T L T R D G V E N Y A S S V K G R F T I S R D S A K N T L Y L
antiOmpA-L (Nb39)
nanobody 39 against OmpA
3,430 3,440 3,450 3,460 3,470 3,480 3,490 3,500 3,510 3,520 3,530

GCAGATGACGGATGTTAAACCGGGTGACGCGGCGGTCTATATCTGTCACGCAAATTATCGTATTGGCCGTAATGACCTTCCTGTGTGGGGCAAAGGGACACCAGTTA
CGTCTACTGCCTACAATTTGGCCCACTGCGCCGCCAGATATAGACAGTGCGTTTAATAGCATAACCGGCATTACTGGAAGGACACACCCCGTTTCCCTGTGGTCAAT
Q M T D V K P G D A A V Y I C H A N Y R I G R N D L P V W G K G T P V
antiOmpA-L (Nb39)
nanobody 39 against OmpA
3,540 3,550 3,560 3,570 3,580 3,590 3,600 3,610 3,620 3,630

CGGTCAGCTAATACTtcacacaggaaacctactaaATGAAGAATAACAAACTTGGGTTACGGATCGGTATCGTAGGGGGTGGCATTTCAGGTGTCGCCTTGGCATTG
GCCAGTCGATTATGAagtgtgtcctttggatgattTACTTCTTATTGTTTGAACCCAATGCCTAGCCATAGCATCCCCCACCGTAAAGTCCACAGCGGAACCGTAAC
T V S *
antiOm...
na...A
nahG
3,640 3,650 3,660 3,670 3,680 3,690 3,700 3,710 3,720 3,730 3,740

GAACTGTGCCGTTACTCACACATCCAGGTGCAACTGTTCGAGGCAGCCCCGGCATTTGGGGAGGTCGGCGCCGGTGTCAGTTTCGGTCCGAACGCTGTGCGGGCCAT
CTTGACACGGCAATGAGTGTGTAGGTCCACGTTGACAAGCTCCGTCGGGGCCGTAAACCCCTCCAGCCGCGGCCACAGTCAAAGCCAGGCTTGCGACACGCCCGGTA
nahG
3,750 3,760 3,770 3,780 3,790 3,800 3,810 3,820 3,830 3,840 3,850

TGTCGGCTTAGGCCTGGGGGAGGCATATTTACAGGTGGCCGATCGTACATCAGAGCCATGGGAGGATGTGTGGTTTGAGTGGCGGCGTGGCTCAGACGCATCGTACC
ACAGCCGAATCCGGACCCCCTCCGTATAAATGTCCACCGGCTAGCATGTAGTCTCGGTACCCTCCTACACACCAAACTCACCGCCGCACCGAGTCTGCGTAGCATGG
nahG
3,860 3,870 3,880 3,890 3,900 3,910 3,920 3,930 3,940 3,950

TTGGCGCGACAATTGCGCCAGGCGTAGGCCAGTCTAGCGTACACCGGGCAGATTTCATCGATGCGCTGGTAACGCATCTCCCAGAAGGTATTGCACAGTTCGGTAAG
AACCGCGCTGTTAACGCGGTCCGCATCCGGTCAGATCGCATGTGGCCCGTCTAAAGTAGCTACGCGACCATTGCGTAGAGGGTCTTCCATAACGTGTCAAGCCATTC
nahG
3,960 3,970 3,980 3,990 4,000 4,010 4,020 4,030 4,040 4,050 4,060

CGGGCAACGCAAGTGGAACAGCAGGGGGGCGAAGTACAAGTGTTATTTACAGACGGTACAGAATATCGTTGTGATTTGCTGATTGGGGCTGATGGTATCAAATCGGC
GCCCGTTGCGTTCACCTTGTCGTCCCCCCGCTTCATGTTCACAATAAATGTCTGCCATGTCTTATAGCAACACTAAACGACTAACCCCGACTACCATAGTTTAGCCG
nahG
4,070 4,080 4,090 4,100 4,110 4,120 4,130 4,140 4,150 4,160 4,170

TCTGCGGTCGCATGTACTTGAGGGGCAAGGGCTTGCGCCGCAGGTCCCACGCTTCTCTGGCACCTGCGCATATCGGGGGATGGTGGACTCCTTGCATCTTCGTGAAG
AGACGCCAGCGTACATGAACTCCCCGTTCCCGAACGCGGCGTCCAGGGTGCGAAGAGACCGTGGACGCGTATAGCCCCCTACCACCTGAGGAACGTAGAAGCACTTC
nahG
4,180 4,190 4,200 4,210 4,220 4,230 4,240 4,250 4,260 4,270 4,280

CCTATCGCGCTCATGGGATCGACGAACATCTGGTTGACGTGCCTCAGATGTACCTGGGGCTCGATGGCCATATTTTAACTTTCCCGGTCCGTAATGGTGGGATTATT
GGATAGCGCGAGTACCCTAGCTGCTTGTAGACCAACTGCACGGAGTCTACATGGACCCCGAGCTACCGGTATAAAATTGAAAGGGCCAGGCATTACCACCCTAATAA
nahG
4,290 4,300 4,310 4,320 4,330 4,340 4,350 4,360 4,370 4,380

AATGTCGTTGCATTCATCTCGGACCGGTCTGAGCCGAAACCGACGTGGCCTGCTGATGCGCCGTGGGTACGTGAAGCATCCCAACGGGAGATGCTTGACGCTTTTGC
TTACAGCAACGTAAGTAGAGCCTGGCCAGACTCGGCTTTGGCTGCACCGGACGACTACGCGGCACCCATGCACTTCGTAGGGTTGCCCTCTACGAACTGCGAAAACG
nahG
4,390 4,400 4,410 4,420 4,430 4,440 4,450 4,460 4,470 4,480 4,490

CGGTTGGGGCGATGCTGCACGTGCCCTGTTAGAGTGTATTCCTGCCCCAACGCTGTGGGCATTACACGATCTTGCTGAACTTCCTGGGTACGTGCATGGCCGGGTGG
GCCAACCCCGCTACGACGTGCACGGGACAATCTCACATAAGGACGGGGTTGCGACACCCGTAATGTGCTAGAACGACTTGAAGGACCCATGCACGTACCGGCCCACC
nahG
4,500 4,510 4,520 4,530 4,540 4,550 4,560 4,570 4,580 4,590 4,600

TTCTCATTGGCGACGCGGCACACGCAATGCTTCCACACCAAGGCGCCGGGGCTGGGCAAGGGCTGGAAGATGCCTACTTCCTCGCCCGCTTACTTGGTGACACACAA
AAGAGTAACCGCTGCGCCGTGTGCGTTACGAAGGTGTGGTTCCGCGGCCCCGACCCGTTCCCGACCTTCTACGGATGAAGGAGCGGGCGAATGAACCACTGTGTGTT
nahG
4,610 4,620 4,630 4,640 4,650 4,660 4,670 4,680 4,690 4,700

GCCGACGCCGGTAATCTTGCAGAACTTCTTGAGGCCTACGATGATTTACGCCGGCCACGCGCCTGTCGGGTACAACAAACGAGTTGGGAGACCGGCGAGTTATATGA
CGGCTGCGGCCATTAGAACGTCTTGAAGAACTCCGGATGCTACTAAATGCGGCCGGTGCGCGGACAGCCCATGTTGTTTGCTCAACCCTCTGGCCGCTCAATATACT
nahG
4,710 4,720 4,730 4,740 4,750 4,760 4,770 4,780 4,790 4,800 4,810

GTTACGCGACCCTGTTGTGGGTGCAAACGAACAGCTTCTTGGCGAGAACTTAGCAACGCGGTTTGACTGGTTGTGGAATCACGACCTTGACACTGATTTGGCAGAAG
CAATGCGCTGGGACAACACCCACGTTTGCTTGTCGAAGAACCGCTCTTGAATCGTTGCGCCAAACTGACCAACACCTTAGTGCTGGAACTGTGACTAAACCGTCTTC
nahG
4,820 4,830 4,840 4,850 4,860 4,870 4,880 4,890 4,900 4,910 4,920

CTCGCGCACGGTTGGGCTGGGAGCATGGTGGTGGCGCCTTACGGCAGGGCTAAAAAGAGGAGAAAGGTACCatggcgagtagcgaagacgttatcaaagagttc
GAGCGCGTGCCAACCCGACCCTCGTACCACCACCACCGCGGAATGCCGTCCCGATTTTTCTCCTCTTTCCATGGtaccgctcatcgcttctgcaatagtttctcaag

nahG RBS mRFP1

4,930 4,940 4,950 4,960 4,970 4,980 4,990 5,000 5,010 5,020

atgcgtttcaaagttcgtatggaaggttccgttaacggtcacgagttcgaaatcgaaggtgaaggtgaaggtcgtccgtacgaaggtacccagaccgctaaactgaa
tacgcaaagtttcaagcataccttccaaggcaattgccagtgctcaagctttagcttccacttccacttccagcaggcatgcttccatgggtctggcgatttgactt

mRFP1

5,030 5,040 5,050 5,060 5,070 5,080 5,090 5,100 5,110 5,120 5,130

agttaccaaaggtggtccgctgccgttcgcttgggacatcctgtccccgcagttccagtacggttccaaagcttacgttaaacacccggctgacatcccggactacc
tcaatggtttccaccaggcgacggcaagcgaaccctgtaggacaggggcgtcaaggtcatgccaaggtttcgaatgcaatttgtgggccgactgtagggcctgatgg

mRFP1

5,140 5,150 5,160 5,170 5,180 5,190 5,200 5,210 5,220 5,230 5,240

tgaaactgtccttcccggaaggtttcaaatgggaacgtgttatgaacttcgaagacggtggtgttgttaccgttacccaggactcctccctgcaagacggtgagttc
actttgacaggaagggccttccaaagtttacccttgcacaatacttgaagcttctgccaccacaacaatggcaatgggtcctgaggagggacgttctgccactcaag

mRFP1

5,250 5,260 5,270 5,280 5,290 5,300 5,310 5,320 5,330 5,340 5,350

atctacaaagttaaactgcgtggtaccaacttcccgtccgacggtccggttatgcagaaaaaaaccatgggttgggaagcttccaccgaacgtatgtacccggaaga
tagatgtttcaatttgacgcaccatggttgaagggcaggctgccaggccaatacgtctttttttggtacccaacccttcgaaggtggcttgcatacatgggccttct

mRFP1

5,360 5,370 5,380 5,390 5,400 5,410 5,420 5,430 5,440 5,450

cggtgctctgaaaggtgaaatcaaaatgcgtctgaaactgaaagacggtggtcactacgacgctgaagttaaaaccacctacatggctaaaaaaccggttcagctgc
gccacgagactttccactttagttttacgcagactttgactttctgccaccagtgatgctgcgacttcaattttggtggatgtaccgattttttggccaagtcgacg

mRFP1

5,460 5,470 5,480 5,490 5,500 5,510 5,520 5,530 5,540 5,550 5,560

cgggtgcttacaaaaccgacatcaaactggacatcacctcccacaacgaagactacaccatcgttgaacagtacgaacgtgctgaaggtcgtcactccaccggtgct
gcccacgaatgttttggctgtagtttgacctgtagtggagggtgttgcttctgatgtggtagcaacttgtcatgcttgcacgacttccagcagtgaggtggccacga

mRFP1

5,570 5,580 5,590 5,600 5,610 5,620 5,630 5,640 5,650 5,660 5,670

taaaagcttgacctgtgaagtgaaaaatggcgcacattgtgcgacatttttttgtctgccgtttaccgctactgcgtcacggatccccacgcgccctgtagcggcg
attttcgaactggacacttcactttttaccgcgtgtaacacgctgtaaaaaaacagacggcaaatggcgatgacgcagtgcctaggggtgcgcgggacatcgccgc

f1 ori

5,680 5,690 5,700 5,710 5,720 5,730 5,740 5,750 5,760 5,770

cattaagcgcggcgggtgtggtggttacgcgcagcgtgaccgctacacttgccagcgccctagcgcccgctcctttcgctttcttcccttcctttctcgccacgttc
gtaattcgcgccgcccacaccaccaatgcgcgtcgcactggcgatgtgaacggtcgcgggatcgcgggcgaggaaagcgaaagaagggaaggaaagagcggtgcaag

f1 ori

5,780 5,790 5,800 5,810 5,820 5,830 5,840 5,850 5,860 5,870 5,880

gccggctttccccgtcaagctctaaatcggggcatccctttagggttccgatttagtgctttacggcacctcgaccccaaaaaacttgattagggtgatggttcacg
cggccgaaaggggcagttcgagatttagccccgtagggaaatcccaaggctaaatcacgaaatgccgtggagctggggttttttgaactaatcccactaccaagtgc

f1 ori

5,890 5,900 5,910 5,920 5,930 5,940 5,950 5,960 5,970 5,980 5,990

tagtgggccatcgccctgatagacggtttttcgccctttgacgttggagtccacgttctttaatagtggactcttgttccaaactggaacaacactcaaccctatct
atcacccggtagcgggactatctgccaaaaagcgggaaactgcaacctcaggtgcaagaaattatcacctgagaacaaggtttgaccttgttgtgagttgggataga

f1 ori

6,000 6,010 6,020 6,030 6,040 6,050 6,060 6,070 6,080 6,090

cggtctattcttttgatttataagggattttgccgatttcggcctattggttaaaaaatgagctgatttaacaaaaatttaacgcgaattttaacaaaatattaacg
gccagataagaaaactaaatattccctaaaacggctaaagccggataaccaattttttactcgactaaattgtttttaaattgcgcttaaaattgttttataattgc

f1 ori

6,100 6,110 6,120 6,130 6,140 6,150 6,160 6,170 6,180 6,190 6,200

tttacaatttcaggtggcacttttcggggaaatgtgcgcggaacccctatttgtttatttttctaaatacattcaaatatgtatccgctcatgtcgagacgttgggt
aaatgttaaagtccaccgtgaaaagcccctttacacgcgccttggggataaacaaataaaaagatttatgtaagtttatacataggcgagtacagctctgcaaccca

f1...i

6,210 6,220 6,230 6,240 6,250 6,260 6,270 6,280 6,290 6,300 6,310

gaggttccaactttcaccataatgaaataagatcactaccgggcgtatttttttgagttatcgagattttcaggagctaaggaagctaaaatggagaaaaaaatcact
ctccaaggttgaaagtggtattactttattctagtgatggcccgcataaaaaaactcaatagctctaaaagtcctcgattccttcgattttacctctttttttagtga

CmR

6,320 6,330 6,340 6,350 6,360 6,370 6,380 6,390 6,400 6,410 6,420

ggatataccaccgttgatatatcccaatggcatcgtaaagaacattttgaggcatttcagtcagttgctcaatgtacctataaccagaccgttcagctggatattac
cctatatggtggcaactatatagggttaccgtagcatttcttgtaaaactccgtaaagtcagtcaacgagttacatggatattggtctggcaagtcgacctataatg

CmR

6,430 6,440 6,450 6,460 6,470 6,480 6,490 6,500 6,510 6,520

ggcctttttaaagaccgtaaagaaaaataagcacaagttttatccggcctttattcacattcttgcccgcctgatgaatgctcatccggagttccgtatggcaatga
ccggaaaaatttctggcatttctttttattcgtgttcaaaataggccggaaataagtgtaagaacgggcggactacttacgagtaggcctcaaggcataccgttact

CmR

6,530 6,540 6,550 6,560 6,570 6,580 6,590 6,600 6,610 6,620 6,630

aagacggtgagctggtgatatgggatagtgttcacccttgttacaccgttttccatgagcaaactgaaacgttttcatcgctctggagtgaataccacgacgatttc
ttctgccactcgaccactataccctatcacaagtgggaacaatgtggcaaaaggtactcgtttgactttgcaaaagtagcgagacctcacttatggtgctgctaaag

CmR

6,640 6,650 6,660 6,670 6,680 6,690 6,700 6,710 6,720 6,730 6,740

cggcagtttctacacatatattcgcaagatgtggcgtgttacggtgaaaacctggcctatttccctaaagggtttattgagaatatgtttttcgtctcagccaatcc
gccgtcaaagatgtgtatataagcgttctacaccgcacaatgccacttttggaccggataaagggatttcccaaataactcttatacaaaaagcagagtcggttagg

CmR

6,750 6,760 6,770 6,780 6,790 6,800 6,810 6,820 6,830 6,840

ctgggtgagtttcaccagttttgatttaaacgtggccaatatggacaacttcttcgcccccgttttcaccatgggcaaatattatacgcaaggcgacaaggtgctga
gacccactcaaagtggtcaaaactaaatttgcaccggttatacctgttgaagaagcgggggcaaaagtggtacccgtttataatatgcgttccgctgttccacgact

CmR

6,850 6,860 6,870 6,880 6,890 6,900 6,910 6,920 6,930 6,940 6,950

tgccgctggcgattcaggttcatcatgccgtctgtgatggcttccatgtcggcagaatgcttaatgaattacaacagtactgcgatgagtggcagggcggggcgtaa
acggcgaccgctaagtccaagtagtacggcagacactaccgaaggtacagccgtcttacgaattacttaatgttgtcatgacgctactcaccgtcccgccccgcatt

CmR

6,960 6,970 6,980 6,990 7,000 7,010 7,020 7,030 7,040 7,050 7,060

tttttttaaggcagttattggtgcccttaaacgcctggtgctacgcctgaataagtgataataagcggatgaatggcagaaattcgaaagcaaattcgacccggtcg
aaaaaaattccgtcaataaccacgggaatttgcggaccacgatgcggacttattcactattattcgcctacttaccgtctttaagctttcgtttaagctgggccagc

7,070 7,080 7,090 7,100 7,110 7,120 7,130 7,140 7,150 7,160

tcggttcagggcagggtcgttaaatagccgcttatgtctattgctggtttaccggtttattgactaccggaagcagtgtgaccgtgtgcttctcaaatgcctgaggc
agccaagtcccgtcccagcaatttatcggcgaatacagataacgaccaaatggccaaataactgatggccttcgtcacactggcacacgaagagtttacggactccg

7,170 7,180 7,190 7,200 7,210 7,220 7,230 7,240 7,250 7,260 7,270

cagtttgctcaggctctccccgtggaggtaataattgctcgacatgaccaaaatcccttaacgtgagttttcgttccactgagcgtcagaccccgtagaaaagatca
gtcaaacgagtccgagaggggcacctccattattaacgagctgtactggttttagggaattgcactcaaaagcaaggtgactcgcagtctggggcatcttttctagt

7,280 7,290 7,300 7,310 7,320 7,330 7,340 7,350 7,360 7,370 7,380

aaggatcttcttgagatcctttttttctgcgcgtaatctgctgcttgcaaacaaaaaaaccaccgctaccagcggtggtttgtttgccggatcaagagctaccaact
ttcctagaagaactctaggaaaaaaagacgcgcattagacgacgaacgtttgtttttttggtggcgatggtcgccaccaaacaaacggcctagttctcgatggttga

ori

7,390 7,400 7,410 7,420 7,430 7,440 7,450 7,460 7,470 7,480 7,490

ctttttccgaaggtaactggcttcagcagagcgcagataccaaatactgtccttctagtgtagccgtagttaggccaccacttcaagaactctgtagcaccgcctac
gaaaaaggcttccattgaccgaagtcgtctcgcgtctatggtttatgacaggaagatcacatcggcatcaatccggtggtgaagttcttgagacatcgtggcggatg

ori

7,500 7,510 7,520 7,530 7,540 7,550 7,560 7,570 7,580 7,590

atacctcgctctgctaatcctgttaccagtggctgctgccagtggcgataagtcgtgtcttaccgggttggactcaagacgatagttaccggataaggcgcagcggt
tatggagcgagacgattaggacaatggtcaccgacgacggtcaccgctattcagcacagaatggcccaacctgagttctgctatcaatggcctattccgcgtcgcca

ori

7,600 7,610 7,620 7,630 7,640 7,650 7,660 7,670 7,680 7,690 7,700

cgggctgaacggggggttcgtgcacacagcccagcttggagcgaacgacctacaccgaactgagatacctacagcgtgagctatgagaaagcgccacgcttcccgaa
gcccgacttgccccccaagcacgtgtgtcgggtcgaacctcgcttgctggatgtggcttgactctatggatgtcgcactcgatactctttcgcggtgcgaagggctt

ori

7,710 7,720 7,730 7,740 7,750 7,760 7,770 7,780 7,790 7,800 7,810

gggagaaaggcggacaggtatccggtaagcggcagggtcggaacaggagagcgcacgagggagcttccagggggaaacgcctggtatctttatagtcctgtcgggtt
ccctctttccgcctgtccataggccattcgccgtcccagccttgtcctctcgcgtgctccctcgaaggtccccctttgcggaccatagaaatatcaggacagcccaa

ori

7,820 7,830 7,840 7,850 7,860 7,870 7,880 7,890 7,900 7,910

tcgccacctctgacttgagcgtcgatttttgtgatgctcgtcaggggggcggagcctatggaaaaacgccagcaacgcggcctttttacggttcctggccttttgct
agcggtggagactgaactcgcagctaaaaacactacgagcagtccccccgcctcggatacctttttgcggtcgttgcgccggaaaaatgccaaggaccggaaaacga

ori

7,920 7,930 7,940 7,950 7,960 7,970 7,980 7,990 8,000 8,010 8,020

ggccttttgctcacatg
ccggaaaacgagtgtac

8,030 8,040
